# The effects of base rate neglect on sequential belief updating and real-world beliefs

**DOI:** 10.1371/journal.pcbi.1010796

**Published:** 2022-12-22

**Authors:** Brandon K. Ashinoff, Justin Buck, Michael Woodford, Guillermo Horga

**Affiliations:** 1 Department of Psychiatry, Columbia University, New York, NY, United States of America; 2 New York State Psychiatric Institute (NYSPI), New York, NY, United States of America; 3 Department of Neuroscience, Columbia University, New York, NY, United States of America; 4 Department of Economics, Columbia University, New York, NY, United States of America; Harvard University, UNITED STATES

## Abstract

Base-rate neglect is a pervasive bias in judgment that is conceptualized as underweighting of prior information and can have serious consequences in real-world scenarios. This bias is thought to reflect variability in inferential processes but empirical support for a cohesive theory of base-rate neglect with sufficient explanatory power to account for longer-term and real-world beliefs is lacking. A Bayesian formalization of base-rate neglect in the context of sequential belief updating predicts that belief trajectories should exhibit dynamic patterns of dependence on the order in which evidence is presented and its consistency with prior beliefs. To test this, we developed a novel ‘urn-and-beads’ task that systematically manipulated the order of colored bead sequences and elicited beliefs via an incentive-compatible procedure. Our results in two independent online studies confirmed the predictions of the sequential base-rate neglect model: people exhibited beliefs that are more influenced by recent evidence and by evidence inconsistent with prior beliefs. We further found support for a noisy-sampling inference model whereby base-rate neglect results from rational discounting of noisy internal representations of prior beliefs. Finally, we found that model-derived indices of base-rate neglect—including noisier prior representation—correlated with propensity for unusual beliefs outside the laboratory. Our work supports the relevance of Bayesian accounts of sequential base-rate neglect to real-world beliefs and hints at strategies to minimize deleterious consequences of this pervasive bias.

## Author summary

Base-rate neglect is a common bias in judgment, a bias defined by a tendency to underuse older information when forming a new belief. This bias can have serious consequences in the real world. Base-rate neglect is often cited as a source of errors in medical and legal decisions, and in many other socially relevant contexts. Despite its broad societal relevance, it is unclear whether current theories capture the expression of base-rate neglect in sequential belief formation, and perhaps more crucially why people have this bias in the first place. In this paper, we find support for a model that describes how base-rate neglect influences belief formation over time, showing that people behave in a way that matches theoretical predictions. Knowing how base-rate neglect influences beliefs over time suggests possible strategies that could be implemented in the future to minimize its impact. We also find support for a model which may explain why people exhibit base-rate neglect in the first place. This model suggests that people’s representation of older information in the brain is noisy and that it is therefore rational to underuse this older information to some extent depending on how noisy or unreliable its representation is. Finally, we show that our measures of base-rate neglect and noise in the representation of older information correlate with variation in real-world belief oddity, suggesting that these models capture belief-formation processes likely to dictate functioning in real-world settings.

## Introduction

Accurate judgments in the face of equivocal—even nearly unequivocal—evidence depend critically upon incorporating prior knowledge about the probability of different scenarios, often referred to as their base rate. Consider a doctor deciding whether a patient has a rare disease (i.e., one with a very low base rate). She orders a diagnostic test that is 99% accurate, and it comes back positive. Intuitively, you may reason it is likely that the patient has the disease. However, in this case a positive test result is actually associated with a very low probability of the disease. In this scenario, neglecting to account for the base rate may lead to a misdiagnosis and serious negative outcomes. This example illustrates the pervasive bias known as base-rate neglect [1–5] and its potential real-world consequences. Far from merely being a hypothetical example, studies have shown that diagnosticians tend to discount known disease rates [6] and relevant medical history [7–9]. Research into base-rate neglect in other areas further highlights its broad societal relevance: for example, base-rate neglect leads to an overestimation of success in environmentally relevant pursuits [10], inaccurate judgments about job candidates [11], and errors in legal decision-making [12–14]. However, despite its importance, the mechanisms governing base-rate neglect and its longer-term effects on human belief updating are poorly understood.

Starting with foundational work on base-rate neglect [5], previous theoretical [1,2,5,15–17] work has formalized base-rate neglect in a Bayesian framework as an underweighting of prior beliefs, or beliefs summarizing previously observed information into the *a priori* probability of a state or event without additional information—mathematically equivalent to its base rate [2]. This Bayesian framework extends to belief updating in sequential contexts [2,15,16] that encompass and go beyond classically studied ‘one-shot’ scenarios, and which arguably have more ecological validity[18]. Crucially, in the context of sequential belief updating, under a Bayesian model where underweighted prior beliefs are iteratively updated upon observation of new evidence samples, theoretical work indicates that base-rate neglect should impact belief-updating dynamics in a lawful manner, simultaneously producing two main effects [2,15,16]. First, base-rate neglect in this context, henceforth referred to as ‘sequential base-rate neglect’, should result in more reliance on newer information to form beliefs—a recency bias. Second, it should result in a specific form of prior-dependent belief updating—with smaller updates to prior-consistent evidence—that imposes a lower boundary on belief certainty over the long run. These two model predictions imply that the beliefs of a sequential base-rate neglecter, unlike those of an unbiased observer, should critically depend on the order in which evidence is presented *and* reach different levels of certainty even when presented with the same amount of evidence. Importantly, these theoretical predictions have not been jointly or systematically tested in empirical studies.

Previous empirical work is broadly consistent with the notion that base-rate neglect coexists with a recency bias [19–31]. However, whether the degree of base-rate neglect that individuals exhibit is commensurate with both the recency bias and the prior-dependent belief updating in a way that aligns with the abovementioned theoretical predictions remains unknown. Testing these predictions would ideally require a sequential belief-updating paradigm that incentivizes true beliefs, has sufficient evidence samples, is conducive to quantitative analysis, and systematically manipulates evidence order. In contrast, previous work on base-rate neglect has often used single [4,32] or short series of evidence samples [19–22,24–30,33,34], non-incentivized paradigms[4,35], or qualitative designs [19–23,33,34]. These concerns raise the possibility of various confounds [36] and further limit the ability of previous paradigms to characterize critical belief-updating dynamics predicted under sequential base-rate neglect.

**Fig 1:**
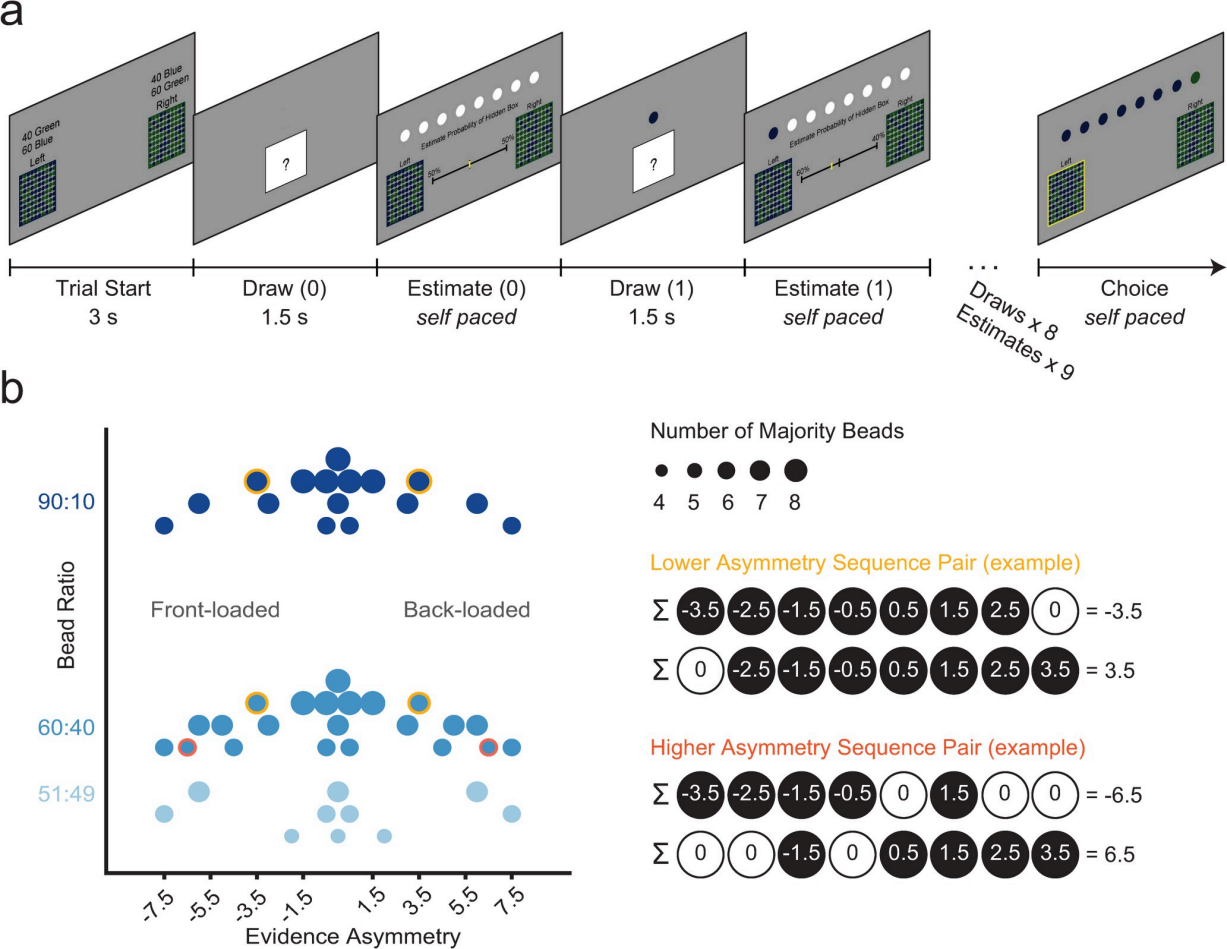
Task schematic. **(a)** Trial structure of the probability-estimates beads task. Participants are first shown two boxes, a ‘green box’ mostly filled with green beads and a ‘blue box’ mostly filled with blue beads. The ratio of blue to green beads (bead ratio) is shown. Participants are instructed that one of these two boxes, referred to as the “hidden box”, is selected at random, and that their task is to estimate which box is the hidden box based on beads drawn from it. Next, they are shown an obscured representation of the hidden box, but no bead is drawn. Participants then make a first probability estimate using a slider to indicate their perceived probability that the hidden box is either the blue or green box. White circles on top of the screen are used as placeholders to illustrate the remaining samples that will be drawn during the trial. After this first estimate, participants see the hidden box again but this time a bead rises out of the box. Participants are then asked to report a second probability estimate after seeing the first bead. The drawn bead replaces the leftmost available placeholder, starting a sequential visual record of beads drawn during a trial. This process of drawing and estimating repeats until participants have observed 8 samples and reported 9 estimates per trial. At the end of the trial, participants make a binary choice of the box they believe is the hidden box. After this choice, a new trial begins. **(b)** Task variable space showing bead-ratio conditions on the y-axis (each shown in a different shade of blue) and an evidence-order metric (evidence asymmetry) on the x-axis, with negative values indicating front-loading of majority beads (more majority beads, beads consistent with the identity of the hidden box, in the first half of the 8-bead sequence) and positive values indicating back-loading (more majority beads in the second half). The absolute value of the x axis corresponds to more extreme front- or back-loading (the most extreme being a sequence where 5 majority beads are all in the front or all in the back, respectively, and the least extreme being sequences where beads are evenly distributed around the middle). Larger circles reflect sequences with more majority beads. Sequences are organized in mirror-opposite pairs, with two example pairs shown on the right. Note that the examples illustrate majority beads as black and minority beads as white (albeit in the task majority beads were green or blue consistent with the identity of the hidden box in a given trial). Trials were selected to span the full range of the evidence asymmetry space while avoiding confounds with the bead-ratio condition (Fig 1B) and cumulative evidence (S1 Fig).

**Fig 2:**
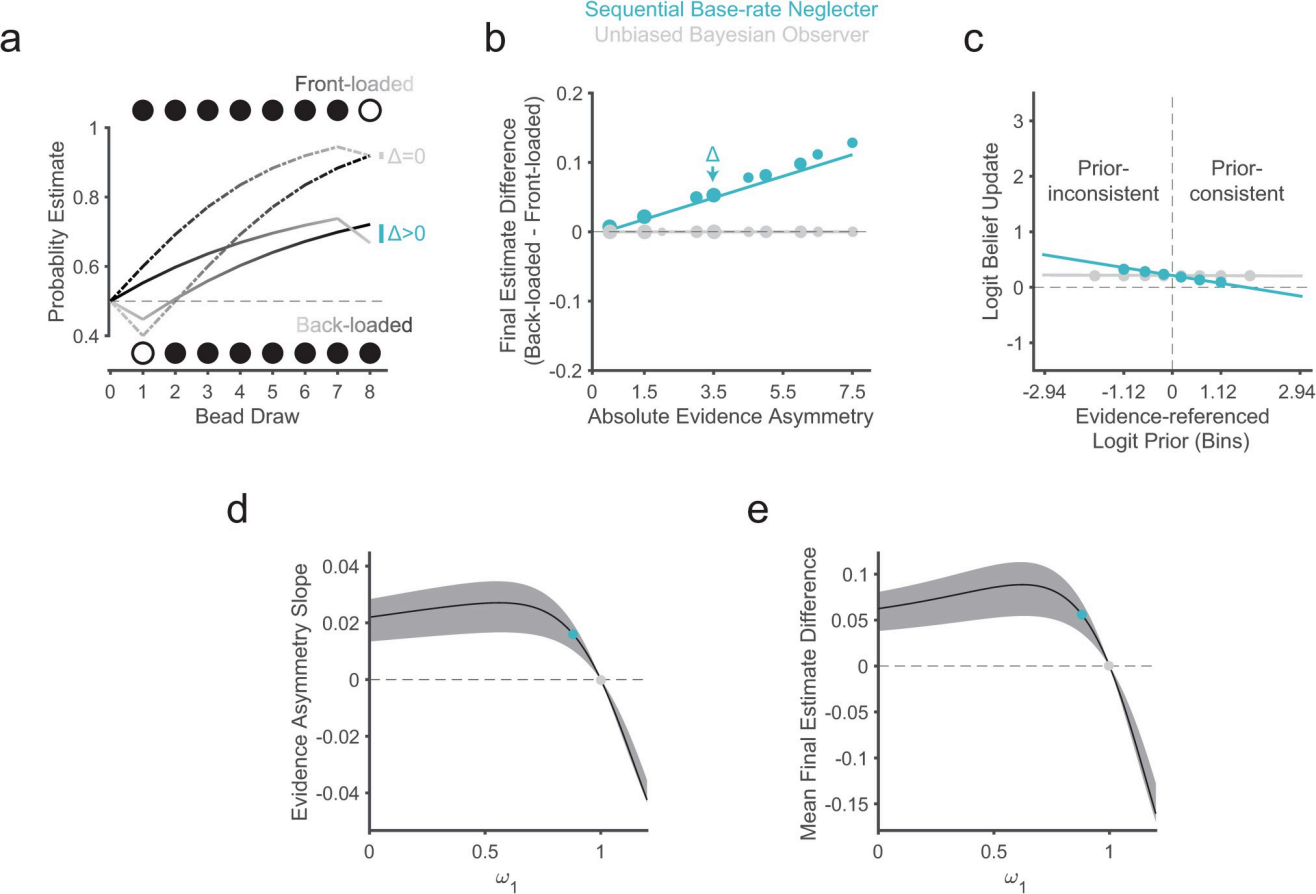
Model predictions for sequential base-rate neglect under the weighted Bayesian model. An agent with sequential base-rate neglect (*ω*_1_ < 1.00; for these simulations: *ω*_1_ = 0.88), in blue, is compared with a Bayesian ideal observer (*ω*_1_ = 1), in grey, on the 60:40 bead-ratio condition. Values of *ω*_2(60:40)_ are 0.51 (or between 0.31 and 0.66 in the shaded regions in panels d-e) consistent with observed mean values (and 25^th^ to 75^th^ percentile range) in our prior work with a similar beads task [31]. **(a)** Simulated sequential probability estimates for two mirror-opposite sequences for a base-rate neglecting agent (blue/solid) and the ideal Bayesian observer (grey/dashed). Majority beads are shown as black and minority beads as white for illustrative purposes. Belief trajectories for front-loaded sequences show a gradient from dark to light and those for the back-loaded sequences transition from light to dark **(b)** Simulation of the recency bias, defined as the difference between the final probability estimate after 8 beads between mirror-opposite pairs, as a function of the absolute evidence asymmetry of the pairs. As in Fig 1, larger circles reflect sequences with more majority beads. The fit line shows the fixed effect of absolute evidence asymmetry on the final estimate difference. The simulated base-rate neglecter shows higher estimates for back-loaded sequences (compared to their front-loaded mirror opposites), particularly for sequence pairs with more evidence asymmetry. This effect varies with evidence strength and is strongest in the 90:10 condition (S2A Fig). See S2E Fig for a simulation of an agent that overweights the prior. The lower-case delta shows the example from (a). **(c)** Simulation of the magnitude of logit-belief updates as a function of the prior with respect to the color of the current evidence. For illustrative purposes, the x-axis has been discretized into bins equivalent to 0.1 increments of prior beliefs in probability space. The y-axis represents the mean magnitude of the logit belief updates (the difference in the log-odds of the prior and the posterior belief). The Bayesian ideal observer has constant logit belief-updates. In contrast, the simulated base-rate neglecter shows logit-belief updates that depend upon the prior belief, with relatively larger updates for prior-inconsistent evidence (left of the vertical dashed line) and smaller for prior-consistent evidence (right) (see S2B Fig for a condition-wise simulation). The fit line reflects the fixed effect of logit-prior on the logit-belief update, which we refer to as prior-dependent belief updating. The model predicts main effects of logit-prior and bead-ratio condition, but no interaction S2B Fig. See S2D Fig for a simulation illustrating the distinct scaling effects of *ω*_2_ and S2F Fig for a simulation of an agent that overweights the prior. **(d)** Simulation demonstrating the predicted relationship between *ω*_1_ and the evidence asymmetry slope (blue fit line from b). **(e)** Simulation of the predicted relationship between *ω*_1_ and mean final estimate difference (average of blue data points in b). **(d,e)** The blue and grey dots show the values for the base-rate neglecting and Bayesian ideal observers simulated in **(a,b,c)**.

To address this gap in the literature, we developed and validated a novel probability-estimation task (Fig 1) adopting an “urn-and-beads” design [4,5,35], which we combined with computational modeling to test the predictions of the abovementioned weighted Bayesian framework of sequential base-rate neglect (Fig 2). Critically, our task systematically and selectively manipulated the evidence order of relatively long (8-sample) sequences and used an incentive-compatible belief-elicitation procedure [36–39].

Another outstanding issue in this literature relates to the underlying explanation for base-rate neglect and its (sub)optimality. While base-rate neglect can lead to adverse outcomes in one-shot scenarios [6–14], whether it can generally be considered suboptimal depends upon the theoretical framing. Early views framed base-rate neglect as a consequence of qualitative differences in the assessed representativeness [40] or relevance [5] of prior information relative to new evidence samples that are more immediately significant and thus disproportionately influential. An implication of these and related views [41–44] is that base-rate neglect results from a suboptimal heuristic strategy. In contrast, recent explanatory (functional or mechanistic) theories of belief updating [16,45,46] suggest that sequential base-rate neglect may represent an optimal response to perceived volatility in the environment [45,46] or to internal capacity limitations in the precision of information processing [16]. We thus evaluated these alternative accounts in order to advance a functional explanatory model of base-rate neglect [41].

Our results in two independent online studies confirm the joint predictions of the weighted Bayesian model on the dynamic hallmarks of sequential base-rate neglect. We additionally show that interindividual variability in sequential base-rate neglect measures derived from task behavior correlates with a tendency to hold odd beliefs outside the laboratory, further supporting the real-world relevance of sequential base-rate neglect. Finally, we provide initial support for a capacity-limited noisy-sampling model of sequential base-rate neglect that predicts the interindividual relationships with response variability observed in the data, supporting a framing of base-rate neglect as a rational response to an imprecise prior representation.

## Results

In each trial of the probability-estimates beads task (Fig 1A and Methods), participants had to estimate the probability of and eventually determine the identity of a “hidden” box, either a ‘blue box’ mostly filled with blue beads or a ‘green box’ mostly filled with green beads—with the bead ratios of blue to green beads being reciprocal and explicitly shown. The hidden box was randomly selected on each trial and remained the same for the duration of the trial. Participants were shown beads drawn from the hidden box one at a time. After each bead sample, and once before seeing any samples, participants had to report an estimate of the probability that the hidden box was the blue or the green box using a slider. At the end of a trial, after seeing 8 bead samples and reporting 9 estimates, participants made a binary choice about the hidden box. Critically, the task included various novel manipulations at the trial level to allow testing of the predictions of the weighted Bayesian model of sequential base-rate neglect (Fig 2): we systematically manipulated evidence strength (majority-to-minority ratio of bead colors in the hidden box, or ‘bead ratios’) and crucially the evidence order and symmetry of the 8-bead sequences, which we arranged as mirror-opposite sequence pairs presented in pseudorandom order (Fig 1B).

In the context of this task, sequential base-rate neglect is mathematically equivalent to underweighting of prior beliefs in a recursive weighted Bayesian model [1,2] (Methods) of the form: *logit*(*posterior*)_*d*_ = *ω*_1_∙*logit*(*prior*)_*d*_ + *ω*_2_∙*logit*(*likelihood*)_*d*_, where *d* is a given draw of an evidence (bead) sample, and *logit*(*prior*)_*d*_ *= logit*(*posterior*)_*d*−1_. In short, this model forms a posterior belief about the hidden box after a new sample is drawn (at *d*) by integrating a weighted prior probability of the hidden box (the belief before observing the new sample) and a weighted likelihood determined by the color of the new bead sample at draw *d* and the bead ratio for the trial. While the likelihood weight *ω*_2_ multiplicatively scales all evidence samples equally for a given bead ratio, the prior weight *ω*_1_ affects the evidence samples differentially as a function of the draw number *d*. In particular, prior underweighting (*ω*_1_<1) or sequential base-rate neglect, implies exponential discounting of older evidence samples as a function of number of draws into the past (i.e., the older the information, the more it is neglected or discounted). Theoretical predictions under this model suggest that sequential base-rate neglect should manifest as two main dynamic effects commensurate with the degree of base-rate neglect [2,15,16]: a recency bias (Fig 2B) and prior dependence in belief updating (Fig 2C).

Using this paradigm, we conducted two online studies which produced high-quality data consistent with in-person studies based on extensive quality checks (see *Online Data Quality* in Methods).

### Study 1

After exclusions (Methods), data for 151 participants were analyzed for Study 1.

#### Manipulation check

**Fig 3:**
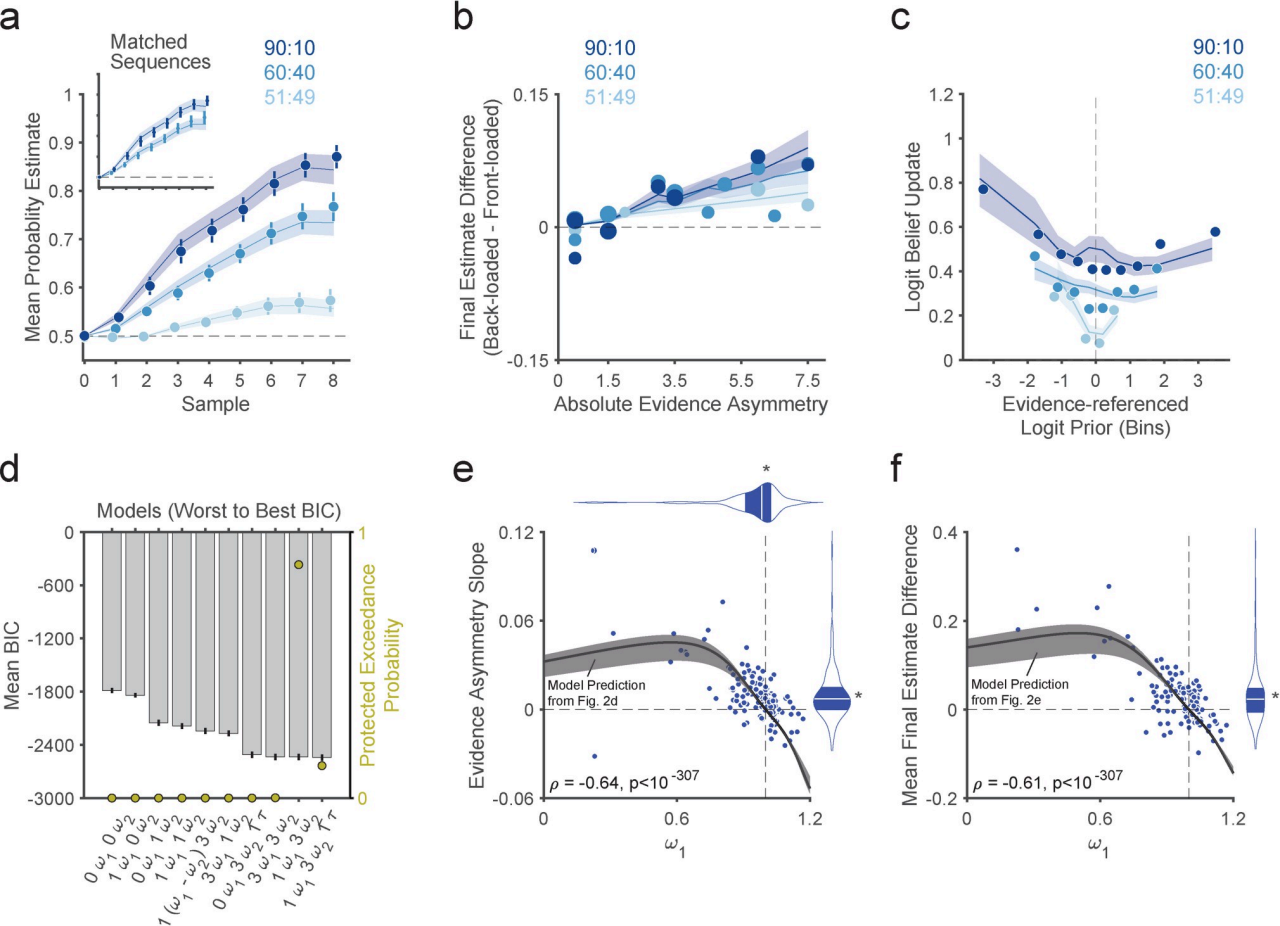
Study 1 participants show behavioral signatures of sequential base-rate neglect which scale with model-derived prior underweighting. **(a)** Group mean of average probability estimates over bead draws for each bead-ratio condition. Participants updated beliefs progressively toward the correct hidden box with steeper slopes for stronger evidence. The inset shows the same data limited to matched (identical) sequences for the 60:40 and 90:10 conditions. Solid lines and shaded regions reflect the mean and standard error of the mean (SEM) of the weighted Bayesian model fits across participants. **(b)** Group mean of final estimate difference as a function of evidence asymmetry. Each data point shows the difference in the probability estimate after 8 beads for a back-loaded and a front-loaded sequence comprising a mirror-opposite pair, with positive values indicating higher estimates for back-loaded sequences consistent with recency bias. Solid lines and shaded regions reflect the mean and SEM of the weighted Bayesian model fits. Consistent with model predictions (Fig 2B), the data shows a recency bias scaling with evidence asymmetry. **(c)** Group median of individual medians for the magnitude of logit-belief updates as a function of the logit prior with respect to the color of the current bead sample, divided by bead-ratio condition. The x-axis is discretized into bins equivalent to 0.1 increments of the prior belief in probability space (with a lower limit of 0.01 and an upper limit of 0.99; data only binned for visualization). The y-axis represents the magnitude of the logit-belief updates (the difference in the log-odds of the prior and posterior beliefs). Solid lines and shaded regions reflect medians and 95% bootstrapped confidence intervals of the weighted Bayesian model fits. Although not displayed for visual clarity, the confidence intervals for the raw data overlap substantially with the model fits. For visualization only, we excluded extreme outlier or noisy data points (logit belief updates > 2, individual median values based on less than 3 data points for a given bin, group median values based on less than 25% of individuals) for a total exclusion of 6.96% of the data. Consistent with model predictions (Fig 2C), the data shows prior-dependent belief-updating with less updating for prior-consistent evidence (right of the vertical dashed line; i.e. an overall negative slope). Note that at the group level this effect appears to be non-monotonic (with slightly positive slope towards the rightmost end) due to aggregation of data across individuals with different *ω*_1_ values, since individuals with *ω*_1_ > 1 are predicted to have and exhibit more updating to prior-consistent evidence (i.e., positive slopes; S2 Fig). **(d)** Formal model comparison for data from study 1. We compared 10 different models as in our previous work [31]. Each model is defined by its free parameters, which are reflected on the x-axis. See S28 Table for details. The winning model was defined as the model with the highest protected exceedance probability, which was the same as in our previous work [31] and in study 2 (S6 Fig). **(e)** The evidence asymmetry slope (equivalent to a single line fitted across all conditions in panel b) is plotted against the prior-weight *ω*_1_, showing a negative correlation. This correlation closely follows model predictions indicated by the black line (as in Fig 2D but with shaded regions including variability in likelihood-weight *ω*_2_ parameters between the 25^th^ and 75^th^ percentile range of observed values in our previous work [31]). Marginal violin plots show group medians and interquartile ranges. (**f)** The mean final estimate difference is shown against *ω*_1_, again showing a correlation that follows the model prediction (black line as in Fig 2E). Marginal violin plots show group medians and interquartile ranges. **(e, f)** Asterisks indicate a significant sign-rank tests of group medians against the corresponding reference values indicated by the dashed lines. Note that results in (e) and (f) were robust to the exclusion of outliers with an *ω*_1_ more than 3 scaled median absolute deviations [52] from the median (*ω*_1_<0.75; 11 outliers): after their removal, the correlation between *ω*_1_ and the evidence asymmetry slope was still significant (ρ = -0.58, p < 10^−307^), as was the correlation between *ω*_1_ and the mean final estimate difference (ρ = -0.53, p = 2.32 x 10^−12^). Posterior predictive checks further recapitulate the range of values in the data (S10–S12 Figs).

We first checked whether the draw-by-draw probability estimates for the hidden box reported by participants indicated that they generally engaged in the task as we expected. Indeed, averaging across all sequences and participants, probability estimates showed a gradual increase towards higher probabilities for the true hidden box as the number of observed beads increased, and the rate of this increase was higher for bead-ratio conditions denoting stronger evidence ([90:10]>[60:40]>[51:49]; interaction of bead draw [0–8] by bead-ratio condition: t_160.66_ = 26.15, p = 8.82x10^-60^, linear mixed-effects model; Fig 3A and S4 Table), suggesting that participants’ beliefs tracked the cumulative evidence strength of observed samples over a trial. This effect was also obvious in most individual participants and in an analysis restricted to a subset of 16 identical sequences (see Methods) across 60:40 and 90:10 bead-ratio conditions ([90:10] > [60:40]; interaction of bead draw [0–8] by bead-ratio condition: t_182.96_ = 11.81, p = 2.79x10^-24^, linear mixed-effects model; Fig 3A inset and S5 Table). The first estimates before seeing any bead were generally unbiased S3 Fig and no systematic between-trial effects were apparent.

#### Behavioral signatures of sequential base-rate neglect

As mentioned above, the weighted Bayesian model predicts that base-rate neglecters (*ω*_1_ < 1) should have a recency bias. In the sequential context relevant here, the recency bias should manifest at the end of a sequence as higher final probability estimates for the true hidden box when more majority beads (beads whose color is consistent with the true identity of the hidden box) are presented towards the end versus the beginning of the sequence (“back-loaded” versus “front-loaded” sequences, respectively). This would directly show that more recent samples, closer to the end of the sequence, have a stronger influence on the final estimate relative to older samples closer to the beginning (Fig 2A). Furthermore, model simulations showed that this effect should be more apparent when comparing pairs of sequences with more extreme front-loading and back-loading (Fig 2B), which we quantified based on a linear weighted sum of majority beads in the sequence based on their order (1^st^ to 8^th^ position) and which we refer to as ‘evidence asymmetry’ (with respect to the middle of the sequence). In contrast to a base-rate neglecter (prior weight *ω*_1_ < 1), the Bayesian ideal observer exhibits path- or evidence-order-independence in its final beliefs, as do observers with different likelihood weighting (*ω*_2_ ≠ 1; S2 Fig).

Our task design included a systematic sequence-level manipulation of evidence order and asymmetry (Fig 1B) to allow for a direct demonstration of recency bias. Per the above explanation, a simple test for this bias consisted of comparing the final probability estimate between mirror-opposite sequence pairs that had the same number of majority beads and bead ratio but in which majority beads were either front-loaded or back-loaded (Fig 2A). Critically, the data showed evidence-order-dependence in the form of a recency bias consistent with sequential base-rate neglect: pair-wise differences in final probability estimates were higher for back-loaded versus front-loaded sequences (mean final estimate difference > 0, p = 1.66x10^-8^; sign-rank test) and this positive difference increased with evidence asymmetry (evidence asymmetry slope > 0, p = 2.22x10^-11^; sign-rank test), with steeper slopes for stronger evidence ([90:10]>[60:40]>[51:49] bead-ratio conditions; bead ratio x evidence asymmetry interaction: t_151.46_ = 3.107, p = 0.002, linear mixed-effects model; Fig 3B and S6 Table). All three findings conformed with the predictions of the sequential base-rate-neglect model.

A further prediction of the weighted Bayesian model is that sequential base-rate neglect induces a form of prior-dependent belief updating whereby, as the prior increases in favor of one option, the magnitude of logit belief updates to prior-consistent evidence decreases, and it increases to prior-inconsistent evidence (Fig 2C). This impedes reaching full certainty in beliefs over the long run, resulting in more “moderate” beliefs [1,2,17]. In contrast, the ideal observer would predict belief updates of constant magnitude in logit space. In line with sequential base-rate neglect and our model predictions, we observed that mean logit belief updates in response to prior-consistent evidence tended to decrease as prior certainty increased (logit-prior main effect: t_150.30_ = -2.643, p = 0.009; Fig 3C), an effect which was independent of the bead-ratio condition (logit-prior x bead-ratio interaction: t_143.13_ = 0.903, p = 0.368; linear mixed-effects model; S7 Table).

Thus, these model-agnostic results show evidence-order dependence and prior-dependent updating that are generally consistent with the predictions of sequential base-rate neglect under the weighted Bayesian model [1,2,17,31] and are satisfactorily captured by this model based on posterior predictive checks (shaded regions in Fig 3A–3C).

#### Relationship between model-agnostic and model-based measures of sequential base-rate neglect

We carried out a group-level model comparison of variants of Bayesian-inference models, including the (unweighted) Bayesian ideal-observer model, as in previous work [31] (see Methods). As in this previous work, the winning model (Fig 3D; S8 Table) was the weighted Bayesian model with a prior-weight parameter (*ω*_1_) and one likelihood-weight parameter per condition ($\omega_{2_{\left(l\right)}}$, where (*l*) is one of the three bead-ratio conditions). Examining the fitted prior-weight *ω*_1_ parameter values across participants revealed substantial interindividual variability and a general tendency for underweighting of prior beliefs (*ω*_1_<1: p = 2.27x10^-4^, sign-rank test), consistent with sequential base-rate neglect. Critically, and consistent with the model predictions (Fig 2D and 2E), participants exhibiting lower *ω*_1_ values tended to exhibit stronger recency biases and stronger modulation with increasing evidence asymmetry in their final probability estimates (mean final estimate difference: ρ = −0.60, p<2.22 x 10^−308^; evidence asymmetry slope: ρ = −0.64, p<2.22 x 10^−308^; Spearman correlation; Fig 3E and 3F). The evidence asymmetry slope and the mean final estimate difference also correlated strongly with each other (ρ = −0.63, p<2.22 x 10^−308^). Note that the model-predicted relationship between the prior weight *ω*_1_ and these model-agnostic measures of recency bias (Fig 2D and 2E) is non-monotonic for very low values of *ω*_1_ but monotonic for the range of *ω*_1_ values roughly over 0.75, where the majority of our data are (92.72%); results held when analyses were restricted to this monotonic range (see Fig 3 caption). Furthermore, prior-dependent updating—the slope of the logit belief update in the direction of the evidence as a function of the logit prior—across all bead-ratio conditions positively correlated with *ω*_1_ (ρ = 0.71, p<2.22 x 10^−308^), and negatively correlated with the evidence-asymmetry effect (ρ = −0.50, p = 1.32 x 10^−10^) and the mean final estimate difference (ρ = −0.49, p = 2.57 x 10^−10^). These model-predicted relationships all held when controlling for the three $\omega_{2_{\left(l\right)}}$ parameters, and the model root-mean-squared error (RMSE; S9 Table) and were robust to exclusion of potential outliers (see Fig 3 caption).

This indicates consistency across model-based and model-agnostic analyses and highlights the specificity of the relationship between *ω*_1_ and the predicted behavioral signatures of sequential base-rate neglect. Furthermore, measures of general cognition [47] and psychopathology [48] did not show a specific relationship with *ω*_1_, suggesting that variability in *ω*_1_ is unlikely to reflect domain-general factors (despite sufficient variability in both; S4 Fig and S10–13 Tables).

#### Relationship between laboratory indices of sequential base-rate neglect and odd real-world beliefs

Individuals with more extreme sequential base-rate neglect may tend to hold peculiar beliefs due to excessive susceptibility to new evidence (i.e., recency bias) combined with an inability to resolve belief uncertainty [2] (per prior-dependent belief updating). To examine the relevance of interindividual variability in the task-based measures of sequential base-rate neglect to real-world beliefs, we collected a self-report questionnaire that measures proclivity to various odd or unusual beliefs (Peters Delusions Inventory [PDI] [49]; Methods). We did not observe significant relationships between the relevant measures of sequential base-rate neglect and PDI scores (S9 Table). However, very few participants had high PDI scores based on previously published cutoffs [50,51] (only 1–15 participants or ~1–10% of the sample), thus limiting our power to detect relationships with PDI. To address this, we conducted a second study that used pre-screening to ensure an adequate range of PDI scores.

### Study 2

#### Pre-screening, exclusions, and retained sample

**Fig 4:**
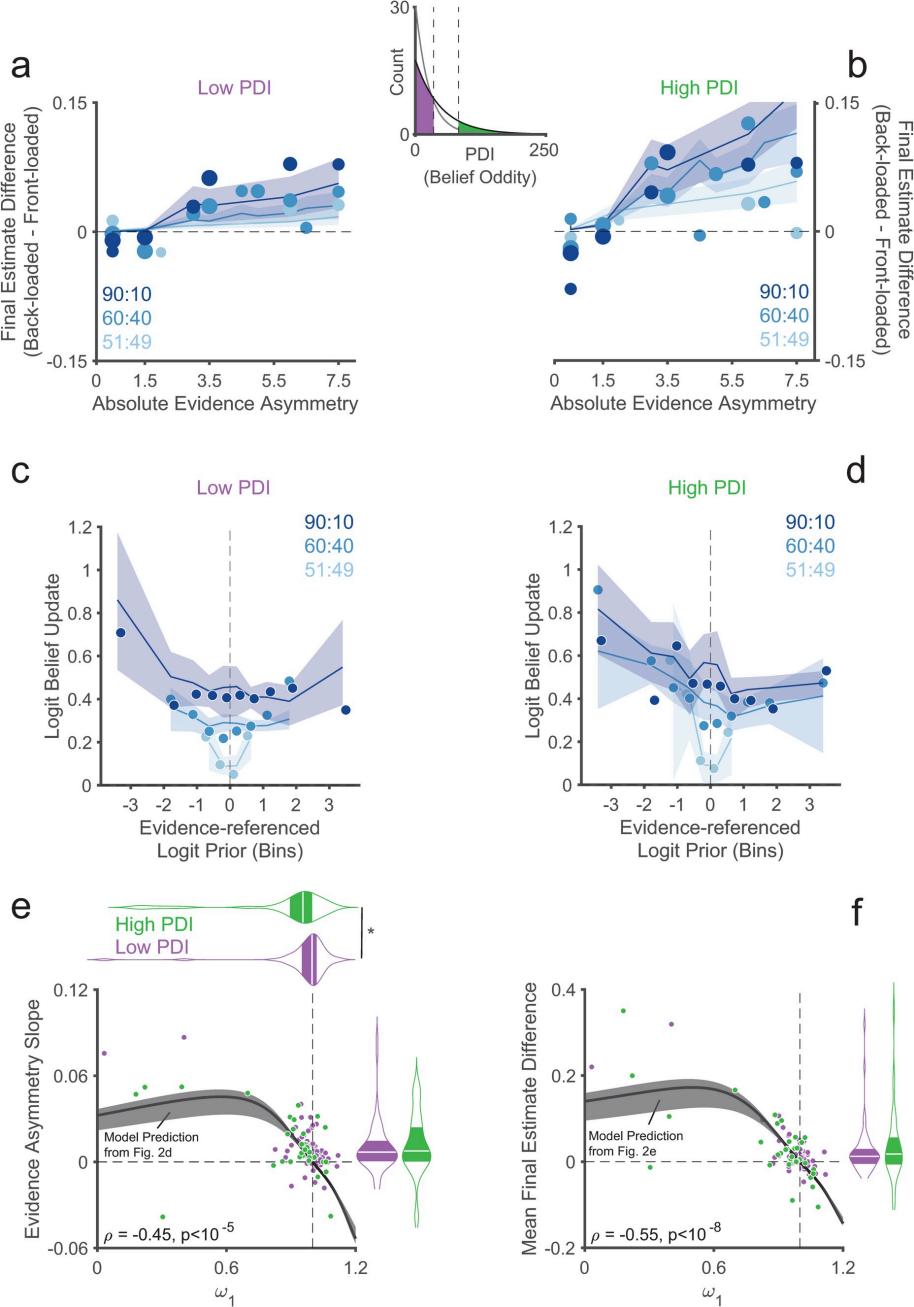
Replication in study 2 of results from study 1. **(a, b)** Mean final estimate difference as a function of evidence asymmetry for the low and high PDI groups independently (S18 and S19 Tables). Solid lines and shaded regions reflect the mean and SEM of the weighted Bayesian model fits. The center inset shows the exponential fit of the distribution of PDI global scores from study 1 (grey line) and study 2 (black line), indicating the cutoffs for high and low PDI by vertical dashed lines. **(c, d)** Logit-belief updates as a function of logit prior by bead ratio for the low (c; S20 Table) and high (d; S21 Table) PDI groups. Group medians of individual medians for logit-belief updates are shown and other conventions follow Fig 3C. Solid lines and shaded regions reflect medians and 95% bootstrapped confidence intervals of the weighted Bayesian model fits. **(e)** Evidence asymmetry slopes are plotted against *ω*_1_ by group. Other conventions as in Fig 3E. Marginal violin plots show the group medians and interquartile ranges. The asterisk indicates a significant rank-sum test comparing group medians of *ω*_1_. **(f)** Mean final estimate differences are plotted against *ω*_1_. The marginal violin plot shows the group medians and interquartile ranges. **(e, f)** The solid black line shows model predictions as in Fig 2E and 2F. As in Fig 3, after excluding outliers [52] (*ω*_1_<0.82; 11 outliers), the correlation between *ω*_1_ and the evidence asymmetry slope was still significant (ρ = -0.37, p = 1.08 x 10^−4^), as was the correlation between *ω*_1_ and the mean final estimate difference (ρ = -0.47, p = 7.32 x 10^−7^).

To ensure a wide range of PDI scores and sufficient high PDI participants with odd beliefs, Study 2 used a pre-screening procedure following prior work [53–56] (Methods). The study consisted of two parts: (i) a pre-screening based on the PDI (and, secondarily, on the Paranoia Checklist; Methods), and (ii) a separate experimental session involving administration of a second PDI and the task discussed above (separated on average by 3.5 days). Critically, the pre-screening used unbiased PDI-score cutoffs derived from previously published norms [49] (under 34.9 for low PDI and over 82.9 for high PDI; Methods). After exclusions (Methods), 116 participants were retained of whom 91 comprised the main sample: 34 in the high PDI group and 57 in the low PDI group (S1 Table; Fig 4A and 4B inset). Attesting to the effectiveness of (and need for) the pre-screening, note that only 2 participants from study 1 would have been classified as high PDI based on study 2’s pre-screening cutoffs.

#### Direct replication of results from study 1

As further validation of our task and model, we replicated the critical results from study 1 in the main sample of study 2 (Fig 4 and S2 Text), including the winning model (S6 Fig; S8 Table).

#### Group differences in sequential base-rate neglect reflect variability in real-world odd beliefs

**Fig 5:**
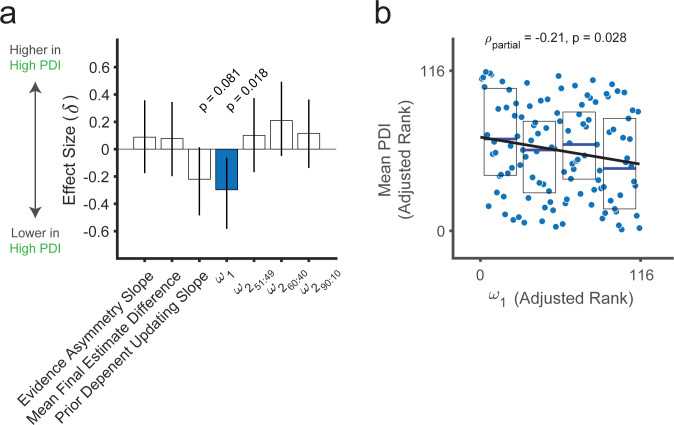
Prior underweighting relates to belief oddity outside the laboratory. **(a)** Summary of group-level differences (S22 Table) for model-based and model-agnostic measures between the low (n = 57) and high (n = 34) PDI groups. Bar plots are effect size (Cliff’s delta, δ) and 95% confidence intervals. **(b)** Scatterplot showing a negative correlation between ranked mean PDI scores and *ω*_1_ (n = 116). Values are adjusted by $\omega_{2_{\left(l\right)}}$ parameter values and the model fit (RMSE) for specificity as in the partial Spearman correlation analysis. Boxplots show medians (blue lines) and 25^th^ and 75^th^ percentiles (bottom and top edges, respectively). The solid black line reflects the least-squares linear fit to the data points. Mean PDI is the average of the global PDI scores across the pre-screening and the experimental sessions for each participant.

Having ensured enough variability in odd beliefs (i.e., PDI scores), we tested whether the high and low PDI groups differed on the relevant measures of sequential base-rate neglect. Both groups separately showed recency biases (mean final estimate difference and evidence asymmetry slope; all p<0.009), but only the high PDI group showed the prior-dependent belief-updating effect (low PDI: p = 0.41; high PDI: p = 0.02; sign-rank tests). There were no group differences for the recency bias measures (mean final estimate difference or evidence asymmetry slope; all p>0.48) or for the prior-dependent belief-updating effect, although the latter trended towards significance (p = 0.083, rank-sum test; Fig 5A). Crucially, the model-based measure of sequential base-rate neglect did differ between the groups, with more sequential base-rate neglect (lower *ω*_1_) in the high PDI compared to the low PDI group (p = 0.018; rank-sum test; effect-size Cliff’s delta δ = 0.30). No group differences were observed in the other model parameters ($\omega_{2_{\left(l\right)}}$: all 0.43>p>0.09; rank-sum tests; -0.10>δ>-0.21; Fig 5A and S22 Table).

Consistent with the observed group differences, an exploratory dimensional analysis (including 25 participants with PDI scores in an intermediate range between the high and low cutoffs in addition to the 91 comprising our primary groups per the pre-screening protocol; n = 116) showed that individuals with more unusual beliefs tended to exhibit lower *ω*_1_ (ρ = −0.25, p = 0.007) and a trend towards stronger prior-dependent belief-updating (i.e., a more negative slope; ρ = −0.17, p = 0.065). The relationship with *ω*_1_ held after controlling for all three $\omega_{2_{\left(l\right)}}$ parameters and the model RMSE (ρ = −0.22, p = 0.021; Fig 5C and S23 Table). The relationship with *ω*_1_ also held after controlling for demographic variables including age, biological sex, race, education, handedness, smoking and drug use status, and previous hospitalizations for psychiatric and neurological conditions (*ρ*_*partial*_ = −0.24, *p* = 0.021), and none of these variables related to PDI scores. Our secondary measure of odd beliefs, the Paranoia Checklist, also correlated with *ω*_1_ (ρ = −0.23, p = 0.014) and the prior-dependent belief-updating (ρ = −0.12, p = 0.039). Overall, the results of study 2 suggest that a laboratory measure of sequential base-rate neglect relates specifically to odd beliefs outside the laboratory.

### Study 3

#### Functional explanations for sequential base-rate neglect

Thus far, we have shown evidence that human behavior in a sequential belief-updating task generally conforms to the predictions of a weighted Bayesian model of sequential base-rate neglect. Specifically, this model jointly predicts a recency bias and a pattern of prior-dependent updating as well as interindividual relationships with prior underweighting that we observed empirically. Further, interindividual variability in sequential base-rate neglect correlates with real-world belief oddity. However, this descriptive model does not provide a normative explanation as to *why* sequential base-rate neglect is such a predominant feature or why it varies across individuals. It also does not address whether prior underweighting may or may not be an optimal strategy under realistic constraints.

**Fig 6:**
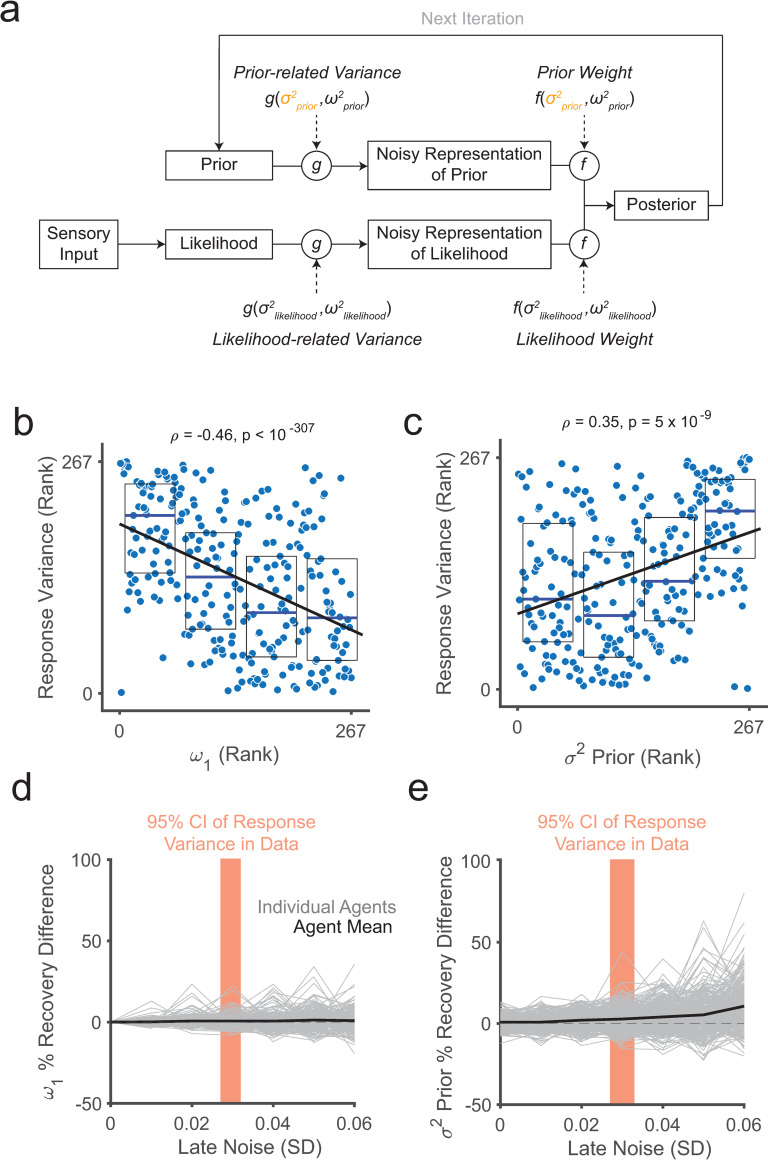
Relationship between prior underweighting, prior noise, and response variance. (a) Visual Schematic of Noisy Sampling Model. The noisy-sampling model captures an iterative sequential belief updating process where the internal representation of prior (and likelihood information) is noisy (see Methods). This is based on an agents’ uncertainty about the true values of the prior and likelihood, given recent evidence, with variances $\sigma_{prior}^{2}$ and $\sigma_{likelihood}^{2}$, and their assumed distributions of priors and likelihoods, with variances $\omega_{prior}^{2}$ and $\omega_{likelihood}^{2}$. Note that variables are in logit space and noise consists of an additive zero-mean Gaussian distribution. Critical to the model are a noisy representation of the prior and likelihood where the noise is given by the functions $g(\sigma_{prior}^{2},\omega_{prior}^{2})$ and $g(\sigma_{likelihood}^{2},\omega_{likeliood}^{2})$. More noise (e.g., due to higher $\sigma_{prior}^{2}$) leads to more random variability in responses reflecting the posterior belief (even for repetitions of identical sequence fragments, as captured by the model-agnostic measure of response variance). Optimal inference results from adjusting weighting commensurate with the degree of noise, with optimal weights given by the functions $f(\sigma_{prior}^{2},\omega_{prior}^{2})$ and $f(\sigma_{likelihood}^{2},\omega_{likeliood}^{2})$. Finally, the optimal posterior is a weighted sum of the noisy prior and noisy likelihood in logit space. Model fitting used 4 free parameters, 1 shared $\sigma_{prior}^{2}$ parameter and condition-specific $\sigma_{likelihood}^{2}$ parameters (3), and a grid search with 4 fixed parameters for $\omega_{prior}^{2}$ (1) and $\omega_{likelihood}^{2}$ (3) (Methods). **(b)** Scatterplot of ranked response variance rank and *ω*_1_ showing a negative relationship indicating that individuals with more sequential base-rate neglect have more variability in their probability estimates for identical sequence fragments (Methods). **(c)** Scatterplot of ranked response variance and prior noise $\sigma_{prior}^{2}$ showing a positive relationship indicating that the model-agnostic measure of response variability scales with the model-derived measure of prior noise. **(b, c)** Boxplots reflect median (blue) and 25^th^ and 75^th^ percentiles (bottom and top edges, respectively). Black lines show the least-squares linear fit of the data points. **(e, f)** Noise-corrupted parameter recovery analysis for the weighted Bayesian model (e) and the noisy-sampling model (f). The y-axis shows the percent deviation in the recovered versus the original parameter values. The x-axis shows the magnitude of the late Gaussian noise added at the response level in the model simulations in standard deviation. Each grey line depicts a single agent defined by a set of parameter values across a range of noise levels. The red shaded area indicates the estimated range of response variance found in the actual data as a 95% confidence interval based on the median response variance (see Methods). On average (black line), the critical parameters are adequately recovered, without systematic biases in their estimation for meaningful levels of late Gaussian noise (particularly for the weighted Bayesian model), indicating that low-level factors such as general inattention or random responding are unlikely to explain variability in *ω*_1_ values.

Study 3 thus aimed to address these outstanding mechanistic questions of why people exhibit base-rate neglect and whether it could reflect an optimal strategy. To do so, we considered models that explain variation in prior weighting as a rational response to external or internal factors. We specifically considered a first class of functional models that explains sequential base-rate neglect and its variability as a consequence of perceived variability in the environment [45,46] and a second class that explains it as a rational adjustment to a noisy internal sampling process [16]. In the first, in a volatile environment where the underlying evidence-generating process can change abruptly, the relevance of evidence before an inferred change point should be proportional to the certainty that a change point occurred. Thus, an optimal agent that perceives the environment as volatile will tend to decrease the prior weight around potential change points, therefore exhibiting sequential base-rate neglect. This class of model should be less applicable to the stable environment in the current task, but we reasoned that participants could still assume some degree of volatility despite explicit instructions to the contrary. The second class of models prescribes the optimal behavior for an agent with limited cognitive resources [57]. Under the noisy-sampling model [16] within this class, the agent can only access an imprecise, noisy representation of prior beliefs through random sampling of its internal representation (i.e., the distribution of the logit prior resulting from additive noise); it is possible to increase precision of the prior representation by increasing samples at the cost of allocating more internal cognitive resources, but the optimal strategy balances this cost against that of prediction inaccuracy (Fig 6A). Given this, the optimal strategy in this capacity-limited agent consists of decreasing the prior weight more in response to greater noise in the prior representation [16]. Because more noise in the prior representation should lead to more variable responses, even after accounting for structured variability due to sequential effects the noisy-sampling model predicts a correlation between the degree of sequential base-rate neglect and (unstructured) response variance. In contrast, the alternative volatility-based model we considered here does not predict this correlation in the context of our task (S7 Fig).

We thus assessed the interindividual correlation between prior-weight *ω*_1_ and response variance across all 267 participants from studies 1 and 2. A clear correlation was observed with *ω*_1_ when using the unexplained variance by the weighted Bayesian model (the model RMSE) as an index of unstructured response variability (ρ = −0.40, p = 8.9 x 10^−12^, Spearman correlation; Fig 6A). This relationship was also present in each independent sample (S24 Table). To circumvent potential artifacts of modeling, we also derived a model-agnostic measure of response variance focused on the unstructured variability of responses under identical circumstances—specifically, the aggregate response variance of logit probability estimates for repeated, identical sequence fragments matched on bead color and bead ratio (Methods), which we refer to as the response variance for simplicity and which captures variability that cannot be attributed to sequential evidence-order effects. Using this measure, we again found a correlation with *ω*_1_ in the expected direction (ρ = −0.46, p<2.22 x 10^−308^; Fig 6B). Again, this relationship was also present in each independent sample (S24 Table). Although this result does not rule out the broader class of volatility models, it is more consistent with the noisy-sampling model; we thus further explored the ability of the latter model to capture our data.

#### The noisy-sampling model captures belief-updating behaviors described by the weighted Bayesian model

The noisy-sampling model posits that the prior and likelihood weights of the weighted Bayesian model (*ω*_1_ and *ω*_2(*l*)_) scale negatively with the respective noise in the representation of the prior and likelihood, captured respectively by parameters $\sigma_{prior}^{2}$ and $\sigma_{likelihood\;(l)}^{2}$ (S25 Table). The noisy-sampling model also includes parameters $\omega_{prior}^{2}$ and $\omega_{likelihood\;(l)}^{2}$ that reflect the uncertainty in the distribution of logit priors and logit likelihoods that the agent might encounter (here held constant for model fitting to avoid parameter tradeoff; Methods). Perhaps unsurprisingly given that the structure of the noisy-sampling model reduces to the weighted Bayesian model, when fitted to our data (Methods) the noisy-sampling model captured comparable variance (correlation of explained R^2^ between models: ρ = 0.93) and the *σ*^2^ noise parameters closely correlated with the corresponding weights of the weighted Bayesian model (mean Spearman correlation ρ = −0.92; Fig 6C and S8 Fig) in the full sample combining studies 1 and 2 (n = 267). Under the noisy-sampling model, behavioral variability is partly due to noise in the internal representation of variables such as the prior. If this is true and it explains the observed correlation between *ω*_1_ and response variance, prior noise should correlate with response variance. Consistent with this, the fitted parameter $\sigma_{prior}^{2}$ correlated with response variance (ρ = 0.35, p = 5.06 x 10^−9^; Fig 6D and S24 Table). Control analyses evaluating contributions of $\omega_{prior}^{2}$ suggested that this parameter had no meaningful contribution to response variance or base-rate neglect (S9 Fig and Methods).

#### Alternative explanations to noisy sampling

A possible alternative explanation of the observed correlation between prior weight *ω*_1_ and response variance may be that individuals respond more inconsistently not because of noisy internal representations but due to other lower-level factors such as distraction or late motor noise. In other words, some inattentive participants could in principle tend to respond randomly. Although this is unlikely based on control analyses (S8 Fig and S9 Fig), if this were the case, perhaps data from these individuals was better fitted with lower *ω*_1_ values due to modeling artifacts. To evaluate this possibility, we assessed robustness of parameter recovery for the weighted Bayesian model and the noisy-sampling model in the presence of levels of late noise that could capture random responding during the task (Methods). These analyses showed that parameter recovery of the relevant parameters (*ω*_1_ and $\sigma_{prior}^{2}$) had no appreciable biases at levels of late noise matching the observed behavioral variability in the data (Fig 6E and 6F), suggesting that variability in their fitted values is unlikely to stem from lower-level factors irrelevant to the noisy-sampling model. Moreover, an explanation of prior underweighting in terms of inattentiveness may predict modulations of response times by *ω*_1_ that were not present in the data (S27 Table). Altogether, these results speak against an explanation in terms of inattention or random responding and support noisy representation of prior beliefs as a more tenable explanation for sequential base-rate neglect.

#### Relationship between prior noise and real-world odd beliefs

**Fig 7:**
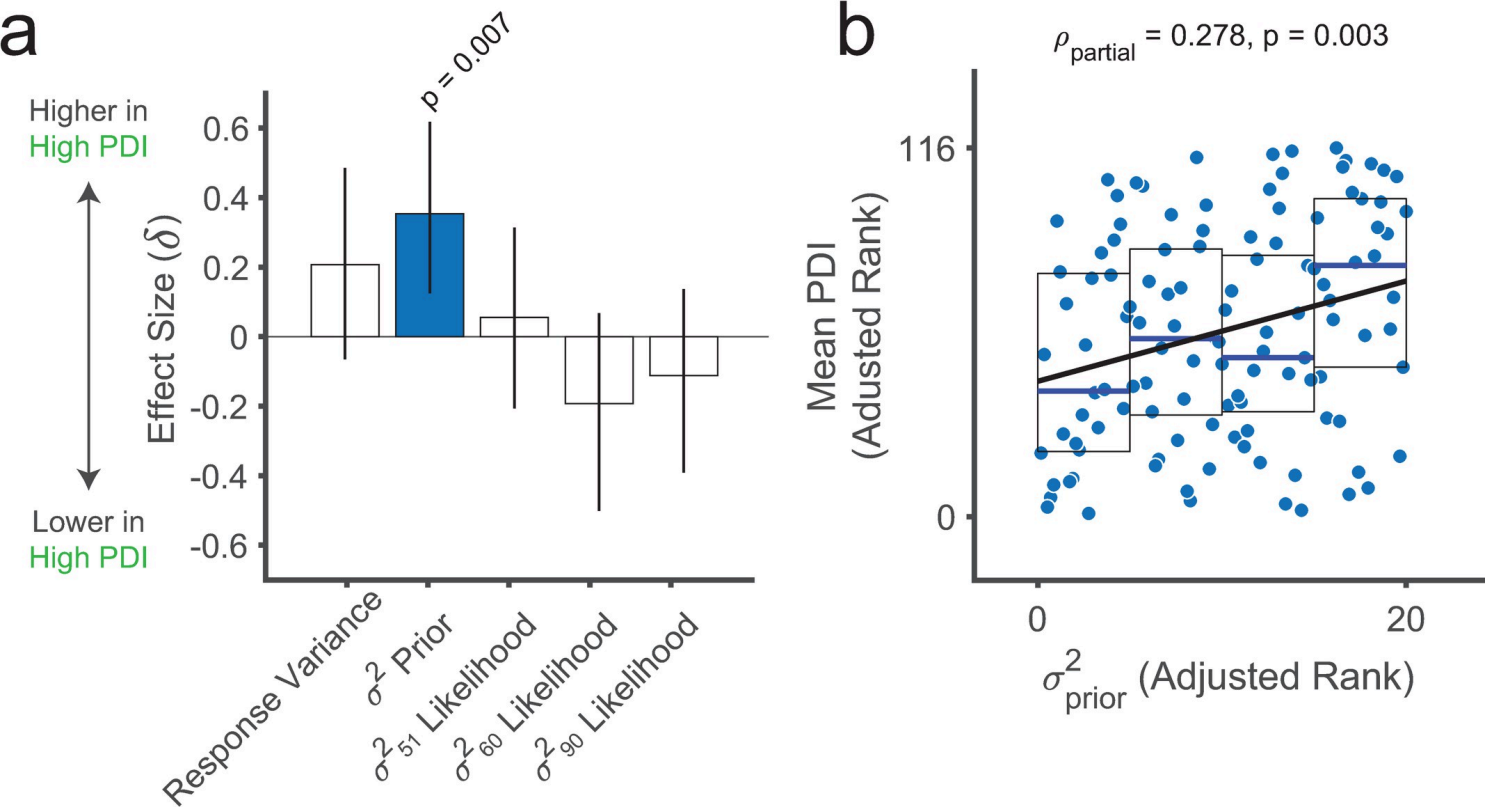
Prior noise relates to belief oddity outside the laboratory. **(a)** Summary of group-level differences for noisy-sampling model-based and model-agnostic measures between the low (n = 57) and high (n = 34) PDI groups. Bar plots are effect size (Cliff’s delta, δ) and 95% confidence intervals. **(b)** Scatterplot showing a positive correlation between ranked mean PDI scores and $\sigma_{prior}^{2}$ (n = 116). Values are adjusted by $\omega_{2_{\left(l\right)}}$ parameter values and the model fit (RMSE) for specificity as in the partial Spearman correlation analysis. Boxplots show medians (blue lines) and 25^th^ and 75^th^ percentiles (bottom and top edges, respectively). The solid black line reflects the least-squares linear fit to the data points. Mean PDI is the average of the global PDI scores across the pre-screening and the experimental sessions for each participant.

Because belief oddity correlated with sequential base-rate neglect (lower *ω*_1_) in study 2, and the previous results imply that prior noise ($\sigma_{prior}^{2}$) could explain sequential base-rate neglect, we next asked whether prior noise could account for belief oddity. In the main sample from study 2, the high PDI group showed higher $\sigma_{prior}^{2}$ than the low PDI group (p = 0.007, rank-sum test; δ = -0.34; Fig 7A). No group differences were observed in the other model parameters ($\sigma_{likelihood\;(l)}^{2}$: all 0.40>p>0.12, rank sum tests; 0.18>δ>-0.53) or in response variance (p = 0.12, rank-sum test; δ = -0.20; S26 Table). These results suggest that high PDI may be specifically associated with increased prior noise.

An exploratory dimensional analysis (using the same sample as in Fig 5B and 5C) further showed a correlation between prior noise $\sigma_{prior}^{2}$ and more unusual beliefs (ρ = 0.29, p = 0.002; Fig 7B), even after controlling for all three $\sigma_{likelihood\;(l)}^{2}$ parameters and the noisy sampling model RMSE (ρ = 0.265, p = 0.0048; S24 Table). Altogether, these results suggest that noisy prior representations may explain sequential base-rate neglect and interindividual variability in odd beliefs outside the laboratory.

## Discussion

In this study, we leveraged computational modeling and a novel task developed to test the joint predictions of a weighted Bayesian model of sequential base-rate neglect. People tended to exhibit base-rate neglect—defined as prior underweighting based on long-standing [5] and recent theories [1,2]—which in a sequential context manifested in two ways consistent with model predictions [1,2,17]: a recency bias, apparent in the raw differences in final probability estimates between mirror-opposite sequences, and a form of prior-dependent updating, apparent in the changes of probability estimates. Crucially, interindividual variability in the magnitude of these effects was commensurate with the degree of sequential base-rate neglect exhibited by individuals. We also considered functional explanatory models of base-rate neglect, and found initial support for a noisy-sampling model[16] whereby sequential base-rate neglect represents an optimal response to noisy representation of prior beliefs—in contrast to classical theories that frame base-rate neglect as a suboptimal heuristic strategy [3,5]. The noisy-sampling model predicted correlations between sequential base-rate neglect and unstructured response variance that we corroborated in the data. Finally, both model-derived measures of sequential base-rate neglect and prior noise from our laboratory task correlated with the endorsement of odd beliefs outside the laboratory, suggesting the relevance of these computationally characterized processes to the development and maintenance of real-world beliefs.

Our study goes beyond previous studies showing evidence-order effects in sequential belief updating [19–31,33,34] in several ways. First, our study used explicit quantitative information from a single evidence stream as the basis for both prior beliefs and the likelihood of evidence samples. This allowed us to rule out meaningful baseline biases (S3 Fig) as each of two alternative states was confirmed to be considered equally likely before any evidence was presented. It also equalized the relevance and representativeness of the prior and likelihood information, making interpretations of base-rate neglect in terms of qualitative differences between observed evidence and base-rate information [5,40] less tenable. Second, we used a validated belief-elicitation procedure that financially incentivized participants to report their true beliefs [35,36,38]. Third, we used computational modeling to parse the role of prior weighting during sequential belief updating. Combined with longer sequences and a novel manipulation of evidence order, this allowed us to systematically characterize evidence-order effects and empirically confirm the theoretical prediction [1,2,4,32] that sequential base-rate neglect expresses itself as a combination of recency bias and prior-dependent belief updating imposing a ceiling on belief certainty. And fourth, past studies have found an association between cognitive biases and odd beliefs [58–63] in the general population. However, their primary findings center on correlations between odd beliefs and broadly defined or composite measures of reasoning or cognitive biases—rather than more narrowly defined and more interpretable cognitive constructs defined via computational modeling. These broader measures have yielded mixed results, possibly due to the qualitative nature of the reasoning tasks or other limitations such as a small number of trials. In contrast, the current study identified a specific relationship between a precisely defined computational measure of sequential base-rate neglect from a well-controlled paradigm and a subjective report of odd beliefs in the general population.

A unifying theory for *why* people exhibit sequential base-rate neglect has been lacking [41]. A classic influential view of base-rate neglect framed it as a heuristic strategy [5,40–44], although this notion lacked clear support and a fully developed explanatory framework. Here, we provide empirical support for an alternative functional (mechanistic) model [16] that explains sequential base-rate neglect as an efficient response to noise in the internal representation of prior beliefs. Individuals are assumed to have a certain trait-like degree of prior noise, or imprecision, and they can adapt to it by modulating its influence or weight on belief updating. Given limited internal resources (e.g., cognitive or metabolic), individuals must balance the internal costs of precision against the cost of incorrect predictions [64–66]. And given the limited precision with which prior information can be represented, the optimal strategy is to discount prior information (i.e., to neglect the base rate) in proportion to the degree of prior imprecision. We also considered alternative explanations to the noisy-sampling model, including lower-level factors such as inattention, ultimately deeming an explanation in terms of a response to internal prior noise to be more tenable. Partly supporting this conclusion, we found empirical support for a key prediction of the noisy-sampling model that the degree of prior noise should relate to the amount of unstructured variability reflected in response variance (beyond structured variability related to evidence-order effects). This conclusion is also in line with the finding that the degree of base-rate neglect depends on the perceived trustworthiness of prior information [67]. Our results may also be reconciled with the observation that recency bias is more prevalent upon sequential belief elicitation (as in our paradigm) versus end-of-sequence single-shot belief elicitation [24], at least if we assume that belief updates only occur upon each elicitation [1,2], since more elicitations should lead to more prior discounting with each belief update. Generally speaking, our results thus align with an emergent literature supporting the relevance and biological plausibility of sampling-based inference models [68–77]. While here we focused on a specific model of inference under internal capacity constraints, our main results are broadly consistent with this model family—including a learning-to-infer model where prior underweighting and sequential biases arise through learning of contextual information [78]—and thus support further examination of these models in future work.

Finally, we extended previous field work linking sequential base-rate neglect to real-world judgments by demonstrating that individuals with more sequential base-rate neglect and noisier prior beliefs tend to endorse more odd beliefs in their daily lives [79]—beliefs that are likely to influence how they function in society [53,80–86]. Notably, this has implications for psychiatric disorders involving delusions [17,31] or odd unsupported beliefs. Previous literature has emphasized a “jumping to conclusions” [87–92] bias in schizophrenia, although interpretations of this bias in terms of altered belief updating in relation to delusions remains questionable [17,31,93–96]. Using a similar approach to the current paper, we previously showed that variability in sequential prior weighting correlated with the clinical delusion severity in schizophrenia [31], suggesting a role for sequential base-rate neglect in belief psychopathology. Our finding that sequential base-rate neglect drives evidence order effects implies that different sequences of information may lead to inconsistent differences in certainty (and by extension information sampling) in schizophrenia [17], which could explain mixed results in this literature [93–95]. Systematic manipulations of evidence order such as the ones we introduced here may thus be helpful in clarifying the computational mechanisms underlying delusions. Further, our results also emphasize that alterations in noisy-sampling (and other limited-capacity) inference processes should be evaluated as candidate explanations for maladaptive or pathological beliefs, particularly given increasing support for their role in adaptive behaviors [97,98].

Our results indicate that sequential base-rate neglect makes human observers rely disproportionately on recent evidence. They also hint at a potential strategy that could be used to avoid or minimize potentially harmful consequences of sequential base-rate neglect. By manipulating the magnitude of evidence asymmetry, we showed that recency biases tend to disappear in sequences with balanced information (i.e., they approach zero as evidence asymmetry approaches zero; e.g., as shown in Fig 3B). This suggests that sequential information curated to maximize evidence *symmetry* may facilitate the development of unbiased beliefs. This principle could apply to real-world situations where unbiased, objective judgments are vital, like a clinician making a diagnosis or a jury rendering a verdict. In the former case, previous work has shown that the order of information affects diagnostic accuracy [8,20,99], so ensuring a balanced sequence of information—e.g., via medical decision-making scripts [100]—could plausibly minimize biases and improve diagnostic accuracy. More generally, our results suggest that symmetrically interleaving opposing pieces of evidence may yield a more balanced synthesis of the information at hand.

In summary, we have showed that base-rate neglect manifests sequentially as a combination of recency bias and prior dependence in belief updating, that this process may result from a noisy representation of prior beliefs, and that it likely contributes to the formation of odd beliefs in the real world. Altogether, our findings suggest that sequential base-rate neglect is not just a mathematical quirk or an artifact of laboratory methods but a robust feature of human belief formation.

## Methods

### Ethics statement

All participants provided written informed consent. This study was approved by the Institutional Review Board at the New York State Psychiatric Institute (Protocol #6916).

### Incentive-compatible probability-estimates beads task

#### Task aim

We developed a modified beads task building from previous work[31] where participants had to infer the identity of a hidden state (blue or green box) based on multiple samples of evidence (colored beads). We elicited probability estimates about the identity of the hidden state after each sample of evidence, allowing us to track the development of beliefs over time. We manipulated both the strength of the evidence (bead ratio in the hidden box) and, critically, the order in which the evidence samples were presented. The same task was used in studies 1, 2, and 3.

#### Trial structure

Trials started with a 3-s presentation of two boxes with the same majority-to-minority bead ratio and different majority bead color (i.e., the blue box and the green box). To enhance clarity, the border of each box indicated the color of the majority bead color in the box and the contents of each box were displayed in text above each box (e.g., “60 blue, 40 green”; Fig 1A). One box was presented on the left side of the screen and the other on the right side of the screen. Next, participants were shown a white box with a black border and a question mark, which represented the hidden box. The first time it was displayed in a trial, participants just saw this hidden box for 1.5 s. On subsequent presentations of the hidden box, an animated green or blue bead rose up out of the box with this animation lasting 1.5 s. Participants then reported a probability estimate about how likely they thought the hidden box was the blue box or the green box. The top half of the screen showed a visual record of all beads shown so far within the trial, so as to minimize the working-memory burden and associated interindividual variability. The lower half of the screen displayed a black slider bar used to submit the probability estimate. Percentage values above each extreme of the slider indicated the complementary probability estimates for each box. The slider tick did not appear until a participant moved the mouse, and its starting point was randomized after each bead draw to minimize anchoring. Probability-estimate responses were self-paced and the response window was unlimited. After 8 samples were drawn and 9 probability estimates submitted, a binary choice for the hidden box was prompted. On this choice screen, the boxes were labeled “Left” and “Right” and participants had to respond with a corresponding (left or right) button press within an unlimited response window. When a response was submitted, the border of the selected box changed to yellow for 0.25 s to provide feedback that the selection was recorded. A blank screen was then presented for 0.5 s before the next trial began.

#### Task structure

Participants completed 55 trials of the probability estimates task. At the beginning the experiment, participants were instructed that one of two boxes was randomly selected and hidden with equal probability: one containing mostly blue beads (blue box) or one containing mostly green beads (green box). One box was presented on the left side of the screen and the other on the right. The location of each box was determined at random on each trial. For a given trial, the bead ratio could be 51:49, 60:40, or 90:10, with each box displaying reciprocal ratios of bead colors. The participants’ task was to identify which box was selected and accurately estimate its probability. During each trial, 8 bead samples were presented, one at a time, and probability estimates were prompted before the first bead and after each of the beads about the probability that the hidden box was the blue or the green one. Participants were told that the individual beads were drawn randomly with replacement. To endow the estimates with instrumental value, after seeing 8 beads participants made a binary choice about the identity of the hidden box.

#### Incentive compatibility

During the instructions, to incentivize responses that accurately reflected true beliefs and preferences, participants were informed they would be given an endowment of $10 that they could keep in its entirety (losing $0 or $5) based on their performance. After they completed all blocks of the experiment, $0 or $5 were subtracted from the endowment based on their performance on one randomly selected response. This could be a probability estimate (out of the 9 per trial over all trials) or a binary box choice (1 per trial). To determine the payoff, we instituted a binarized scoring rule [39] that is more robust to risk preferences and produces more accurate estimates than other commonly used methods [39], particularly when combined with potential loss from an endowment as in our implementation and in previous work leveraging endowment effects [101,102] to maximize task engagement and accuracy. If a probability estimate was chosen to determine the payoff, the probability of losing $5 was a function of the squared error of the reported probability estimate relative to the objective probability [103]. Specifically, a random value k from 0 to 1 was selected and participants lost $5 if the squared error of their chosen estimate was larger than k or $0 otherwise. The binarized scoring rule thus implies a quadratic loss function where the probability of losing $0 or $5, rather than the loss magnitude, depends on the precision of reported probability estimates. This leads to a U-shaped relationship between the expected value of a response and the posterior probability (S13 Fig). We also applied the binarized scoring rule to box choices, which in this case reduced to losing $5 when the choice was incorrect or $0 otherwise.

At the end of the task, participants were shown the selected response and the payout realization as explained above. To make the underlying principle of the scoring rule clear, an accessible explanation without excessive mathematical detail and several examples were presented to participants during the instructions (S1 Movie). To ensure comprehension, four of the miscomprehension quiz questions (S31 Table, Questions 1, 3, 4, and 9) specifically probed participants’ understanding of the scoring rule.

#### Instructions, practice, and comprehension checks

To ensure participants completed the task within a reasonable time frame and in one session, they were required to complete the entire experiment within 4 hours (S14 Fig). The MTurk advertisement indicated that the task could take up to 2 hours to incentivize participants to minimize breaks. To minimize the incentive to rush through the task, participants were required to perform task trials for at least 40 minutes (and received additional trials if they completed the actual experimental trials earlier). To ensure task comprehension, participants were given comprehensive and detailed instructions for ~15–20 minutes. After the instructions, participants were required to complete a miscomprehension quiz (S31 Table). They were required to achieve 100% accuracy on the quiz or retake it until they did, consistent with prior work [48]. After the quiz, participants completed 3 practice trials, one with each possible bead ratio, and could repeat the practice if they wished. The practice-trial sequences were not used in the main experiment. A video demonstrating the instructions, quiz, and practice trials is available (S1 Movie).

#### Sequences of evidence

Bead sequences were defined by the specific order of majority-to-minority beads. The color (blue or green) of the majority beads in the hidden box, which determined its identity, was randomly determined on each trial. Each bead-ratio condition comprised a different set of pre-determined fixed sequences; these were chosen from a broader set of all possible sequences of beads drawn randomly with replacement, in line with the instructions. Out of the 55 trials, there were 26 unique sequences of evidence order (S30 Table). Of those, 16 sequences were identical across the 60:40 and 90:10 conditions (“matched trials”). Sequences were presented in blocks of 11 trials, organized by the bead-ratio condition. The order of blocks was the same for each participant: 60:40, 90:10, 51:49, 60:40, 90:10. Within each block, sequences were selected at random without replacement from the sequence set. We selected sets of sequences for which the distribution of majority beads over trials for a given bead ratio matched the distribution of expected sequences of that ratio. To achieve this, 4 sequences were unique to the 51:49 condition, 6 sequences to the 60:40 condition, and none to the 90:10 condition.

Critically, we constructed mirror-opposite sequence pairs to facilitate isolation of sequence-order effects. Further, we aimed to vary sequences in their degree of evidence asymmetry, or how extremely front- or back-loaded the majority beads were in a sequence. Here, and throughout the manuscript, sequences are presented such that 1 (or black) represents the majority bead color, and 0 (or white) the minority color. We quantified evidence asymmetry as a linear weighted sum of a binary sequence, for instance [1 1 0 1 1 1 1 0], with each element in the sequence vector weighted as a function of their linear distance from the middle (weights: [-3.5, -2.5, -1.5, -0.5, 0.5, 1.5, 2.5, 3.5]). The result of the weighted sum (in this example, -2) thus indicated that majority beads were presented mostly towards the beginning of the sequence (front-loaded) with negative values. Positive values would indicate that majority beads were presented mostly towards the end of the sequence (back-loaded). Greater absolute values indicate more extreme back- or front-loading. Mirror-opposite sequence pairs had identical bead-ratio, number of majority beads, and absolute evidence asymmetry such that their comparison would isolate sequence-order effects. In particular, recency biases should manifest as more certain beliefs (favoring the true hidden box) for back-loaded (compared to front-loaded) sequences, particularly in sequences with greater evidence asymmetry.

Overall, we selected trials to span a range of evidence asymmetry, bead-ratio conditions, and total number of majority beads (Fig 1B).

### Questionnaires

#### Study 1

Before completing the probability estimates beads task, participants completed a demographic survey (S1 Table) and the PDI^22^ (see S29 Table for the complete set of items). The PDI is a 21-item questionnaire that measures odd, delusion-like ideas in the general population. The experiences interrogated a range of more common experiences such as “do you ever feel as if some people are not what they seem to be?” or “are you worried that your partner may be unfaithful?” to more unusual ones, like “do you ever feel as if you are a robot or zombie without a will of your own?” or “do you feel as if things in magazines or on TV were written especially for you?” For each experience, the participant can endorse the belief with a Yes or No response. If they report No, then the global item score is 0. If they report Yes, they must then report on a scale of 1 to 5 how distressing the belief is (1 = not distressing at all, to 5 = very distressing), how often they think about it (1 = hardly ever, to 5 = all the time), and their conviction about it (1 = don’t believe it’s true, to 5 = believe it is absolutely true). The global item score is the sum of these three responses plus the “Yes” endorsement. The global PDI score (with a possible range of 0 to 336) is the sum of all the global item scores (each between 0 and 16).

#### Study 2

Participants completed the PDI and the Paranoia Checklist[104]. Although our primary measure was PDI, we included the Paranoia Checklist for exploratory purposes to assess the generalizability of the results and to confirm that recency bias was generally related to odd beliefs and not to paranoid beliefs specifically. The Paranoia Checklist is a 18-item measure of paranoid beliefs in the general population.

### Participants, Exclusions, and Retained samples

#### Study 1

Participants were recruited through Amazon MTurk, and the experiment was run on gorilla.sc [105]. They were paid $10 plus a performance bonus of $5 or $10. Using MTurk filters, we only invited participants who had already successfully completed at least 50 tasks with a 90% approval rate, who were under 55 years old, located in the US, and had an MTurk Masters Qualification (given to workers who “demonstrate a high degree of success in performing a wide range of [tasks] across a large number of requesters”).

The experiment comprised multiple components, including the task itself and questionnaires. Several participants began the study, completing the questionnaires but not the task. These non-completers were excluded from all analyses. Importantly, at least for those who completed the questionnaires (S1 Table and S2 Table) we did not find differences in belief oddity (p = 0.484) or evidence for selection biases in completers on most relevant measures.

We also implemented exclusion criteria based on performance. First, to avoid “bots”, we assessed if average responses were above a minimum of 350 ms (the approximate time needed to shift endogenous attention [106]). No participants were excluded by this criterion. Second, we limited our analysis to participants who identified the “correct” box at the end of the bead sequence with accuracy above 68% for 60:40 and 90:10 bead-ratio conditions based on the binomial chance level (15 correct trials out of 22; accuracy criterion). Third, to assess engagement in the task, we used a linear regression analysis predicting participants’ subjective probability estimates based on the random starting point of the cursor on the sliding scale (see task details below) and the optimal Bayesian estimate (i.e., the objective probability). Participants were excluded if the random cursor start position significantly predicted their subjective estimates *and* the Bayesian estimate *did not*. If both conditions were satisfied (random-estimates criterion), we reasoned that the participant was likely trying to the task as fast as possible with no regard for accuracy. If the optimal Bayesian estimate also predicted subjective estimates, we reasoned that the participant may have been engaged in the task but was anchoring to the random cursor-start location, which was insufficient for exclusion. A total of 213 participants began this study. 43 were non-completers, and 8 were excluded for meeting either the accuracy or the random-estimates criterion.

To further determine if participants were correctly engaging the task, we developed 2 heuristic models reflecting strategies that participants may have used and which would not reflect belief updating. The first heuristic model (no-prior model; equivalent to *ω*_1_ = 0) reflects a strategy where participants report fixed belief certainty in favor of the most recent evidence sample. For example, for a blue bead they would report 0.8 in favor of the blue box and for a green bead 0.8 in favor of the green box. The second heuristic model (observed-proportion) reflects a similar strategy with the difference that the favored box is based on whichever color has been drawn more often. For instance, after observing 3 blue beads and 1 green bead the participant could report 0.8 in favor of the blue box and only change their estimate to 0.8 in favor of the green box after observing more green than blue beads. The heuristic models, along with all the belief updating models (S28 Table), were fit to the data for the 60:40 and 90:10 conditions. The 51:49 condition was not used here because the low evidence strength in this condition makes it harder to determine whether estimates are consistent with heuristic strategies. We excluded any participant whose 60:40 and 90:10 data was fit best by one of the heuristic models in a formal model comparison using the BIC [107] at the individual level. Based on this criterion, 5 participants were excluded for being best fit by the no-prior model, and 6 participants by the observed-proportion model. There were no significant differences in demographic characteristics between participants who were included in the study and those who were excluded based on performance criteria or non-completers (S2 Table).

In sum, 237 participants were recruited from Amazon MTurk, of whom 170 completed the task. Of these, 8 were excluded for poor accuracy or random responding and 11 because their data was best fit by a heuristic model which suggested they did not engage the task as intended. After exclusions, 151 participants were retained and included in the primary analysis. S1 Table shows the demographic information of all 151 completers who were included in the analysis for study 1. Quality checks indicated the data was of comparable quality than similar in-person studies (S14 Fig and S15 Fig; see *Online Data Quality in Methods*).

#### Study 2

Participants were recruited through Amazon MTurk, and the experiment was run on gorilla.sc [105]. We implemented the same MTurk filtering criteria as in study 1, with the exception that we did not limit participants to MTurk Masters so as to increase participation. We also excluded anyone who already participated in study 1. Study 2 consisted of two parts. For part 1, participants were paid $2 to complete 2 questionnaires. 547 participants started part 1, and 512 completed it (93.6%). Participants were invited back for part 2 based on their questionnaire scores from part 1. 241 participants were invited to participate in part 2. For part 2, participants completed 1 questionnaire and the probability estimates beads task. The task and incentive structure was identical to study 1. 213 participants started part 2, and 143 participants completed it (67.14%). We applied the same performance and model-based exclusion criteria as in study 1. Using these criteria, 10 participants were excluded based on their performance (accuracy and random-estimates criteria) and 17 due to evidence (BIC) favoring heuristic models. There were no significant differences in demographic characteristics between participants included in the study and those excluded based on poor performance (S3 Table). For study 2, we analyzed the data for 116 participants (S1 Table). Across all 116 retained participants, PDI scores were stable between the pre-screening and experimental sessions (ρ = 0.97) and both correlated strongly with the secondary measure of odd beliefs, the Paranoia Checklist (all ρ>0.79). Quality checks again suggested comparable quality to similar in-person studies (S14 Fig and S15 Fig).

#### Pre-Screening and PDI Classification

To ensure a wide enough range of odd beliefs and sufficient sampling of meaningfully high levels [54], study 2 pre-screened participants based on the PDI^22^. Participants with high or low belief oddity based on their PDI scores were invited for the experimental session, with the cutoffs based on reported norms for PDI global scores^47^ (mean of 58.9 and standard deviation of 48.0 in healthy individuals): mean plus 0.5 standard deviation (>82.9) for the high PDI group and mean minus 0.5 standard deviation (<34.9) for the low PDI group. For secondary analyses, we also invited participants with high (>17.15) or low (<6.65) frequency scores on reported norms for the Paranoia Checklist^92^. Participants who were invited solely based on the Paranoia Checklist scores were only included in exploratory dimensional analyses. Participants in the high and low PDI groups were gender- and age-matched (within 2 years). Those who completed the experimental session completed the PDI a second time (typically within 1–2 days of the pre-screening) and the mean PDI across both sessions was used for dimensional analyses.

Data from these participants was again high quality (S9 and S10 Figs).

#### Study 3

Study 3 re-analyzes combined data from studies 1 (n = 151) and 2 (n = 116) in 267 participants.

### Online data quality

In line with best practices for online studies [108–110], we limited recruitment (in study 1) to those with a high reputation [111] and a record of active engagement with tasks, we implemented strict exclusion criteria to ensure retention of participants who were most likely to have been actively and honestly engaged in our task (S1 Table), and we assessed and found evidence against selection bias (S2 Table and S3 Table). Attrition was consistent with previous work in online samples [112] and unlikely to compromise validity based on previous analyses [113]. We further confirmed that participant behavior was well captured by our model and that participants completed the task within a reasonable time frame, both consistent with our previous data from a related in-person study [31] (S14A Fig and S14C Fig). The precision of probability estimates was also consistent with previous in-person work, providing evidence that our incentive-compatible scoring method was effective (S14B Fig and S14D Fig). We also show that our parameter estimates were reliable and consistent within participants across the full duration of the task (S15 Fig). Finally, we conducted a noise-corrupted parameter recovery analysis showing that our results were unlikely to be driven by general low-level factors such as inattention or disengagement (Fig 6E and 6F). In line with previous online work [105,114,115], these analysis support that our data was valid, reliable, and high-quality.

### Model-agnostic measure of response variance

In keeping with the noisy-sampling model and previous work [2,17], we calculated the main measure of behavioral response variance in logit space as the variance of the log-odds of probability estimates for identical sequence fragments. The prediction of the noisy sampling model is that under identical circumstances, participants with a noisier prior representation will have greater variability in their posterior beliefs that will result in more response variability across instantiations. To isolate this variability, we determined the unique sets of sequence fragments, defined as subsequences of beads starting at the first bead that were identical in terms of bead-ratio condition and exact bead order (including bead color). For robustness, we only analyzed subsequences presented a minimum of 3 times (after excluding sequences with an incorrect final choice). (Note that the specific sequence fragments were identical in the order of the majority versus minority beads but differed in color across individuals, as the majority bead color of the hidden box was determined randomly for each subject.) For each given sequence fragment, we then calculated the variance of the logit estimates across different instantiations. We then calculated the median of variances across sequence fragments for a given bead-ratio condition, and calculated the mean of the medians across conditions to obtain the summary measure of response variance. We focus on this summary measure but our results hold separately for response variance measured separately by bead-ratio condition (S16 Fig).

### Statistical analysis

To analyze the probability estimates from the task, we employed parametric linear mixed-effects models, with random intercepts and slopes to account for within subject variance (Wilkinson Notation for all regressions is provided; see S1 Text). To minimize type 1 errors all linear mixed-effects models used the Satterthwaite correction for degrees of freedom [116]. To minimize disproportionate contributions of repeat sequences on results, the probability estimates for the only sequence that was repeated multiple times (i.e., the 8-majority bead sequence in the 90:10 bead-ratio condition) were averaged across for each participant and analyzed as a single sequence.

To analyze the relationship between model-agnostic summary measures (mean final estimate difference, the evidence asymmetry slope, prior-dependent updating slope, and response variance) and model-derived parameter values (see modeling below), we employed non-parametric tests because these variables were generally not normally distributed across participants based on Lilliefors tests at p<0.05. For group analyses of medians, we thus used sign-rank within-group tests and rank-sum between-group tests. Cliff’s delta (δ) was used as a non-parametric measure of effect size [117]. For dimensional analyses, we used Spearman correlations and partial Spearman correlations to control for potential confounding variables. All tests were considered statistically significant at p<0.05.

All analyses, including model-agnostic and model-based analyses, excluded trials with incorrect final choices, since these were unlikely to reflect inferential processes of interest and more likely to instead reflect model-unrelated lower-level factors such as inattention or task disengagement. The weighted Bayesian model predicts incorrect choices (due to evidence-order effects) at extreme levels of *ω*_1_. However, most of the errors we observed in our data were not predicted by this model based on fitted estimates (S17 Fig), suggesting that most errors were driven by lower-level factors like inattention. On average, this resulted in the exclusion of 1.5% trials per participant (S17A Fig). Less critically, the model goodness-of-fit was marginally improved after excluding trials with incorrect final choices (S17B Fig). Nonetheless the results of analyses including these trials were virtually unchanged.

Further, for all model-agnostic analyses involving the conversion of probability estimates to logit space, subjective probability estimates of 1 and 0 were excluded to avoid infinity values.

### Computational modeling

#### Weighted Bayesian belief-updating model and variants

We fit several weighted Bayesian belief updating models to the draw-by-draw probability estimates for each participant individually and extracted best-fitting parameters for each model. All models in the model comparison were variants of a weighted belief-updating model: *logit*(*posterior*) = *ω*_1_∙*logit*(*prior*)+*ω*_2_∙*logit*(*likelihood*).

In this model, *logit*(*prior*) represents the log-odds of the prior probability or belief on the current draw before integrating the likelihood, and it is equivalent to the posterior probability after the previous draw. *logit*(*likelihood*) represents the log-odds of the likelihood (or the log-likelihood ratio), which is the strength of the sensory evidence given by the bead-ratio for a specific bead draw with respect to the correct box. *logit*(*posterior*) represents the updated log-posterior ratio about the probability that the beads are coming from the green or blue box after combining the prior and the likelihood terms. The prior-weight *ω*_1_ is a free parameter that acts as a multiplicative weight on the prior belief; it affects how much older evidence is incorporated into the updated beliefs, controlling a primacy-recency bias. Prior underweighting (*ω*_1_ < 1) captures sequential base-rate neglect, limiting belief certainty (Fig 2C) and inducing a recency bias (Fig 2D and 2E) [2,4,17]. The likelihood-weight *ω*_2_ is a free parameter that scales the likelihood term multiplicatively and equally for older and newer samples of evidence, producing distinct effects from the prior-weight *ω*_1_ (S2D Fig).

Model fitting was performed for each subject using the Matlab function fmincon [118] in order to minimize the root mean squared error (RMSE) between the model-estimated probabilities and the probability estimates reported by the participant. Only estimates after bead draws were used for fitting, and the participant’s first estimate before the first bead draw defined the starting prior belief for a given trial. Data for sequences associated with an incorrect final decision were excluded from analyses. For robustness, participants’ data were each fit 100 times to each model, using random starting points between 0 and 20 for each free parameter. Bounds were set to 0 and 20. The parameters associated with the iteration yielding the lowest RMSE were taken as the best-fitting parameters for the participant and model. Formal model comparison (for the same 10 models used for comparison in our previous work [31]) was conducted based on the Schwarz Bayesian Information Criterion (BIC)[107]: $BIC=n∙\ln\left(\frac{\sum {error}^{2}}{n}\right)+l∙\ln\left(n\right)$, where n is the total number of fitted probability estimates (per participant), error is the difference between the actual probability estimates and the simulated probability estimates, and *l* is the number of parameters in the model. BIC values were used to calculate the protected exceedance probability (using the Variational Bayes Toolbox [119]; Fig 3D and S6 Fig) for group-level Bayesian model selection.

#### Noisy-sampling model

Azeredo da Silveira and Woodford [16] described a noisy-sampling model of belief updating where agents do not have access to the full prior distribution and instead represent prior beliefs imprecisely via noisy internal samples. Under this model, a rational response to imprecision in prior beliefs given the costs of precision and prediction inaccuracy is to underweight prior beliefs. This model thus provides a functional account for sequential base-rate neglect: lower prior-weight results from, and is inversely proportional to, noise in prior beliefs.

Here, we specify this model in the context of sequential belief-updating in our task. The noisy log-odds of the prior with respect to the true underlying state of the hidden box is:

$$r_{prior}=\log\left(\frac{\pi_{majority}}{\pi_{minority}}\right)+v_{p},{where\;v}_{p}∼N(0,\sigma_{prior}^{2}).$$

*r*_*prior*_ reflects the noisy internal representation of the prior. *π*_*majority*_ reflects the prior probability in favor of the true underlying state of the hidden box. *π*_*minority*_ reflects the prior probability in favor of the incorrect state of the hidden box, where *π*_*majority*_+*π*_*minority*_ = 1. *v*_*p*_ reflects the Gaussian noise (in logit space) of the internal representation of the prior, which is centered around 0 and has a variance of $\sigma_{prior}^{2}$. Similarly, the noisy log-odds of the likelihood with respect to the true underlying state of the hidden box is:

$$r_{likelihood}=\log\left(\frac{\lambda_{majority}}{\lambda_{minority}}\right)+v_{l},{where\;v}_{l}∼N(0,\sigma_{likelihood}^{2}).$$

*r*_*likelihood*_ reflects the noisy internal representation of the likelihood. *λ*_*majority*_ reflects the likelihood in probability space in favor of the correct state of the hidden box, and. *λ*_*minority*_ reflects the likelihood in favor of the incorrect state, where *λ*_*majority*_+*λ*_*minority*_ = 1. *v*_*l*_ reflects the Gaussian noise (in logit space) of the internal representation of the likelihood. The Gaussian noise is centered around zero and its variance may vary per bead-ratio condition, where $\sigma_{likelihood}^{2}$ can take on values $\sigma_{51}^{2},\sigma_{60}^{2}$, or $\sigma_{90}^{2}$ depending on the condition.

To calculate an optimal estimate of a participants’ beliefs in response to new evidence, we must also define a probability distribution over the possible true underlying states; that is, we must define the prior distributions from which the values {*π*, *λ*} may have been drawn. Here we define these distributions as centered around their corresponding probability ratio, $\log\left(\frac{\pi_{majority}}{\pi_{minority}}\right)\;or\;\log\left(\frac{\lambda_{majority}}{\lambda_{minority}}\right),$ where the variance of the distribution is given by $\omega_{prior}^{2}$ or $\omega_{likelihood}^{2}$. Further, $\omega_{likelihood}^{2}$ may similarly take on different values $\omega_{51}^{2}$, $\omega_{60}^{2}$ or $\omega_{90}^{2}$ per condition. The complete generative model over true possible situations and the participants’ noisy internal representations is specified by eight parameters: $\omega_{prior}^{2},\omega_{51}^{2},\omega_{60}^{2},\omega_{90}^{2},\sigma_{prior}^{2},\sigma_{51}^{2},\sigma_{60}^{2},and\;\sigma_{90}^{2}$.

Conditional on the prior (before the observation of a new bead draw), we can determine what optimal Bayesian inference of the true underlying state of the hidden box would be. The equations described above imply that the true log-odds (based on previously available information), $z_{prior}\equiv \log\left(\frac{\pi_{majority}}{\pi_{minority}}\right)$, and the prior have a joint, bivariate, Gaussian distribution. Consequently, the distribution of *z*_*prior*_ conditional on the noisy representation of the prior, *r*_*prior*_, will also be a Gaussian distribution:

$$z_{prior}|r_{prior}∼N(\gamma_{prior}∙r_{prior},\Sigma_{prior}^{2}),$$

$$\mathrm{where}\;\gamma_{prior}\equiv \frac{\omega_{prior}^{2}}{\omega_{prior}^{2}+\sigma_{prior}^{2}}\;\mathrm{and}\;\Sigma_{prior}^{2}\equiv \frac{\omega_{prior}^{2}∙\sigma_{prior}^{2}}{\omega_{prior}^{2}+\sigma_{prior}^{2}}.$$

From this, the implied probability that the true state of the hidden box is given by:

$$prior=\int \left(\frac{e^{z_{prior}}}{1+e^{z_{prior}}}\right)f(z_{prior}|r_{prior})dz_{prior},$$

where *f*(*z*_*prior*_|*r*_*prior*_) is the density function of the conditional distribution. In order to compute this quantity as a function of the *ω*^2^ and *σ*^2^ parameters, we use an analytical approximation[120] of this integral:

$$logit(prior)=\frac{\gamma_{prior∙r_{prior}}}{\rho_{prior}},where\;\rho_{prior}=\sqrt{1+(\frac{3}{\pi^{2}})∙\Sigma_{prior}^{2}}>1.$$

*ρ*_*prior*_ is a correction owing to the fact that the posterior distribution is not concentrated entirely at its mean. Then, we can substitute in the formula for prior to get:

$$logit(prior)=\frac{\gamma_{prior}}{\rho_{prior}}∙\log\left(\frac{\pi_{majority}}{\pi_{minority}}\right)+\epsilon_{prior},where\;\epsilon_{prior}∼N(0,{\left(\frac{\gamma_{prior}∙\sigma_{prior}}{\rho_{prior}}\right)}^{2}).$$

Here, we show the calculation for the prior, but the calculation of the likelihoods follows the same logic and can be obtained by substituting the corresponding *ω*^2^ and *σ*^2^ parameters. To calculate the posterior after each bead draw, we simply add or subtract the likelihood from the prior depending on if the bead is in favor of or against the true underlying state of the hidden box. If the signal is in favor of the true underlying state of the hidden box, the posterior would be:

$$logit(posterior)=\frac{\gamma_{prior}}{\rho_{prior}}∙\log\left(\frac{\pi_{majority}}{\pi_{minority}}\right)+\frac{\gamma_{likelihood}}{\rho_{likelihood}}∙\log\left(\frac{\lambda_{majority}}{\lambda_{minority}}\right)+\epsilon .$$

If the signal is against the true underlying state of the hidden box, the posterior would be:

$$logit(posterior)=\frac{\gamma_{prior}}{\rho_{prior}}∙\log\left(\frac{\pi_{majority}}{\pi_{minority}}\right)-\frac{\gamma_{likelihood}}{\rho_{likelihood}}∙\log\left(\frac{\lambda_{majority}}{\lambda_{minority}}\right)+\epsilon .$$

In either case, *ϵ* = *ϵ*_*prior*_ + $\epsilon_{likelihood}∼N(0,{\left(\frac{\gamma_{prior}∙\sigma_{prior}}{\rho_{prior}}\right)}^{2}+{\left(\frac{\gamma_{likelihood}∙\sigma_{likelihood}}{\rho_{likelihood}}\right)}^{2})$.

The *ω*_1_ and *ω*_2(*l*)_ parameters in the weighted Bayesian model correspond respectively to the weights $\frac{\gamma_{prior}}{\rho_{prior}}$ and $\frac{\gamma_{likelihood}}{\rho_{likelihood}}$ in the noisy-sampling model and are thus inversely proportional to the noise parameters, $\sigma_{prior}^{2}\;and\;\sigma_{likelihood}^{2}$, respectively. They are also inversely proportional to the parameters $\omega_{prior}^{2}$ and $\omega_{likelihood}^{2}$ representing the assumed uncertainty in the underlying logit prior and likelihood distributions. In visual schematic of the model in Fig 6, the prior weight is framed as the function $f(\sigma_{prior}^{2},\omega_{prior}^{2})=\frac{\gamma_{prior}}{\rho_{prior}}$, the likelihood weight as the function $f(\sigma_{likelihood}^{2},\omega_{likeliood}^{2})=\frac{\gamma_{likelihood}}{\rho_{likelihood}}$, and the noise parameters *ϵ* as the functions $g(\sigma_{prior}^{2},\omega_{prior}^{2})=\epsilon_{prior}$, and $g(\sigma_{likelihood}^{2},\omega_{likeliood}^{2})=\epsilon_{likelihood}$.

In fitting the model, consistent with previous work [121] we assumed that participants would adapt to the context of the task, acquiring a realistic estimate of the uncertainty underlying logit prior and likelihood distributions. Under this assumption $\omega_{prior}^{2}$ and $\omega_{likelihood}^{2}$ should be relatively constant across participants, and the primary source of interindividual variability should be reflected in the $\sigma_{prior}^{2}\;and\;\sigma_{likelihood}^{2}$ parameters. To avoid the possibility of parameter trade-off, our primary analysis fixed the $\omega_{prior}^{2}$ and the 3 $\omega_{likelihood}^{2}$ across the entire sample, but allowed the $\sigma_{prior}^{2}$ and the 3 $\sigma_{likelihood}^{2}$ parameters to vary freely. To determine the appropriate values for the $\omega_{prior}^{2}$ and the 3 $\omega_{likelihood}^{2}$ parameters, we conducted a 4-dimensional grid search of *ω*^2^ values from 0 to 1.4 in steps of 0.2, fitting the 4 *σ*^2^ parameters to each participant’s data for a given set of *ω*^2^ parameter values. To do this we used the Matlab function fmincon [118] in order to minimize the RMSE between the model-estimated probabilities and the probability estimates reported by the participant. We then calculated the group-level BIC (based on RMSE across all trials and participants) and selected the *ω*^2^ parameter values from the model with the lowest value; these were: $\omega_{prior}^{2}=0.2$, $\omega_{51}^{2}=0.2,\omega_{60}^{2}=0.2,and\;\omega_{90}^{2}=0.6$. Fitted *σ*^2^ parameter values from the noisy-sampling model with these fixed *ω*^2^ values were taken as best-fitting values and used in our main analyses. Since increased $\omega_{prior}^{2}$ and $\sigma_{prior}^{2}$ parameter values could both lead to decreased prior weighting and response variance under this model (S7 Fig), we empirically assessed the possibility that $\omega_{prior}^{2}$, and not $\sigma_{prior}^{2}$, could drive interindividual variability in base-rate neglect and response variance. Instead of using fixed $\omega_{prior}^{2}$ parameter values, we took the best-fitting values for each individual. Critically, these individually best-fitting $\omega_{prior}^{2}$ values were uncorrelated with the prior-weight *ω*_1_ from the weighted Bayesian model and our measure of response variance (S18 Fig). Furthermore, comparisons of $\omega_{prior}^{2}$ parameter values fitted for relevant subgroups (median-split groups based on *ω*_1_ or response variance) were inconsistent with an alternative explanation of base-rate neglect in terms of variability in $\omega_{prior}^{2}$.

#### Parameter recovery analysis

To generate simulated agents for parameter recovery, we sampled agent model parameters from the range of fitted parameters values found in the real data. Specifically, we randomly sampled parameters uniformly from the 10^th^ to 90^th^ percentile of values to limit the influence of extreme values. Responses were then simulated on the experimental trials that participants observed. Simulated observers started each trial with unbiased prior beliefs about the hidden box and posterior beliefs after each draw were updated in logit space according to the model. To evaluate the robustness of model fitting procedures to late (e.g., motor) noise, varying magnitudes of zero-mean Gaussian noise were added to the logit posterior beliefs after updating. This late noise was unrelated to the inference process, and thus only affected the agents’ reported noisy estimates and did not propagate to subsequent prior beliefs. To simulate realistic levels of late Gaussian noise, we estimated the variance that matched the variability observed in the data. First, we determined the 95% confidence interval of the median response variance at the group level in the actual data via bootstrapping. Next, we simulated ten sets of random agents (n = 267 per set, as in the combined dataset for study 3) across a range of late-noise variance levels. For each level, we calculated the median response variance at the group level and the mean of the medians across the sets. We determined the estimated noise range of the actual data to correspond to noise levels where this mean of medians overlapped with the 95% confidence interval of the median response variance in the actual data.

## Supporting information

S1 Table Sociodemographic and clinical characteristics of samples included in data analysis. (DOCX)

S2 Table Sociodemographic and clinical characteristics for study 1 comparing included participants to non-completers and excluded participants. (DOCX)

S3 Table Sociodemographic and clinical characteristics for study 2 comparing included participants to non-completers and excluded participants. (DOCX)

S4 Table Linear mixed-effects model predicting probability estimates based on bead draw and bead ratio. (DOCX)

S5 Table Linear mixed-effects model predicting probability estimates based on bead draw and bead ratio for matched trials. (DOCX)

S6 Table Linear mixed-effects model predicting final estimate difference based on evidence asymmetry and bead ratio. (DOCX)

S7 Table Linear mixed-effects model predicting mean logit-belief updates based on mean logit-priors and bead ratio. (DOCX)

S8 Table Descriptive statistics for the parameters of the winning weighted Bayesian model for study 1, 2, and 3. (DOCX)

S9 Table Pair-wise correlations between PDI score, the final estimate difference, the Evidence Asymmetry Slope, the prior dependent updating slope, and *ω*_1_. (DOCX)

S10 Table Linear model predicting participant scores on the 9-item Raven’s Matrix based on their fitted parameters from the weighted Bayesian model (N = 143). (DOCX)

S11 Table Linear model predicting participant scores on their anxious-depression factor score (S4 Fig) based on their fitted parameters from the weighted Bayesian model (N = 143). (DOCX)

S12 Table Linear model predicting participant scores on their OCD factor score (S4 Fig) based on their fitted parameters from the weighted Bayesian model (N = 143). (DOCX)

S13 Table Linear model predicting participant scores on their social withdrawal factor score (S4 Fig) based on their fitted parameters from the weighted Bayesian model (N = 143). (DOCX)

S14 Table Linear mixed-effects model predicting probability estimates based on bead draw and bead ratio for the main sample in study 2 (N = 91). (DOCX)

S15 Table Linear mixed-effects model predicting probability estimates based on bead draw and bead ratio for the main sample in study 2 (N = 91) for matched trials. (DOCX)

S16 Table Linear mixed-effects model predicting final estimate difference based on evidence asymmetry and bead ratio for the main sample in study 2 (N = 91). (DOCX)

S17 Table Linear mixed-effects model predicting mean logit-belief updates based on mean logit-priors and bead ratio for the main sample in study 2 (N = 91). (DOCX)

S18 Table Linear mixed-effects model predicting final estimate difference based on evidence asymmetry and bead ratio for low PDI group only (N = 57). (DOCX)

S19 Table Linear mixed-effects model predicting final estimate difference based on evidence asymmetry and bead ratio for the high PDI group only (N = 34). (DOCX)

S20 Table Linear mixed-effects model predicting mean logit-belief updates based on mean logit-priors and bead ratio for the low PDI group only (N = 57). (DOCX)

S21 Table Linear mixed-effects model predicting mean logit-belief updates based on mean logit-priors and bead ratio for the high PDI group only (N = 34). (DOCX)

S22 Table Statistics for rank sum tests for group differences between Low (N = 34) and High (N = 57) PDI groups for belief updating measures yielded by study 2. (DOCX)

S23 Table Pair-wise correlations for study 2 between mean PDI score (mean of prescreening and experimental session PDI scores; see Methods), Paranoia Checklist score, the final estimate difference, the evidence asymmetry slope, the prior dependent updating slope, and *ω*_1_. (DOCX)

S24 Table Correlations between measures of response variability and *ω*_1_ by individual study and for the full sample. (DOCX)

S25 Table Descriptive statistics for the parameters of the noisy sampling model for study 3. (DOCX)

S26 Table Statistics for rank sum tests for group differences between Low (N = 34) and High (N = 57) PDI groups for belief updating measures yielded by study 3. (DOCX)

S27 Table Prior weight *ω*_1_ is not associated with response times. (DOCX)

S28 Table Weighted Bayesian belief updating models. (DOCX)

S29 Table PDI items and order of presentation of items. (DOCX)

S30 Table Bead sequences (i.e., trials) used in studies 1 and 2. (DOCX)

S31 Table Questions and possible responses during the miscomprehension quiz. (DOCX)

S1 Fig Evidence asymmetry is unrelated to mean final estimates. (DOCX)

S2 Fig Condition-wise simulations of the final estimate difference and prior-dependent updating as a function of *ω*_1_ and *ω*_2_. (DOCX)

S3 Fig Probability estimates before presentation of the first bead (i.e., at the 0^th^ bead). (DOCX)

S4 Fig Comparison of sample’s general psychopathology factor scores to those in Gillan et al (2016). (DOCX)

S5 Fig Logit-belief updates as a function of logit prior by bead ratio for the main sample in study 2 (N = 91). (DOCX)

S6 Fig Formal model comparison for data from (a) study 1 and (b) study 2.
(DOCX)

S7 Fig Predicted relationships between parameters governing prior integration and response variability in (a) a volatility model and (b) the noisy sampling model. (DOCX)

S8 Fig Scatterplots and Spearman correlations of corresponding likelihood parameters from the weighted Bayesian model and noisy sampling model. (DOCX)

S9 Fig Simulations varying individual *ω*^2^ parameters in the noisy sampling model, while holding all other parameters constant. (DOCX)

S10 Fig Posterior predictive checks for mean final estimate difference and evidence asymmetry slope. (DOCX)

S11 Fig Posterior predictive checks for logit belief updates. (DOCX)

S12 Fig Posterior predictive checks for response variance. (DOCX)

S13 Fig The binarized scoring rule maximizes expected value for accurate probability estimates. (DOCX)

S14 Fig Data Quality 1: Comparison between the weighted Bayesian model mean squared error and total time taken to complete the probability estimates beads task. (DOCX)

S15 Fig Data Quality 2: Evidence for behavioral consistency across the probability estimates beads task. (DOCX)

S16 Fig The relationship between the condition-wise response variance and *ω*_1_. (DOCX)

S17 Fig Trial-wise exclusions are justified because few box choice errors are predicted by the weighted Bayesian model. (DOCX)

S18 FigNegligible effects of $\omega_{prior}^{2}$ on base-rate neglect and response variance.(DOCX)

S1 Text Supplemental Materials table of contents. (DOCX)

S2 Text Direct replication of results from study 1 using Study 2 data. (DOCX)

S3 Text Volatility Model Specification. (DOCX)

S1 Movie Video demonstration of task instructions, miscomprehension quiz, and practice trials. (WEBM)
