# Supplementary material for: The effects of base rate neglect on sequential belief updating and real-world beliefs
Source: PLoS Comput Biol. 2022 Dec 22;18(12):e1010796. doi: 10.1371/journal.pcbi.1010796 (PMC9831339; doi:10.1371/journal.pcbi.1010796)
Supplement: S1 Table — (DOCX) [file pcbi.1010796.s001.docx]

**S1 Table. Sociodemographic and clinical characteristics of samples included in data analysis.** All p-values reflect the statistical difference between the Low and High PDI group from Study 2. For clinical characteristics, the p-values correspond to non-parametric rank-sum tests, as the clinical measures were non-normally distributed (Lilliefors’s test; p < 0.05 for all clinical measures).

| *Basic Demographics* | Study 1: All Participants | Study 2: All Participants | Study 2: Low PDI Group | Study 2: High PDI Group | Low vs. High p-Value |
| --- | --- | --- | --- | --- | --- |
| N ^a^ | 151 | 116 | 57 | 34 |  |
| Age (Mean) ^a^ | 39.12 | 35.51 | 36.55 | 34.61 | 0.197 |
| Biological Sex (Female/Male) ** | 78/73 | 67/49 | 36/21 | 18/16 | 0.337 |
| *Race* ^b^ | | | | | |
| African-American | 9 | 9 | 6 | 2 | 0.477 |
| Asian | 6 | 6 | 5 | 1 |  |
| Caucasian | 127 | 94 | 42 | 29 |  |
| Hispanic | 5 | 5 | 3 | 1 |  |
| Other/Mixed | 2 | 1 | 0 | 1 |  |
| Prefer Not to Answer | 2 | 1 | 1 | 0 |  |
| *Education* ^b^ | | | | | |
| Partial High School (10th or 11th Grade) | 1 | 2 | 1 | 0 | 0.449 |
| High School Graduate | 22 | 14 | 5 | 5 |  |
| Partial College or Specialized Training | 37 | 31 | 13 | 12 |  |
| Standard College or University Graduate | 76 | 55 | 29 | 14 |  |
| Graduate Professional Training | 15 | 14 | 9 | 3 |  |
| *Other/Mixed* ^b^ | | | | | |
| Hispanic Ethnicity (Yes/No) | 7/144 |  |  |  |  |
| Handedness (Right/Left/Both) | 133/13/5 | 99/15/2 | 52/4/1 | 24/9/1 | 0.032 |
| Smoker? (Yes/No) | 25/126 | 39/77 | 20/37 | 11/23 | 0.790 |
| Drug User? (Yes/No) | 12/139 | 80/36 | 40/17 | 20/14 | 0.269 |
| *Psychiatric History* ^b^ | | | | | |
| Ever Hospitalized for Psychiatric Problems? (Yes/No) | 6/145 | 16/100 | 6/51 | 8/26 | 0.096 |
| *Current Psychiatric Diagnosis?* |  |  |  |  |  |
| Yes, in the last 6 months | 6 | 19 | 8 | 8 | 0.510 |
| Yes, but not in the last 6 months | 34 | 26 | 14 | 7 |  |
| No | 111 | 71 | 35 | 19 |  |
| *Neurological History* ^b^ | | | | | |
| Ever Hospitalized for Neurological Problems? (Yes/No) | 1/150 | 5/111 | 4/53 | 1/33 | 0.409 |
| *Current Neurological Diagnosis?* |  |  |  |  |  |
| Yes, in the last 6 months | 0 | 2 | 1 | 1 | 0.915 |
| Yes, but not in the last 6 months | 1 | 7 | 4 | 2 |  |
| No | 150 | 107 | 52 | 31 |  |
| *Measures of Odd Beliefs and Perceptions* ^c^ | | | | | |
| Prescreening PDI Global, median |  | 38.5 | 6 | 117.5 | 1.34 x 10^-15^ |
| Prescreening PDI Global, range |  | 0 - 240 | 0 - 33 | 85 - 240 |  |
| Experimental Session PDI Global, median | 20 | 34.5 | 7 | 102.5 | 1.95 x 10^-15^ |
| Experimental Session PDI Global, range | 0 - 104 | 0 - 240 | 0 - 75 | 64 - 240 |  |
| Mean PDI Global, median |  | 36 | 8 | 110 | 1.56 x 10^-15^ |
| Mean PDI Global, range |  | 0 - 240 | 0 - 53.5 | 78.5 - 240 |  |
| CAPS Global, median ^d^ | 15 | 26 (27) | 5 | 121 (121.5) | 1.48 x 10^-12^  (7.66 x 10^-13^) |
| CAPS Global, range ^d^ | 0 - 158 | 0 - 272 (417) | 0 - 98 | 15 - 272 (417) |  |
| Paranoia Checklist, median |  | 9 | 1 | 27 | 3.37 x 10^-15^ |
| Paranoia Checklist, range |  | 0 - 64 | 0 - 32 | 7 - 64 |  |
| ^a^ *p-value reflects significance for t-test* | | | | | |
| ^b^ *p-value reflects chi-squared test for independence* | | | | | |
| ^c^ *p-value reflects rank-sum test*  *^d^ Values reflect CAPS scores with (in parentheses) and without including a single outlier*  *^d^ Values reflect CAPS scores with (in parentheses) and without including a single outlier* | | | | | |
| *^d^ Values reflect CAPS scores with (in parentheses) and without including a single outlier* | | | | | |
