# Supplementary material for: The effects of base rate neglect on sequential belief updating and real-world beliefs
Source: PLoS Comput Biol. 2022 Dec 22;18(12):e1010796. doi: 10.1371/journal.pcbi.1010796 (PMC9831339; doi:10.1371/journal.pcbi.1010796)
Supplement: S2 Table — (DOCX) [file pcbi.1010796.s002.docx]

**S2 Table. Sociodemographic and clinical characteristics for study 1 comparing included participants to non-completers and excluded participants.** There were no major differences between these populations, suggesting that our online recruitment procedures did not induce a selection bias. ‘Completers’ are participants who completed all elements of the study and were included in data analysis. ‘Excluded’ participants are those who completed all study procedures but were excluded from analysis due to poor performance. ‘Non-completers’ were participants who began the study, completed the demographic and clinical questionnaires, but quit the study before completing the main task.

|  | Study 1 | Completers vs Excluded | | | Completers vs Non-Completers | | |
| --- | --- | --- | --- | --- | --- | --- | --- |
| *Basic Demographics* | Completers | Excluded | P-Value | Effect Size | Non-Completers | P-Value | Effect Size |
| N ^a^ | 151 | 19 |  |  | 43 |  |  |
| Age (mean) ^a^ | 39.1211 | 41.5 | 0.744 | -0.080 | 37.4 | 0.246 | 0.201 |
| Biological Sex (Female/Male) ^b^ | 78/73 | 9/10 | 0.725 | 0.027 | 29/14 | 0.066 | 0.132 |
| *Race* ^b^ | | | | | | | |
| African-American | 9 | 1 | 0.807 | 0.116 | 1 | 0.552 | 0.143 |
| Asian | 6 | 2 |  |  | 3 |  |  |
| Caucasian | 127 | 15 |  |  | 36 |  |  |
| Hispanic | 5 | 1 |  |  | 1 |  |  |
| Other/Mixed | 2 | 0 |  |  | 2 |  |  |
| Prefer Not To Answer | 2 | 0 |  |  | 0 |  |  |
| *Education* ^b^ | | | | | | | |
| Partial High School (10th or 11th Grade) | 1 | 0 | 0.064 | 0.229 | 0 | 0.532 | 0.128 |
| High School Graduate | 22 | 4 |  |  | 3 |  |  |
| Partial College or Specialized Training | 37 | 10 |  |  | 15 |  |  |
| Standard College or University Graduate | 76 | 4 |  |  | 21 |  |  |
| Graduate Professional Training | 15 | 1 |  |  | 4 |  |  |
| *Other/Mixed* ^b^ | | | | | | | |
| Hispanic Ethnicity (Yes/No) | 7/144 | 2/17 | 0.2798 | 0.0829 | 1/42 | 0.502 | 0.048 |
| Handedness (R/L/A) | 133/13/5 | 15/2/2 | 0.9155 | 0.0313 | 37/6/0 | 0.299 | 0.112 |
| Smoker? (Yes/No) | 25/126 | 5/14 | 0.2929 | 0.0807 | 8/35 | 0.753 | 0.023 |
| Drug User? (Yes/No) | 12/139 | 1/18 | 0.6782 | 0.0318 | 7/36 | 0.105 | 0.116 |
| *Psychiatric History* ^b^ | | | | | | | |
| Ever Hospitalized for Psychiatric Problems? (Yes/No) | 6/145 | 0/19 | 0.3764 | 0.0678 | 0/43 | 0.184 | 0.095 |
| *Current Psychiatric Diagnosis?* |  |  |  |  |  |  |  |
| Yes, in the last 6 months | 6 | 1 | 0.069 | 0.1774 | 0 | 0.109 | 0.150 |
| Yes, but not in the last 6 months | 34 | 0 |  |  | 6 |  |  |
| No | 111 | 18 |  |  | 37 |  |  |
| *Neurological History* ^b^ | | | | | | | |
| Ever Hospitalized for Neurological Problems? (Yes/No) | 1/150 | 0/19 | 0.722 | 0.0273 | 0/43 | 0.593 | 0.038 |
| *Current Neurological Diagnosis?* |  |  | 10 |  |  |  |  |
| Yes, in the last 6 months | 0 | 0 | 0.0796 | 0.1344 | 0 |  |  |
| Yes, but not in the last 6 months | 1 | 1 |  |  | 2 | 0.061 | 0.134 |
| No | 150 | 18 |  |  | 41 |  |  |
| *Measures of Odd Beliefs and Perceptions* ^c^ | | | | | | | |
| PDI Global, median - 1st time | 20 | 10 | 0.4721 | 0.1014 | 17 | 0.484 | 0.070 |
| PDI Global, range - 1st time | 0 - 104 | 0 - 153 |  |  | 0 - 172 |  |  |
| CAPS Global, median | 15 | 14 | 0.8292 | -0.0303 | 7 | 0.329 | 0.096 |
| CAPS Global, range | 0 - 158 | 0 - 231 |  |  | 0 - 194 |  |  |
| ^a^ *p-value reflects significance for t-test, effect size reflects Cohen’s d* | | | | | | | |
| ^b^ *p-value reflects chi-squared test for independence, effect size reflects phi* | | | | | | | |
