# Supplementary material for: The effects of base rate neglect on sequential belief updating and real-world beliefs
Source: PLoS Comput Biol. 2022 Dec 22;18(12):e1010796. doi: 10.1371/journal.pcbi.1010796 (PMC9831339; doi:10.1371/journal.pcbi.1010796)
Supplement: S4 Table — (DOCX) [file pcbi.1010796.s004.docx]

**S4 Table. Linear mixed-effects model predicting probability estimates based on bead draw and bead ratio.**

This analysis corresponds to Fig 3a in the main text.

Wilkinson Notation: Estimates ~ Draw*Ratio +(Draw*Ratio|Subject_Number).

| **Effect** | **Estimate** | ***SE*** | ***t-stat*** | **df** | ***p*** | **95% CI** | |
| --- | --- | --- | --- | --- | --- | --- | --- |
|  |  |  |  |  |  | ***LL*** | ***UL*** |
| Intercept | 0.449 | 0.006 | 75.528 | 237.37 | 5.51e-168 | 0.437 | 0.461 |
| Bead Draw | 0.066 | 0.010 | 6.904 | 188.86 | 7.47e-11 | 0.047 | 0.084 |
| Bead Ratio | -0.009 | 0.002 | -4.231 | 156.46 | 3.96e-05 | -0.013 | -0.005 |
| Bead Draw * Bead Ratio | 0.066 | 0.003 | 26.152 | 160.66 | 8.82e-60 | 0.061 | 0.071 |
| Adj. R2 = 0.4218 |  |  |  |  |  |  |  |
|  |  |  |  |  |  |  |  |
