# Supplementary material for: The effects of base rate neglect on sequential belief updating and real-world beliefs
Source: PLoS Comput Biol. 2022 Dec 22;18(12):e1010796. doi: 10.1371/journal.pcbi.1010796 (PMC9831339; doi:10.1371/journal.pcbi.1010796)
Supplement: S5 Table — (DOCX) [file pcbi.1010796.s005.docx]

**Table S5. Linear mixed-effects model predicting probability estimates based on bead draw and bead ratio for matched trials.**

This analysis only includes the 16 trials that were matched between the 60:40 and 90:10 bead ratio conditions.

This analysis corresponds to the inset of Fig 3a in the main text.

Wilkinson Notation: Estimates ~ Draw*Ratio +(Draw*Ratio|Subject_Number).

| **Effect** | **Estimate** | ***SE*** | ***t-stat*** | **df** | ***p*** | **95% CI** | |
| --- | --- | --- | --- | --- | --- | --- | --- |
|  |  |  |  |  |  | ***LL*** | ***UL*** |
| Intercept | 0.481 | 0.008 | 63.027 | 311.99 | 1.54e-179 | 0.466 | 0.496 |
| Bead Draw | 0.031 | 0.010 | 2.974 | 282.82 | 3.19e-03 | 0.010 | 0.051 |
| Bead Ratio | 0.021 | 0.002 | 8.598 | 164.55 | 6.08e-15 | 0.016 | 0.026 |
| Bead Draw * Bead Ratio | 0.031 | 0.003 | 11.807 | 182.96 | 2.79e-24 | 0.026 | 0.036 |
| Adj. R2 = 0.4528 |  |  |  |  |  |  |  |
|  |  |  |  |  |  |  |  |
