# Supplementary material for: The effects of base rate neglect on sequential belief updating and real-world beliefs
Source: PLoS Comput Biol. 2022 Dec 22;18(12):e1010796. doi: 10.1371/journal.pcbi.1010796 (PMC9831339; doi:10.1371/journal.pcbi.1010796)
Supplement: S6 Table — (DOCX) [file pcbi.1010796.s006.docx]

**S6 Table. Linear mixed-effects model predicting final estimate difference based on evidence asymmetry and bead ratio**.

This analysis corresponds to Fig 3b in the main text.

Wilkinson Notation: Final Estimate Difference ~ Ratio* Evidence Asymmetry +(Ratio*Evidence Asymmetry|Subject_Number).

| **Effect** | **Estimate** | ***SE*** | ***t-stat*** | **df** | ***p*** | **95% CI** | |
| --- | --- | --- | --- | --- | --- | --- | --- |
|  |  |  |  |  |  | ***LL*** | ***UL*** |
| Intercept | 0.036 | 0.023 | 1.583 | 149.26 | 0.116 | -0.009 | 0.080 |
| Evidence Asymmetry | -0.006 | 0.005 | -1.233 | 148.74 | 0.219 | -0.016 | 0.004 |
| Bead Ratio | -0.001 | 3.007e-04 | -1.921 | 165.58 | 0.056 | -0.001 | 1.612e-05 |
| Evidence Asymmetry * Bead Ratio | 2.246e-04 | 7.230e-05 | 3.107 | 151.46 | 0.002 | 8.178e-05 | 3.675e-04 |
| Adj. R2 = 0.2183 |  |  |  |  |  |  |  |
|  |  |  |  |  |  |  |  |
