# Supplementary material for: The effects of base rate neglect on sequential belief updating and real-world beliefs
Source: PLoS Comput Biol. 2022 Dec 22;18(12):e1010796. doi: 10.1371/journal.pcbi.1010796 (PMC9831339; doi:10.1371/journal.pcbi.1010796)
Supplement: S7 Table — (DOCX) [file pcbi.1010796.s007.docx]

**S7 Table. Linear mixed-effects model predicting mean logit-belief updates based on mean logit-priors and bead ratio**.

This analysis corresponds to Fig 3c in the main text.

Wilkinson Notation: Logit Belief Update ~ Logit Prior*Ratio +(Logit Prior*Ratio|Subject_Number).

| **Effect** | **Estimate** | ***SE*** | ***t-stat*** | **df** | ***p*** | **95% CI** | |
| --- | --- | --- | --- | --- | --- | --- | --- |
|  |  |  |  |  |  | ***LL*** | ***UL*** |
| Intercept | 0.255 | 0.015 | 17.205 | 149.80 | 2.664e-37 | 0.226 | 0.285 |
| Logit-Prior | -0.056 | 0.021 | -2.643 | 150.30 | 0.009 | -0.099 | -0.014 |
| Bead Ratio | 0.173 | 0.012 | 14.582 | 146.91 | 2.392e-30 | 0.149 | 0.196 |
| Logit-Prior * Bead Ratio | 0.006 | 0.007 | 0.903 | 143.13 | 0.368 | -0.008 | 0.020 |
| Adj. R2 = 0.3097 |  |  |  |  |  |  |  |
