# Supplementary material for: The effects of base rate neglect on sequential belief updating and real-world beliefs
Source: PLoS Comput Biol. 2022 Dec 22;18(12):e1010796. doi: 10.1371/journal.pcbi.1010796 (PMC9831339; doi:10.1371/journal.pcbi.1010796)
Supplement: S8 Table — (DOCX) [file pcbi.1010796.s008.docx]

**S8 Table. Descriptive statistics for the parameters of the winning weighted Bayesian model for study 1, 2, and 3.**

| Study 1 (n = 151) | Mean | Standard Deviation | Median | 25th Percentile | 75th percentile |
| --- | --- | --- | --- | --- | --- |
| ω_1_ | 0.950 | 0.143 | 0.983 | 0.908 | 1.025 |
| ω _2 (51:49)_ | 4.281 | 4.152 | 3.085 | 1.143 | 5.699 |
| ω _2 (60:40)_ | 0.970 | 1.530 | 0.803 | 0.577 | 1.030 |
| ω _2 (90:10)_ | 0.299 | 0.208 | 0.246 | 0.159 | 0.336 |
|  |  |  |  |  |  |
| Study 2 (n = 116) | Mean | Standard Deviation | Median | 25th Percentile | 75th percentile |
| ω_1_ | 0.926 | 0.213 | 0.982 | 0.944 | 1.018 |
| ω _2 (51:49)_ | 4.138 | 4.531 | 2.571 | 0.884 | 5.780 |
| ω _2 (60:40)_ | 0.985 | 1.100 | 0.816 | 0.567 | 1.047 |
| ω _2 (90:10)_ | 0.447 | 1.224 | 0.254 | 0.183 | 0.400 |
|  |  |  |  |  |  |
| Study 3 (n = 267) | Mean | Standard Deviation | Median | 25th Percentile | 75th percentile |
| ω_1_ | 0.940 | 0.177 | 0.983 | 0.919 | 1.022 |
| ω _2 (51:49)_ | 4.219 | 4.313 | 2.745 | 1.007 | 5.749 |
| ω _2 (60:40)_ | 0.976 | 1.358 | 0.808 | 0.570 | 1.036 |
| ω _2 (90:10)_ | 0.364 | 0.823 | 0.247 | 0.168 | 0.379 |
