# Supplementary material for: The effects of base rate neglect on sequential belief updating and real-world beliefs
Source: PLoS Comput Biol. 2022 Dec 22;18(12):e1010796. doi: 10.1371/journal.pcbi.1010796 (PMC9831339; doi:10.1371/journal.pcbi.1010796)
Supplement: S9 Table — (DOCX) [file pcbi.1010796.s009.docx]

**S9 Table.** **Pair-wise correlations between PDI score, the final estimate difference, the evidence asymmetry slope, the prior dependent updating slope, and** $\boldsymbol{\omega}_{\boldsymbol{1}}$. PDU bias was defined as the logit-prior beta values for individual-level linear mixed-effects analysis consistent with S7 Table, but conducted for each participant individually (i.e., overall participant slopes consistent with Figure 3c). Consistent with earlier analyses, to calculate the prior-dependent updating slope, probability estimates of exactly 1 and 0 were excluded. Partial correlations control for the three $\omega_{2_{(likelihood)}}$ parameters, and the model root-mean-squared-error and are only reported for correlations that involve relevant model parameters such as $\omega_{1}$. n = 151 for all correlations.

| **Correlation Table** | | | | | | | | | | |
| --- | --- | --- | --- | --- | --- | --- | --- | --- | --- | --- |
| Correlations between indices of base-rate neglect | | | | **Spearman** | | | **Partial Spearman** | | | |
|  |  |  |  | **rho** | | **p** | **rho** | **p** | | |
| Final Estimate Difference | | - | Evidence Asymmetry Slope | 0.633 | *** | 2.2251e-308 | N/A |  | N/A |  |
| Final Estimate Difference | | - | Prior Dependent Updating Slope | -0.489 | *** | 2.5765e-10 | N/A |  | N/A |  |
| Final Estimate Difference | | - | ω_1_ | -0.604 | *** | 2.2251e-308 | -0.603 | *** | 6.8204e-16 |  |
| Evidence Asymmetry Slope | | - | Prior Dependent Updating Slope | -0.495 | *** | 1.3194e-10 | N/A |  | N/A |  |
| Evidence Asymmetry Slope | | - | ω_1_ | -0.642 | *** | 2.2251e-308 | -0.612 | *** | 1.7142e-16 |  |
| PDU | | - | ω_1_ | 0.707 | *** | 2.2251e-308 | 0.629 | *** | 1.3591e-17 |  |
|  | | | | | | | | | | |
| Correlations between indices of base-rate neglect and PDI | | | | **Spearman** | | | **Partial Spearman** | | | |
|  |  |  |  | **rho** | | **p** | **rho** | **p** | | |
| PDI | - | Final Estimate Difference | | 0.094 |  | 0.2494 | N/A |  | N/A |  |
| PDI | - | Evidence Asymmetry Slope | | 0.131 |  | 0.1069 | N/A |  | N/A |  |
| PDI | - | Prior Dependent Updating Slope | | -0.012 |  | 0.8805 | N/A |  | N/A |  |
| PDI | - | ω_1_ | | 0.007 |  | 0.928 | -0.017 |  | 0.8413 |  |
|  | | | | | | | | | | |
| * p < 0.05, ** p < 0.01, *** p < 0.001 | | | | | | | | | | |
