# Supplementary material for: The effects of base rate neglect on sequential belief updating and real-world beliefs
Source: PLoS Comput Biol. 2022 Dec 22;18(12):e1010796. doi: 10.1371/journal.pcbi.1010796 (PMC9831339; doi:10.1371/journal.pcbi.1010796)
Supplement: S10 Table — (DOCX) [file pcbi.1010796.s010.docx]

**S10 Table. Linear model predicting participant scores on the 9-item Raven’s Matrix based on their fitted parameters from the weighted Bayesian model (N = 143)**. The Raven’s score is associated with $\omega_{1}$, $\omega_{2_{(51:49)}}$, $\omega_{2_{(60:40)}}$, and $\omega_{2_{(90:10)}}$. Critically, it is not specifically associated with the prior-weight parameter $\omega_{1}$ and it is most strongly associated with $\omega_{2_{(60:40)}}$. Therefore, variation in general cognition does not appear to be specifically driving interindividual differences in $\omega_{1}$. Wilkinson Notation: Raven’s Score ~ $\omega_{1}$ + $\omega_{2_{(51:49)}}$+ $\omega_{2_{(60:40)}}$ + $\omega_{2_{(90:10)}}$.

| **Effect** | **Estimate** | ***SE*** | ***t-stat*** | **df** | ***p*** | **95% CI** | |
| --- | --- | --- | --- | --- | --- | --- | --- |
|  |  |  |  |  |  | ***LL*** | ***UL*** |
| Intercept | 34.132 | 6.080 | 5.614 | 138 | 1.0467e-07 | 22.110 | 46.153 |
| ω_1_ | 11.838 | 5.619 | 2.107 | 138 | 0.037 | 0.728 | 22.949 |
| ω _2 (51:49)_ | -0.332 | 0.194 | -1.711 | 138 | 0.089 | -0.715 | 0.052 |
| ω _2 (60:40)_ | -2.107 | 0.506 | -4.163 | 138 | 5.4956e-05 | -3.107 | -1.106 |
| ω _2 (90:10)_ | 11.930 | 4.212 | 2.832 | 138 | 0.005 | 3.601 | 20.258 |
| Adj. R2 = 0.1491 | |  |  |  |  |  |  |
|  | |  |  |  |  |  |  |
|  | |  |  |  |  |  |  |
