# Supplementary material for: The effects of base rate neglect on sequential belief updating and real-world beliefs
Source: PLoS Comput Biol. 2022 Dec 22;18(12):e1010796. doi: 10.1371/journal.pcbi.1010796 (PMC9831339; doi:10.1371/journal.pcbi.1010796)
Supplement: S11 Table — (DOCX) [file pcbi.1010796.s011.docx]

**Table S11. Linear model predicting participant scores on their Anxious-Depression Factor Score (S3 Fig) based on their fitted parameters from the weighted Bayesian model (N = 143).** The factor score is not associated with any model parameters. Therefore, variation in the anxious-depression factor does not appear to be specifically driving interindividual differences in $\omega_{1}$. Wilkinson Notation: Anxious Depression Factor Score ~ $\omega_{1}$ + $\omega_{2_{(51:49)}}$+ $\omega_{2_{(60:40)}}$ + $\omega_{2_{(90:10)}}$.

| **Effect** | **Estimate** | ***SE*** | ***t-stat*** | **df** | ***p*** | **95% CI** | |
| --- | --- | --- | --- | --- | --- | --- | --- |
|  |  |  |  |  |  | ***LL*** | ***UL*** |
| Intercept | 1.502 | 0.693 | 2.169 | 138 | 0.032 | 0.132 | 2.871 |
| ω_1_ | 0.978 | 0.640 | 1.528 | 138 | 0.129 | -0.288 | 2.244 |
| ω _2 (51:49)_ | 0.013 | 0.022 | 0.585 | 138 | 0.559 | -0.031 | 0.057 |
| ω _2 (60:40)_ | -0.034 | 0.058 | -0.583 | 138 | 0.561 | -0.148 | 0.080 |
| ω _2 (90:10)_ | 0.110 | 0.480 | 0.229 | 138 | 0.819 | -0.839 | 1.059 |
| Adj. R2 = -0.0087 | |  |  |  |  |  |  |
