# Supplementary material for: The effects of base rate neglect on sequential belief updating and real-world beliefs
Source: PLoS Comput Biol. 2022 Dec 22;18(12):e1010796. doi: 10.1371/journal.pcbi.1010796 (PMC9831339; doi:10.1371/journal.pcbi.1010796)
Supplement: S12 Table — (DOCX) [file pcbi.1010796.s012.docx]

**Table S12. Linear model predicting participant scores on their OCD Factor Score (S3 Fig) based on their fitted parameters from the weighted Bayesian model (N = 143).** The factor score is not associated with any model parameters. Therefore, variation in the OCD factor does not appear to be specifically driving interindividual differences in $\omega_{1}$. Wilkinson Notation: OCD Factor Score ~ $\omega_{1}$ + $\omega_{2_{(51:49)}}$+ $\omega_{2_{(60:40)}}$ + $\omega_{2_{(90:10)}}$.

| **Effect** | **Estimate** | ***SE*** | ***t-stat*** | **df** | ***p*** | **95% CI** | |
| --- | --- | --- | --- | --- | --- | --- | --- |
|  |  |  |  |  |  | ***LL*** | ***UL*** |
| Intercept | 1.073 | 0.604 | 1.778 | 138 | 0.078 | -0.120 | 2.267 |
| ω_1_ | 0.469 | 0.558 | 0.840 | 138 | 0.402 | -0.634 | 1.572 |
| ω _2 (51:49)_ | 0.035 | 0.019 | 1.818 | 138 | 0.071 | -0.003 | 0.073 |
| ω _2 (60:40)_ | 0.051 | 0.050 | 1.017 | 138 | 0.311 | -0.048 | 0.150 |
| ω _2 (90:10)_ | -0.297 | 0.418 | -0.711 | 138 | 0.479 | -1.124 | 0.530 |
| Adj. R2 = 0.0014 | |  |  |  |  |  |  |
|  | |  |  |  |  |  |  |
|  | |  |  |  |  |  |  |
