# Supplementary material for: The effects of base rate neglect on sequential belief updating and real-world beliefs
Source: PLoS Comput Biol. 2022 Dec 22;18(12):e1010796. doi: 10.1371/journal.pcbi.1010796 (PMC9831339; doi:10.1371/journal.pcbi.1010796)
Supplement: S13 Table — (DOCX) [file pcbi.1010796.s013.docx]

**Table S13. Linear model predicting participant scores on their Social Withdrawal Factor Score (S3 Fig) based on their fitted parameters from the weighted Bayesian model (N = 143).** The factor score is not associated with any model parameters. Therefore, variation in the Social Withdrawal factor does not appear to be specifically driving interindividual differences in $\omega_{1}$. Wilkinson Notation: Social Withdrawal Factor Score ~ $\omega_{1}$ + $\omega_{2_{(51:49)}}$+ $\omega_{2_{(60:40)}}$ + $\omega_{2_{(90:10)}}$.

| **Effect** | **Estimate** | ***SE*** | ***t-stat*** | **df** | ***p*** | **95% CI** | |
| --- | --- | --- | --- | --- | --- | --- | --- |
|  |  |  |  |  |  | ***LL*** | ***UL*** |
| Intercept | 0.278 | 0.762 | 0.364 | 138 | 0.716 | -1.229 | 1.785 |
| ω_1_ | 0.689 | 0.704 | 0.978 | 138 | 0.330 | -0.704 | 2.081 |
| ω _2 (51:49)_ | 0.001 | 0.024 | 0.041 | 138 | 0.967 | -0.047 | 0.049 |
| ω _2 (60:40)_ | -0.080 | 0.063 | -1.263 | 138 | 0.209 | -0.206 | 0.045 |
| ω _2 (90:10)_ | 0.284 | 0.528 | 0.537 | 138 | 0.592 | -0.760 | 1.328 |
| Adj. R2 = -0.0087 | |  |  |  |  |  |  |
