# Supplementary material for: The effects of base rate neglect on sequential belief updating and real-world beliefs
Source: PLoS Comput Biol. 2022 Dec 22;18(12):e1010796. doi: 10.1371/journal.pcbi.1010796 (PMC9831339; doi:10.1371/journal.pcbi.1010796)
Supplement: S14 Table — (DOCX) [file pcbi.1010796.s014.docx]

**S14 Table. Linear mixed-effects model predicting probability estimates based on bead draw and bead ratio for the main sample in study 2 (N = 91).** This is comparable to the analysis illustrated in Fig 3a, but for study 2.

Wilkinson Notation: Estimates ~ Draw*Ratio +(Draw*Ratio|Subject_Number).

| **Effect** | **Estimate** | ***SE*** | ***t-stat*** | **df** | ***p*** | **95% CI** | |
| --- | --- | --- | --- | --- | --- | --- | --- |
|  |  |  |  |  |  | ***LL*** | ***UL*** |
| Intercept | 0.438 | 0.008 | 53.315 | 134.00 | 4.218e-92 | 0.422 | 0.454 |
| Bead Draw | 0.087 | 0.013 | 6.474 | 106.07 | 3.031e-09 | 0.061 | 0.114 |
| Bead Ratio | -0.010 | 0.003 | -3.299 | 93.34 | 0.001 | -0.016 | -0.004 |
| Bead Draw * Bead Ratio | 0.067 | 0.004 | 17.354 | 93.26 | 5.898e-31 | 0.060 | 0.075 |
| Adj. R2 = 0.4129 |  |  |  |  |  |  |  |
