# Supplementary material for: The effects of base rate neglect on sequential belief updating and real-world beliefs
Source: PLoS Comput Biol. 2022 Dec 22;18(12):e1010796. doi: 10.1371/journal.pcbi.1010796 (PMC9831339; doi:10.1371/journal.pcbi.1010796)
Supplement: S15 Table — (DOCX) [file pcbi.1010796.s015.docx]

**S15 Table. Linear mixed-effects model predicting probability estimates based on bead draw and bead ratio for the main sample in study 2 (N = 91) for matched trials.** This analysis only includes the 16 trials that are matched between the 60:40 and 90:10 bead ratio conditions. It is comparable to the analysis illustrated in the Fig 3a inset, but for study 2.

Wilkinson Notation: Estimates ~ Draw*Ratio +(Draw*Ratio|Subject_Number).

| **Effect** | **Estimate** | ***SE*** | ***t-stat*** | **df** | ***p*** | **95% CI** | |
| --- | --- | --- | --- | --- | --- | --- | --- |
|  |  |  |  |  |  | ***LL*** | ***UL*** |
| Intercept | 0.477 | 0.010 | 48.522 | 337.01 | 4.155e-154 | 0.458 | 0.496 |
| Bead Draw | 0.045 | 0.014 | 3.302 | 189.14 | 0.001 | 0.018 | 0.072 |
| Bead Ratio | 0.018 | 0.004 | 5.028 | 96.73 | 2.273e-06 | 0.011 | 0.025 |
| Bead Draw * Bead Ratio | 0.034 | 0.004 | 8.421 | 101.68 | 2.530e-13 | 0.026 | 0.042 |
| Adj. R2 = 0.4354 |  |  |  |  |  |  |  |
