# Supplementary material for: The effects of base rate neglect on sequential belief updating and real-world beliefs
Source: PLoS Comput Biol. 2022 Dec 22;18(12):e1010796. doi: 10.1371/journal.pcbi.1010796 (PMC9831339; doi:10.1371/journal.pcbi.1010796)
Supplement: S16 Table — (DOCX) [file pcbi.1010796.s016.docx]

**S16 Table. Linear mixed-effects model predicting final estimate difference based on evidence asymmetry and bead ratio for the main sample in study 2 (N = 91).** This is comparable to the analysis illustrated in Fig 3b, but for the main sample in study 2

Wilkinson Notation: Final Estimate Difference ~ Ratio* Evidence Asymmetry + (Ratio*Evidence Asymmetry|Subject_Number).

| **Effect** | **Estimate** | ***SE*** | ***t-stat*** | **df** | ***p*** | **95% CI** | |
| --- | --- | --- | --- | --- | --- | --- | --- |
|  |  |  |  |  |  | ***LL*** | ***UL*** |
| Intercept | 0.029 | 0.025 | 1.177 | 432.23 | 0.240 | -0.020 | 0.078 |
| Evidence Asymmetry | -0.010 | 0.007 | -1.545 | 86.45 | 0.126 | -0.023 | 0.003 |
| Bead Ratio | -0.001 | 3.433e-04 | -1.708 | 465.50 | 0.088 | -0.001 | 8.832e-05 |
| Evidence Asymmetry * Bead Ratio | 3.049e-04 | 1.082e-04 | 2.819 | 77.99 | 0.006 | 8.954e-05 | 0.001 |
| Adj. R2 = 0.2638 |  |  |  |  |  |  |  |
