# Supplementary material for: The effects of base rate neglect on sequential belief updating and real-world beliefs
Source: PLoS Comput Biol. 2022 Dec 22;18(12):e1010796. doi: 10.1371/journal.pcbi.1010796 (PMC9831339; doi:10.1371/journal.pcbi.1010796)
Supplement: S17 Table — (DOCX) [file pcbi.1010796.s017.docx]

**S17 Table. Linear mixed-effects model predicting mean logit-belief updates based on mean logit-priors and bead ratio**

**for the main sample in study 2 (N = 91).** This analysis corresponds to Fig S4.

Wilkinson Notation: Logit Belief Update ~ Logit Prior*Ratio +(Logit Prior*Ratio|Subject_Number).

| **Effect** | **Estimate** | ***SE*** | ***t-stat*** | **df** | ***p*** | **95% CI** | |
| --- | --- | --- | --- | --- | --- | --- | --- |
|  |  |  |  |  |  | ***LL*** | ***UL*** |
| Intercept | 0.241 | 0.019 | 12.726 | 89.88 | 8.115e-22 | 0.203 | 0.279 |
| Logit-Prior | -0.077 | 0.028 | -2.783 | 95.74 | 0.006 | -0.132 | -0.022 |
| Bead Ratio | 0.214 | 0.020 | 10.841 | 84.79 | 1.091e-17 | 0.175 | 0.253 |
| Logit-Prior * Bead Ratio | 0.001 | 0.009 | 0.152 | 89.80 | 0.879 | -0.016 | 0.019 |
| Adj. R2 = 0.3644 |  |  |  |  |  |  |  |
