# Supplementary material for: The effects of base rate neglect on sequential belief updating and real-world beliefs
Source: PLoS Comput Biol. 2022 Dec 22;18(12):e1010796. doi: 10.1371/journal.pcbi.1010796 (PMC9831339; doi:10.1371/journal.pcbi.1010796)
Supplement: S18 Table — (DOCX) [file pcbi.1010796.s018.docx]

**S18 Table. Linear mixed-effects model predicting final estimate difference based on evidence asymmetry and bead ratio** **for the low PDI group only (N = 57).** This analysis corresponds to Fig 4a in the main text.

Wilkinson Notation: Final Estimate Difference ~ Ratio* Evidence Asymmetry + (Ratio*Evidence Asymmetry|Subject_Number).

| **Effect** | **Estimate** | ***SE*** | ***t-stat*** | **df** | ***p*** | **95% CI** | |
| --- | --- | --- | --- | --- | --- | --- | --- |
|  |  |  |  |  |  | ***LL*** | ***UL*** |
| Intercept | 0.011 | 0.029 | 0.381 | 116.95 | 0.704 | -0.046 | 0.067 |
| Evidence Asymmetry | -0.008 | 0.007 | -1.128 | 90.73 | 0.262 | -0.023 | 0.006 |
| Bead Ratio | -3.315e-04 | 3.951e-04 | -0.839 | 148.63 | 0.403 | -0.001 | 4.492e-04 |
| Evidence Asymmetry * Bead Ratio | 2.647e-04 | 1.226e-04 | 2.159 | 70.08 | 0.034 | 2.019e-05 | 0.001 |
| Adj. R2 = 0.2526 |  |  |  |  |  |  |  |
