# Supplementary material for: The effects of base rate neglect on sequential belief updating and real-world beliefs
Source: PLoS Comput Biol. 2022 Dec 22;18(12):e1010796. doi: 10.1371/journal.pcbi.1010796 (PMC9831339; doi:10.1371/journal.pcbi.1010796)
Supplement: S19 Table — (DOCX) [file pcbi.1010796.s019.docx]

**S19 Table. Linear mixed-effects model predicting final estimate difference based on evidence asymmetry and bead ratio for the high PDI group only (N = 34).** This analysis corresponds to Fig 4b in the main text.

Wilkinson Notation: Final Estimate Difference ~ Ratio* Evidence Asymmetry +(Ratio*Evidence Asymmetry|Subject_Number).

| **Effect** | **Estimate** | ***SE*** | ***t-stat*** | **df** | ***p*** | **95% CI** | |
| --- | --- | --- | --- | --- | --- | --- | --- |
|  |  |  |  |  |  | ***LL*** | ***UL*** |
| Intercept | 0.062 | 0.047 | 1.309 | 185.47 | 0.192 | -0.031 | 0.155 |
| Evidence Asymmetry | -0.013 | 0.013 | -1.018 | 29.08 | 0.317 | -0.039 | 0.013 |
| Bead Ratio | -0.001 | 6.484e-04 | -1.601 | 205.85 | 0.111 | -0.002 | 2.405e-04 |
| Evidence Asymmetry * Bead Ratio | 3.712e-04 | 2.068e-04 | 1.795 | 28.61 | 0.083 | -5.210e-05 | 0.001 |
| Adj. R2 = 0.2791 |  |  |  |  |  |  |  |
