# Supplementary material for: The effects of base rate neglect on sequential belief updating and real-world beliefs
Source: PLoS Comput Biol. 2022 Dec 22;18(12):e1010796. doi: 10.1371/journal.pcbi.1010796 (PMC9831339; doi:10.1371/journal.pcbi.1010796)
Supplement: S20 Table — (DOCX) [file pcbi.1010796.s020.docx]

**S20 Table. Linear mixed-effects model predicting mean logit-belief updates based on mean logit-priors and bead ratio for the low PDI group only (N = 57).** This analysis corresponds to Fig 4c in the main text.

Wilkinson Notation: Logit Belief Update ~ Logit Prior*Ratio +(Logit Prior*Ratio|Subject_Number).

| **Effect** | **Estimate** | ***SE*** | ***t-stat*** | **df** | ***p*** | **95% CI** | |
| --- | --- | --- | --- | --- | --- | --- | --- |
|  |  |  |  |  |  | ***LL*** | ***UL*** |
| Intercept | 0.206 | 0.018 | 11.196 | 55.99 | 6.526e-16 | 0.169 | 0.243 |
| Logit-Prior | -0.039 | 0.028 | -1.383 | 59.67 | 0.172 | -0.096 | 0.018 |
| Bead Ratio | 0.223 | 0.026 | 8.477 | 52.76 | 2.021e-11 | 0.170 | 0.276 |
| Logit-Prior * Bead Ratio | 6.244e-06 | 0.012 | 0.001 | 54.98 | 1.000 | -0.023 | 0.023 |
| Adj. R2 = 0.3319 |  |  |  |  |  |  |  |
