# Supplementary material for: The effects of base rate neglect on sequential belief updating and real-world beliefs
Source: PLoS Comput Biol. 2022 Dec 22;18(12):e1010796. doi: 10.1371/journal.pcbi.1010796 (PMC9831339; doi:10.1371/journal.pcbi.1010796)
Supplement: S21 Table — (DOCX) [file pcbi.1010796.s021.docx]

**S21 Table. Linear mixed-effects model predicting mean logit-belief updates based on mean logit-priors and bead ratio for the high PDI group only (N = 34).** This analysis corresponds to Fig 4d in the main text.

Wilkinson Notation: Logit Belief Update ~ Logit Prior*Ratio +(Logit Prior*Ratio|Subject_Number).

| **Effect** | **Estimate** | ***SE*** | ***t-stat*** | **df** | ***p*** | **95% CI** | |
| --- | --- | --- | --- | --- | --- | --- | --- |
|  |  |  |  |  |  | ***LL*** | ***UL*** |
| Intercept | 0.299 | 0.038 | 7.814 | 33.72 | 4.539e-09 | 0.221 | 0.376 |
| Logit-Prior | -0.142 | 0.054 | -2.602 | 35.31 | 0.013 | -0.252 | -0.031 |
| Bead Ratio | 0.202 | 0.031 | 6.603 | 33.88 | 1.459e-07 | 0.140 | 0.264 |
| Logit-Prior * Bead Ratio | 0.007 | 0.014 | 0.471 | 34.64 | 0.640 | -0.022 | 0.036 |
| Adj. R2 = 0.3888 |  |  |  |  |  |  |  |
