# Supplementary material for: The effects of base rate neglect on sequential belief updating and real-world beliefs
Source: PLoS Comput Biol. 2022 Dec 22;18(12):e1010796. doi: 10.1371/journal.pcbi.1010796 (PMC9831339; doi:10.1371/journal.pcbi.1010796)
Supplement: S22 Table — (DOCX) [file pcbi.1010796.s022.docx]

**S22 Table. Statistics for rank-sum tests of group differences between Low (N = 34) and High (N =. 57) PDI groups for belief updating measures yielded by study 2**. This table corresponds to Fig 5a in the main text. Cliff’s delta was calculated using the effect-size toolbox[1].

| **Metric** | **Rank Sum** | **Z** | ***p*** | **Cliff's Delta (δ)** | **Cliff’s Delta 95% CI** | |
| --- | --- | --- | --- | --- | --- | --- |
|  |  |  |  |  | ***LL*** | ***UL*** |
| Evidence Asymmetry | 2537 | -0.693 | 0.488 | 0.088 | 0.358 | -0.179 |
| Final Estimate Difference | 2547 | -0.611 | 0.541 | 0.077 | 0.347 | -0.190 |
| Prior Dependent Updating Slope | 2835 | 1.743 | 0.081 | -0.220 | 0.011 | -0.489 |
| ω_1_ | 2910 | 2.359 | 0.018 | -0.297 | -0.066 | -0.585 |
| ω _2 (51:49)_ | 2525 | -0.792 | 0.429 | 0.100 | 0.370 | -0.163 |
| ω _2 (60:40)_ | 2419 | -1.661 | 0.097 | 0.209 | 0.480 | -0.052 |
| ω _2 (90:10)_ | 2511 | -0.907 | 0.365 | 0.115 | 0.367 | -0.137 |

References

1. Hentschke H, Stüttgen MC. Computation of measures of effect size for neuroscience data sets. Eur J Neurosci. 2011;34: 1887–1894. doi:10.1111/j.1460-9568.2011.07902.x
