# Supplementary material for: The effects of base rate neglect on sequential belief updating and real-world beliefs
Source: PLoS Comput Biol. 2022 Dec 22;18(12):e1010796. doi: 10.1371/journal.pcbi.1010796 (PMC9831339; doi:10.1371/journal.pcbi.1010796)
Supplement: S23 Table — (DOCX) [file pcbi.1010796.s023.docx]

**S23 Tavle. Pair-wise correlations for study 2 between mean PDI score (mean of prescreening and experimental session PDI scores; see Methods), paranoia checklist score, the final estimate difference, the evidence asymmetry slope, the prior dependent updating slope, and** $\boldsymbol{\omega}_{\boldsymbol{1}}$**.** The prior dependent updating bias was defined as the logit-prior beta values for individual-level linear mixed-effects analysis consistent with S17 Table, S20 Table, and S21 Table but conducted for each participant individually. Consistent with earlier analyses, the most extreme prior dependent updating bias are excluded. Partial correlations control for the three $\omega_{2_{(q)}}$ parameters, and the model root-mean-squared-error and are only reported for correlations that involve relevant model parameters such as $\omega_{1}$. Sample size is consistent with group vs dimensional analyses as detailed in the main text and the methods.

| **Correlation Table** | | | | | | | | | | |
| --- | --- | --- | --- | --- | --- | --- | --- | --- | --- | --- |
| Correlations between indices of base-rate neglect (n = 91) | | | | | **Spearman** | | | **Partial Spearman** | | |
|  |  |  |  |  | **rho** | | **p** | **rho** | | **p** |
| Final Estimate Difference | | - | Evidence Asymmetry Slope | | 0.536 | *** | 7.1310e-08 | N/A |  | N/A |
| Final Estimate Difference | | - | Prior Dependent Updating Slope | | -0.245 | * | 0.019 | N/A |  | N/A |
| Final Estimate Difference | | - | $\omega_{1}$ | | -0.550 | *** | 2.8407e-08 | -0.565 | *** | 1.2346e-08 |
| Evidence Asymmetry Slope | | - | Prior Dependent Updating Slope | | -0.251 | ** | 0.017 | N/A |  | N/A |
| Evidence Asymmetry Slope | | - | $\omega_{1}$ | | -0.446 | *** | 1.1852e-05 | -0.408 | *** | 8.8797e-05 |
| Prior Dependent Updating Slope | | - | $\omega_{1}$ | | 0.534 | *** | 8.1704e-08 | 0.437 | *** | 2.2684e-05 |
|  | | | | | | | | | | |
| Correlations between indices of base-rate neglect and PDI (n = 116) | | | | | **Spearman** | | | **Partial Spearman** | | |
|  |  |  |  |  | **rho** | | **p** | **rho** | | **p** |
| mean PDI | | - | | $\omega_{1}$ | -0.249 | ** | 0.007 | -0.219 | * | 0.0206 |
| mean PDI | | - | | Prior Dependent Updating Slope | -0.172 |  | 0.0654 | N/A |  | N/A |
| mean PDI | | - | | Final Estimate Difference | 0.084 |  | 0.3727 | N/A |  | N/A |
| mean PDI | | - | | Evidence Asymmetry Slope | 0.115 |  | 0.2187 | N/A |  | N/A |
|  | | | | | | | | | | |
| Correlations between indices of base-rate neglect and Paranoia Checklist (n = 116) | | | | | **Spearman** | | | **Partial Spearman** | | |
|  |  |  |  |  | **rho** | | **p** | **rho** | | **p** |
| Paranoia Checklist | - | | | $\omega_{1}$ | -0.227 | * | 0.0144 | -0.232 | * | 0.0140 |
| Paranoia Checklist | - | | | Prior Dependent Updating Slope | -0.192 | * | 0.0394 | N/A |  | N/A |
| Paranoia Checklist | - | | | Final Estimate Difference | 0.117 |  | 0.2095 | N/A |  | N/A |
| Paranoia Checklist | - | | | Evidence Asymmetry Slope | 0.161 |  | 0.0836 | N/A |  | N/A |
|  | | | | | | | | | | |
| p < 0.05, ** p < 0.01, *** p < 0.001 | | | | | | | | | | |
