# Supplementary material for: The effects of base rate neglect on sequential belief updating and real-world beliefs
Source: PLoS Comput Biol. 2022 Dec 22;18(12):e1010796. doi: 10.1371/journal.pcbi.1010796 (PMC9831339; doi:10.1371/journal.pcbi.1010796)
Supplement: S24 Table — (DOCX) [file pcbi.1010796.s024.docx]

**S24 Table. Correlations between measures of response variability and** $\boldsymbol{\omega}_{\boldsymbol{1}}$ **by individual study and for the full sample.** Partial correlations control for the model goodness-of-fit. PDI and paranoia checklist correlations with $\sigma_{prior}^{2}$ and response variance are also reported for the data from study 2. Partial correlations control for all three $\sigma_{Likelihood}^{2}$ parameters and the noisy-sampling model root-mean-squared-error. Study 3 reflects the combined data from studies 1 and 2

| **Correlation Table** | | | | | | | | |
| --- | --- | --- | --- | --- | --- | --- | --- | --- |
| Study 1 (n = 151) | | | **Spearman** | | | **Partial Spearman** | | |
|  |  |  | **rho** | | **p** | **rho** | | **p** |
| RMSE (Weighted Bayesian Model) | - | $\omega_{1}$ | -0.392 | *** | 7.9819e-07 | N/A |  | N/A |
| RMSE (Weighted Bayesian Model) | - | σ^2^_prior_ | 0.282 | *** | 4.6573e-04 | N/A |  | N/A |
| Response Variance | - | $\omega_{1}$ | -0.446 | *** | 1.4128e-08 | N/A |  | N/A |
| Response Variance | - | σ^2^_prior_ | 0.344 | *** | 1.7227e-05 | N/A |  | N/A |
|  | | | | | | | | |
| Study 2 (n = 116) | | | **Spearman** | | | **Partial Spearman** | | |
|  |  |  | **rho** | | **p** | **rho** | | **p** |
| RMSE (Weighted Bayesian Model) | - | $\omega_{1}$ | -0.411 | *** | 5.4907e-06 | N/A |  | N/A |
| RMSE (Weighted Bayesian Model) | - | σ^2^_prior_ | 0.225 | ** | 0.020 | N/A |  | N/A |
| Response Variance | - | $\omega_{1}$ | -0.442 | *** | 8.7839e-07 | N/A |  | N/A |
| Response Variance | - | σ^2^_prior_ | 0.329 | *** | 3.3670e-04 | N/A |  | N/A |
| mean PDI | - | σ^2^_prior_ | 0.291 | ** | 0.002 | 0.278 | ** | 0.003 |
| mean PDI |  | Response Variance | 0.142 |  | 0.128 | N/A |  | N/A |
| Paranoia | - | σ^2^_prior_ | 0.224 | * | 0.016 | 0.237 | * | 0.012 |
| Paranoia |  | Response Variance | 0.142 |  | 0.128 | N/A |  | N/A |
|  | | | | | | | | |
| Study 3 (n = 267) | | | **Spearman** | | | **Partial Spearman** | | |
|  |  |  | **rho** | | **p** | **rho** | | **p** |
| RMSE (Weighted Bayesian Model) | - | $\omega_{1}$ | -0.404 | *** | 8.9331e-12 | N/A |  | N/A |
| RMSE (Weighted Bayesian Model) | - | σ^2^_prior_ | 0.257 | *** | 2.3260e-05 | N/A |  | N/A |
| Response Variance | - | $\omega_{1}$ | -0.457 | *** | 2.2251e-308 | N/A |  | N/A |
| Response Variance | - | σ^2^_prior_ | 0.350 | *** | 5.0579e-09 | N/A |  | N/A |
|  | | | | | | | | |
| * p < .05, ** p < .01, *** p < .001 | | | | | | | | |
