# Supplementary material for: The effects of base rate neglect on sequential belief updating and real-world beliefs
Source: PLoS Comput Biol. 2022 Dec 22;18(12):e1010796. doi: 10.1371/journal.pcbi.1010796 (PMC9831339; doi:10.1371/journal.pcbi.1010796)
Supplement: S25 Table — (DOCX) [file pcbi.1010796.s025.docx]

**S25 Table. Descriptive statistics for the parameters of the noisy sampling model for study 3.**

| Study 3 (n = 267) | Mean | Standard Deviation | Median | 25th Percentile | 75th percentile |
| --- | --- | --- | --- | --- | --- |
| σ^2^_prior_ | 0.165 | 1.144 | 6.183e-04 | 1.590e-07 | 0.015 |
| σ^2^_51:49_ | 0.500 | 2.440 | 9.745e-06 | 1.387e-06 | 1.225e-04 |
| σ^2^_60:40_ | 0.110 | 0.179 | 4.040e-02 | 4.678e-06 | 0.137 |
| σ^2^_90:10_ | 1.864 | 1.411 | 1.645 | 0.957 | 2.490 |
