# Supplementary material for: The effects of base rate neglect on sequential belief updating and real-world beliefs
Source: PLoS Comput Biol. 2022 Dec 22;18(12):e1010796. doi: 10.1371/journal.pcbi.1010796 (PMC9831339; doi:10.1371/journal.pcbi.1010796)
Supplement: S26 Table — (DOCX) [file pcbi.1010796.s026.docx]

**S26 Table. Statistics for rank sum tests for group differences between Low (N = 34) and High (N =57) PDI groups for belief updating measures yielded by study 3.** This table corresponds to Fig 7a in the main text.

| **Metric** | **Rank Sum** | **Z** | **p** | **Cliff's Delta (δ)** | **Cliff’s Delta 95% CI** | |
| --- | --- | --- | --- | --- | --- | --- |
|  |  |  |  |  | **LL** | **UL** |
| Response Variance | 2430 | -1.571 | 0.116 | -0.198 | -0.464 | 0.063 |
| $\sigma_{Prior}^{2}$ | 2294 | -2.687 | 0.007 | -0.338 | -0.591 | -0.119 |
| $\sigma_{51:49}^{2} Likelihood$ | 2571 | -0.414 | 0.679 | -0.053 | -0.299 | 0.198 |
| $\sigma_{60:40}^{2} Likelihood$ | 2801 | 1.464 | 0.143 | 0.185 | -0.065 | 0.480 |
| $\sigma_{90:10}^{2} Likelihood$ | 2726 | 0.849 | 0.396 | 0.107 | -0.131 | 0.374 |
