# Supplementary material for: The effects of base rate neglect on sequential belief updating and real-world beliefs
Source: PLoS Comput Biol. 2022 Dec 22;18(12):e1010796. doi: 10.1371/journal.pcbi.1010796 (PMC9831339; doi:10.1371/journal.pcbi.1010796)
Supplement: S27 Table — (DOCX) [file pcbi.1010796.s027.docx]

**S27 Table.** $\boldsymbol{\omega}_{\boldsymbol{1}}$ **is not associated with response times.** The noisy sampling model suggests that prior weighting (indexed by $\omega_{1}$) is a rational response to prior noise. As it is commonly assumed in inferential models[1,2], increased response times would increase noise in the prior representation, which according to the noisy sampling model would lead to lower $\omega_{1}$ (i.e., increased base-rate neglect). If $\omega_{1}$ reflects a state-like phenomenon and therefore could be influenced by factors such as inattention, we would expect to see an effect of $\omega_{1}$or an interaction between $\omega_{1}$ and estimate certainty on response times, since $\omega_{1}$ should be dependent on the response times. Alternatively, if $\omega_{1}$ represents a trait-like phenomenon reflecting the individual’s intrinsic prior noise, we would expect no main effect or interaction with $\omega_{1}$ on response time, since the only relevant factor determining RT should be the resulting estimate certainty at a given draw. To statistically evaluate this relationship, we conducted a linear mixed-effects regression predicting trial-by-trial response times on based on base-rate neglect ($\omega_{1}$), absolute posterior certainty, the interaction between $\omega_{1}$ and absolute posterior certainty, and the absolute difference between the posterior estimate and the random starting point for the slider (to account for longer response times driven by the cursor simply starting further away from the final estimate). This regression included data for all 267 participants, as in study 3. There was no main effect of, or interaction with $\omega_{1}$, on response times. Therefore, these results are more consistent with $\boldsymbol{\omega}_{\boldsymbol{1}}$ as a trait-like phenomenon.

| Real Subjective Estimates |  |  |  |  |  |  |  |
| --- | --- | --- | --- | --- | --- | --- | --- |
| **Effect** | **Estimate** | **SE** | **t-stat** | **df** | **p** | **95% CI** | |
|  |  |  |  |  |  | **LL** | **UL** |
| Intercept | 2.115 | 0.210 | 10.053 | 115881 | 0.000 | 1.702 | 2.527 |
| $\omega_{1}$ | 0.361 | 0.217 | 1.665 | 115881 | 0.096 | -0.064 | 0.786 |
| Absolute Cursor Difference | 0.579 | 0.024 | 24.562 | 115881 | 0.000 | 0.533 | 0.625 |
| Absolute Posterior Estimate | -1.354 | 0.285 | -4.755 | 115881 | 0.000 | -1.912 | -0.796 |
| $\omega_{1}$: Absolute Posterior Estimate | 0.164 | 0.299 | 0.548 | 115881 | 0.584 | -0.422 | 0.750 |
| Adj. R2 = 0.3749 |  |  |  |  |  |  |  |

References

1. Haefner RM, Berkes P, Fiser J. Perceptual Decision-Making as Probabilistic Inference by Neural Sampling. Neuron. 2016;90: 649–660. doi:10.1016/j.neuron.2016.03.020

2. Gold JI, Shadlen MN. The Neural Basis of Decision Making. Annual Review of Neuroscience. 2007;30: 535–574. doi:10.1146/annurev.neuro.29.051605.113038
