# Supplementary material for: The effects of base rate neglect on sequential belief updating and real-world beliefs
Source: PLoS Comput Biol. 2022 Dec 22;18(12):e1010796. doi: 10.1371/journal.pcbi.1010796 (PMC9831339; doi:10.1371/journal.pcbi.1010796)
Supplement: S28 Table — (DOCX) [file pcbi.1010796.s028.docx]

**S28 Table. Weighted Bayesian Belief Updating Models**

| **Model Name** | **# of Free Parameters** | **Equations Fitted** | **Search Constraints** |
| --- | --- | --- | --- |
| $0 \omega_{1},0 \omega_{2}$ | 0 | $Posterior=Prior+ LLR$ | - |
| $1 \omega_{1},0 \omega_{2}$ | 1 | $Posterior=\omega_{1}Prior+ LLR$ | $0<\omega_{1}<20$ |
| $0 \omega_{1},1 \omega_{2}$ | 1 | $Posterior=Prior+ \omega_{2}LLR$ | $0<\omega_{2}<20$ |
| $1 \omega_{1},1 \omega_{2}$ | 2 | $Posterior=\omega_{1}Prior+ \omega_{2}LLR$ | $0<\omega_{1}<20$  $0<\omega_{2}<20$ |
| $1 \omega_{1},3 \omega_{2}$ | 5 | $Posterior=\omega_{1}Prior+ \omega_{2}(q)LLR$ | $0<\omega_{1}<20$  $0<\omega_{2}(q)<20$ |
| $3 \omega_{1},1 \omega_{2}$ | 5 | $Posterior=\omega_{1}(q)Prior+ \omega_{2}LLR$ | $0<\omega_{1}(q)<20$  $0<\omega_{2}<20$ |
| $3 \omega_{1},3 \omega_{2}$ | 8 | $Posterior=\omega_{1}(q)Prior+ \omega_{2}(q)LLR$ | $0<\omega_{1}(q)<20$  $0<\omega_{2}(q)<20$ |
| $0 \omega_{1},3 \omega_{2}, 1 \tau$ | 5 | $Posterior=(1-\tau)(Prior+ \omega_{2}(q)LLR+$  $\tau((1-Prior)+ \omega_{2}(q)(1-LLR))$ | $0<\omega_{2}(q)<20$  $0<\tau<20$ |
| $1 \omega_{1},3 \omega_{2}, 1 \tau$ | 5 | $Posterior=(1-\tau)(\omega_{1}Prior+ \omega_{2}(q)LLR)+\tau(\omega_{1}(1-Prior)+ \omega_{2}(q)(1-LLR))$ | $0<\omega_{1}<20$  $0<\omega_{2}(q)<20$  $0<\tau<20$ |
| $1 \left( \omega_{1}-\omega_{2} \right),$  $3 \omega_{2}$ | 5 | $Posterior={(\omega}_{1}-\omega_{2}(q))Prior+ \omega_{2}(q)LLR$ | $0<\omega_{1}<20$  $0<\omega_{2}(q)<20$ |
| **Heuristic Models (Fit to 60:40 and 90:10 bead ratio condition data for exclusion purposes)** | | | |
| $No Prior, 2 \omega_{2}$ | 2 | $Posterior=\omega_{2}(q)LLR$ | $0<\omega_{2}(q)<20$ |
| $No Prior, 2 \omega_{2}$ | 2 | $\left\{ \begin{aligned} if n_{Majority}>n_{Minority}, Posterior= + \omega_{2}\left( q \right) \left\vert LLR \right\vert\\ if n_{Majority}<n_{Minority},Posterior= {- \omega}_{2}\left( q \right) \left\vert LLR \right\vert\\ if n_{Majority}=n_{Minority}, Posterior=logit(.5) \end{aligned} \right.$ | $0<\omega_{2}(q)<20$ |
| $\omega_{1}(q)$ and $\omega_{2}(q)$ denote parameters that vary by bead-ratio condition (i.e., one free parameter for each of the three conditions). $n_{Majority}$ refers to the total number of beads matching the majority color of the correct box seen up until that point; $n_{Minority}$ refers to the total number of beads matching the majority color of the incorrect box seen up until that point. For these models, both *Prior* and *Posterior* reflect probabilities in logit space. | | | |
