# Supplementary material for: The effects of base rate neglect on sequential belief updating and real-world beliefs
Source: PLoS Comput Biol. 2022 Dec 22;18(12):e1010796. doi: 10.1371/journal.pcbi.1010796 (PMC9831339; doi:10.1371/journal.pcbi.1010796)
Supplement: S29 Table — (DOCX) [file pcbi.1010796.s029.docx]

**S29 Table. PDI items and order of presentation.** Participants had to report Yes/No to each item, indicating if they have had the belief or experience described by the item. If they responded No, then they moved onto the next item. If they responded Yes, they were asked to rate on a scale of 1-5 how distressing the belief or experience is, how often they think about the belief or experience, and how true they believe the belief or experience to be (3 separate ratings). Participants were explicitly told that their responses should *not* reflect experiences or beliefs they may have had under the influence of drugs. PDI Global Score is the sum of all item ratings, including 1 for yes and 0 for no. Each item has an item-score range of 0 -16. The PDI Global has a score range of 0 – 336 (21 items; maximum score of 16).

| **PDI Item Number** | **PDI Item Question** |
| --- | --- |
| 1 | Do you ever feel as if people seem to drop hints about you or say things with a double meaning? |
| 2 | Do you ever feel as if things in magazines or on TV were written especially for you? |
| 3 | Do you ever feel as if some people are not what they seem to be? |
| 4 | Do you ever feel as if you are being persecuted in some way? |
| 5 | Do you ever feel as if there is a conspiracy against you? |
| 6 | Do you ever feel as if you are, or destined to be someone very important? |
| 7 | Do you ever feel that you are a very special or unusual person? |
| 8 | Do you ever feel that you are especially close to God? |
| 9 | Do you ever think people can communicate telepathically? |
| 10 | Do you ever feel as if electrical devices such as computers can influence the way you think? |
| 11 | Do you ever feel as if you have been chosen by God in some way? |
| 12 | Do you believe in the power of witchcraft, voodoo or the occult? |
| 13 | Are you often worried that your partner may be unfaithful? |
| 14 | Do you ever feel that you have sinned more than the average person? |
| 15 | Do you ever feel that people look at you oddly because of your appearance? |
| 16 | Do you ever feel as if you had no thoughts in your head at all? |
| 17 | Do you ever feel as if the world is about to end? |
| 18 | Do your thoughts ever feel alien to you in some way? |
| 19 | Have your thoughts ever been so vivid that you were worried other people would hear them? |
| 20 | Do you ever feel as if your own thoughts were being echoed back to you? |
| 21 | Do you ever feel as if you are a robot or zombie without a will of your own? |
