# Supplementary material for: The effects of base rate neglect on sequential belief updating and real-world beliefs
Source: PLoS Comput Biol. 2022 Dec 22;18(12):e1010796. doi: 10.1371/journal.pcbi.1010796 (PMC9831339; doi:10.1371/journal.pcbi.1010796)
Supplement: S30 Table — (DOCX) [file pcbi.1010796.s030.docx]

**S30 Table. Bead sequences (i.e., trials) used in studies 1 and 2**. All sequences were presented one time for each listed bead ratio condition except for the 8-bead sequence, which was presented 7 times during the 90:10 condition (indicated by *). Mirror-opposite sequence pairs and symmetric sequences are highlighted.

| Sequence | Bead Ratio | Total Majority Beads | Evidence Asymmetry | Type of Evidence Sequence |
| --- | --- | --- | --- | --- |
| 1 1 1 1 1 0 0 0 | 60/90/51 | 5 | 7.5 | Front-Loaded |
| 0 0 0 1 1 1 1 1 | 60/90/51 | 5 | -7.5 | Back-Loaded |
| 0 1 1 1 0 1 1 0 | 60/90/51 | 5 | 0.5 | Front-Loaded |
| 0 1 1 0 1 1 1 0 | 60/90/51 | 5 | -0.5 | Back-Loaded |
| 1 1 1 1 1 1 0 0 | 60/90/51 | 6 | 6 | Front-Loaded |
| 0 0 1 1 1 1 1 1 | 60/90/51 | 6 | -6 | Back-Loaded |
| 1 1 1 0 1 1 1 0 | 60/90 | 6 | 3 | Front-Loaded |
| 0 1 1 1 0 1 1 1 | 60/90 | 6 | -3 | Back-Loaded |
| 1 0 1 1 1 1 0 1 | 60/90/51 | 6 | 0 | Symmetric |
| 1 1 1 1 1 1 1 0 | 60/90 | 7 | 3.5 | Front-Loaded |
| 0 1 1 1 1 1 1 1 | 60/90 | 7 | -3.5 | Back-Loaded |
| 1 1 1 1 1 0 1 1 | 60/90 | 7 | 1.5 | Front-Loaded |
| 1 1 0 1 1 1 1 1 | 60/90 | 7 | -1.5 | Back-Loaded |
| 1 1 1 1 0 1 1 1 | 60/90 | 7 | 0.5 | Front-Loaded |
| 1 1 1 0 1 1 1 1 | 60/90 | 7 | -0.5 | Back-Loaded |
| 1 1 1 1 1 1 1 1 | 60/90* | 8 | 0 | Symmetric |
| 1 1 1 1 0 1 0 0 | 60 | 5 | 6.5 | Front-Loaded |
| 0 0 1 0 1 1 1 1 | 60 | 5 | -6.5 | Back-Loaded |
| 1 1 0 1 1 1 0 0 | 60 | 5 | 4.5 | Front-Loaded |
| 0 0 1 1 1 0 1 1 | 60 | 5 | -4.5 | Back-Loaded |
| 1 1 1 1 1 0 1 0 | 60 | 6 | 5 | Front-Loaded |
| 0 1 0 1 1 1 1 1 | 60 | 6 | -5 | Back-Loaded |
| 1 0 1 0 1 0 1 0 | 51 | 4 | 2 | Front-Loaded |
| 0 1 0 1 0 1 0 1 | 51 | 4 | -2 | Back-Loaded |
| 0 1 0 1 1 0 1 0 | 51 | 4 | 0 | Symmetric |
| 1 0 1 0 0 1 0 1 | 51 | 4 | 0 | Symmetric |
