# Supplementary material for: The effects of base rate neglect on sequential belief updating and real-world beliefs
Source: PLoS Comput Biol. 2022 Dec 22;18(12):e1010796. doi: 10.1371/journal.pcbi.1010796 (PMC9831339; doi:10.1371/journal.pcbi.1010796)
Supplement: S31 Table — (DOCX) [file pcbi.1010796.s031.docx]

**S31 Table. Questions and possible responses during the miscomprehension quiz.** Bolded and italicized text indicates the correct answer. Questions were presented in this order for the first attempt, and randomized for any subsequent attempts.

|  | Question | Answer 1 | Answer 2 | Answer 3 | Answer 4 | Answer 5 |
| --- | --- | --- | --- | --- | --- | --- |
| 1 | What is your goal during this game? | ***To make as much money as possible.*** | To finish as quickly as possible. | To do math. | I do not know. |  |
| 2 | How many responses will affect the amount of money you get? | Every response. | ***One response.*** | A few responses. | I do not know. |  |
| 3 | Being inaccurate makes it more likely you will lose $10. | ***True.*** | False. | I do not know. |  |  |
| 4 | What is the most amount of money you can get? | $10 | $20 | ***$30*** | I do not know. |  |
| 5 | You must play games for least 45 minutes to get money. | ***True.*** | False. | I do not know. |  |  |
| 6 | In each game, where are the beads coming from? | Both boxes at the same time. | One box at a time, but the box can change. | ***Only one box during a single game.*** | I do not know. |  |
| 7 | What are the elements of a single game? | 1 estimate, 9 box choices | ***9 estimates, 1 box choice*** | 5 estimates, 5 box choices | I do not know. |  |
| 8 | What might affect the probability of losing $10? | Accuracy for ALL estimates. | Accuracy for ALL box decisions. | ***Accuracy for ONE estimate OR box decision.*** | Accuracy for ONE estimate AND box decision. | I do not know. |
| 9 | Being accurate makes it less likely you will lose $10. | ***True.*** | False. | I do not know. |  |  |
| 10 | During a game, the hidden box might change. | True. | ***False.*** | I do not know. |  |  |
| 11 | During a single game, how many beads will be taken out of the hidden box? | ***8*** | 5 | As many as I want to see. | I do not know. |  |
| 12 | What happens if you finish all of the games early? | I am done and can collect the money. | ***I will have to play extra games until 45 minutes have passed.*** | I do not know. |  |  |
