# Supplementary material for: The effects of base rate neglect on sequential belief updating and real-world beliefs
Source: PLoS Comput Biol. 2022 Dec 22;18(12):e1010796. doi: 10.1371/journal.pcbi.1010796 (PMC9831339; doi:10.1371/journal.pcbi.1010796)
Supplement: S1 Fig — (DOCX) [file pcbi.1010796.s032.docx]

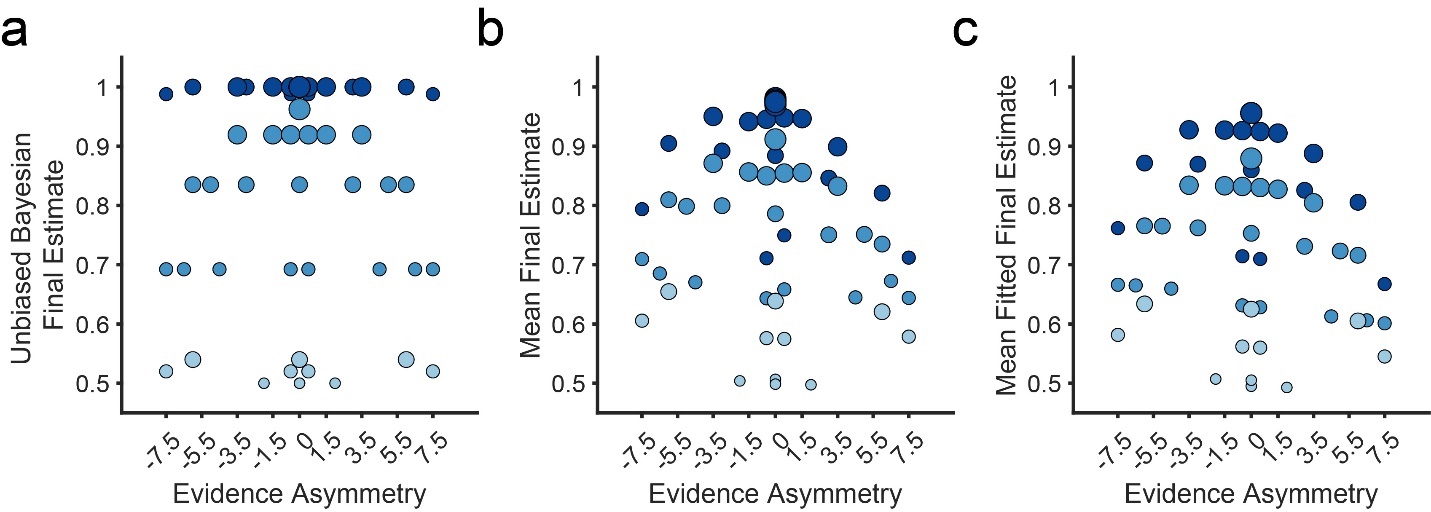


**S1 Fig. Evidence asymmetry is unrelated to mean final estimates. (a)** Evidence asymmetry is unrelated to (and therefore unlikely to be confounded with) the ideal Bayesian observer’s final estimate (objective probability) as multiple sequences with varying levels of overall evidence have an evidence asymmetry value of 0. **(b, c)** To confirm that there was no confound in the actual data accounting for the possibility that the participants might have behaved differently from predictions, we also looked at the mean final estimate across participants and the fitted mean final estimate using the weighted Bayesian model across participants. Again, there were no relationships with evidence asymmetry and mean final estimates as reported by the participants (ρ = -0.10, p = 0.46, Spearman correlation across all data points) or fitted by the model (ρ = -0.14, p = 0.31). **(b)** N = 267, using the sample from study 3. **(c)** Fitted estimates based on the best-fitting parameters for each participant from the sample from study 3. **(a, b, c)** Each dot reflects a different unique sequence used in the task, showing mean final estimate on the y-axis (with bead ratio condition shown in a different shade of blue) and an evidence-order metric (evidence asymmetry) on the x-axis, with negative values indicating front-loading of majority beads (more majority beads in the first half of the 8-bead sequence) and positive values indicating back-loading (more majority beads in the second half). Larger circles reflect sequences with more majority beads, as in Fig 1b.
