# Supplementary material for: The effects of base rate neglect on sequential belief updating and real-world beliefs
Source: PLoS Comput Biol. 2022 Dec 22;18(12):e1010796. doi: 10.1371/journal.pcbi.1010796 (PMC9831339; doi:10.1371/journal.pcbi.1010796)
Supplement: S2 Fig — (DOCX) [file pcbi.1010796.s033.docx]

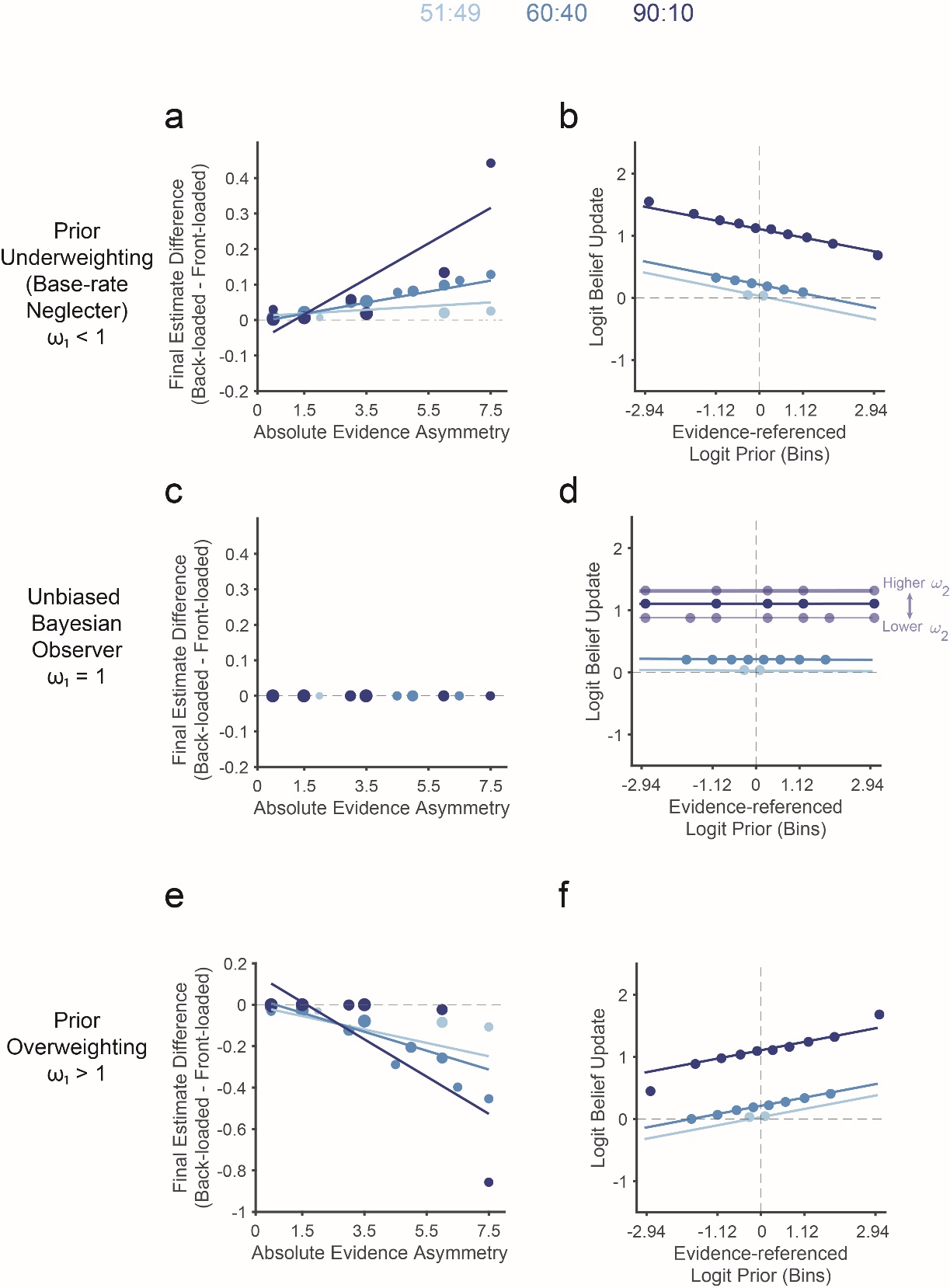


**S2 Fig.** **Condition-wise simulations of the** **final estimate difference and prior-dependent updating as a function of** $\boldsymbol{\omega}_{\boldsymbol{1}}$ **and** $\boldsymbol{\omega}_{\boldsymbol{2}}$**. (a, c, e)** Simulation of the final estimate difference, including all three bead ratio conditions (51:49, 60:40, 90:10) for a **(a)** base-rate neglecting agent ($\omega_{1}$ = 0.87, consistent with the base-rate neglecting agent simulated in Fig 2), **(c)** an unbiased Bayesian observer ($\omega_{1}$ = 1.0; all data points fall on the 0 line on the y-axis, so some data points are not visible because they overlap) and a **(e)** prior overweighting agent ($\omega_{1}$ = 1.12; an equivalent $\omega_{1}$ above 1 as the prior underweighting agent’s $\omega_{1}$ is below 1). The model predicts an interaction between bead-ratio condition and the final estimate difference for the prior underweighting and the prior overweighting agents. Methods are consistent with those described in Fig 2b. **(b, d, f)** Simulations, including all three bead ratio conditions (51:49, 60:40, 90:10), of the magnitude of updates in logit space as a function of the prior with respect to the color of the current evidence for a **(b)** prior underweighting agent, **(d)** an unbiased Bayesian observer and a **(f)** prior overweighting agent. Consistent the simulations throughout the manuscript, most agents are simulated with $\omega_{2(51:49)}$ = 1.0, $\omega_{2(60:40)}$ = 0.51, $\omega_{2(90:10)}$ = 0.50, with two exceptions. For the unbiased Bayesian observer **(d)**, we also illustrate the impact of variation in $\boldsymbol{\omega}_{\boldsymbol{2}}$**.** For the 90:10 bead ratio condition, we show the magnitude of the updates in logit space with an increased or decreased $\omega_{2(90:10)}$ ($\omega_{2(90:10)}$ = 0.4 or 0.6) relative to the primary simulations. These simulations are illustrated as transparent blue data in **(d)** with relatively thicker or thinner fit lines, compared to the primary simulation, for higher or lower $\omega_{2(90:10)}$ respectively. The purpose is to show that $\omega_{2}$ simply scales the magnitude of belief updates and that changes in $\omega_{2}$ alone are not sufficient to produce a recency bias or prior-dependent updating. Note that there are fewer data points in the more extreme bins for the base-rate neglecting agent because base-rate neglect mathematically induces an upper-boundary on certainty. The model predicts main effects of logit-prior and bead-ratio condition, but no interaction for the base-rate neglecting and base-rate overweighting agent.
