# Supplementary material for: The effects of base rate neglect on sequential belief updating and real-world beliefs
Source: PLoS Comput Biol. 2022 Dec 22;18(12):e1010796. doi: 10.1371/journal.pcbi.1010796 (PMC9831339; doi:10.1371/journal.pcbi.1010796)
Supplement: S3 Fig — (DOCX) [file pcbi.1010796.s034.docx]

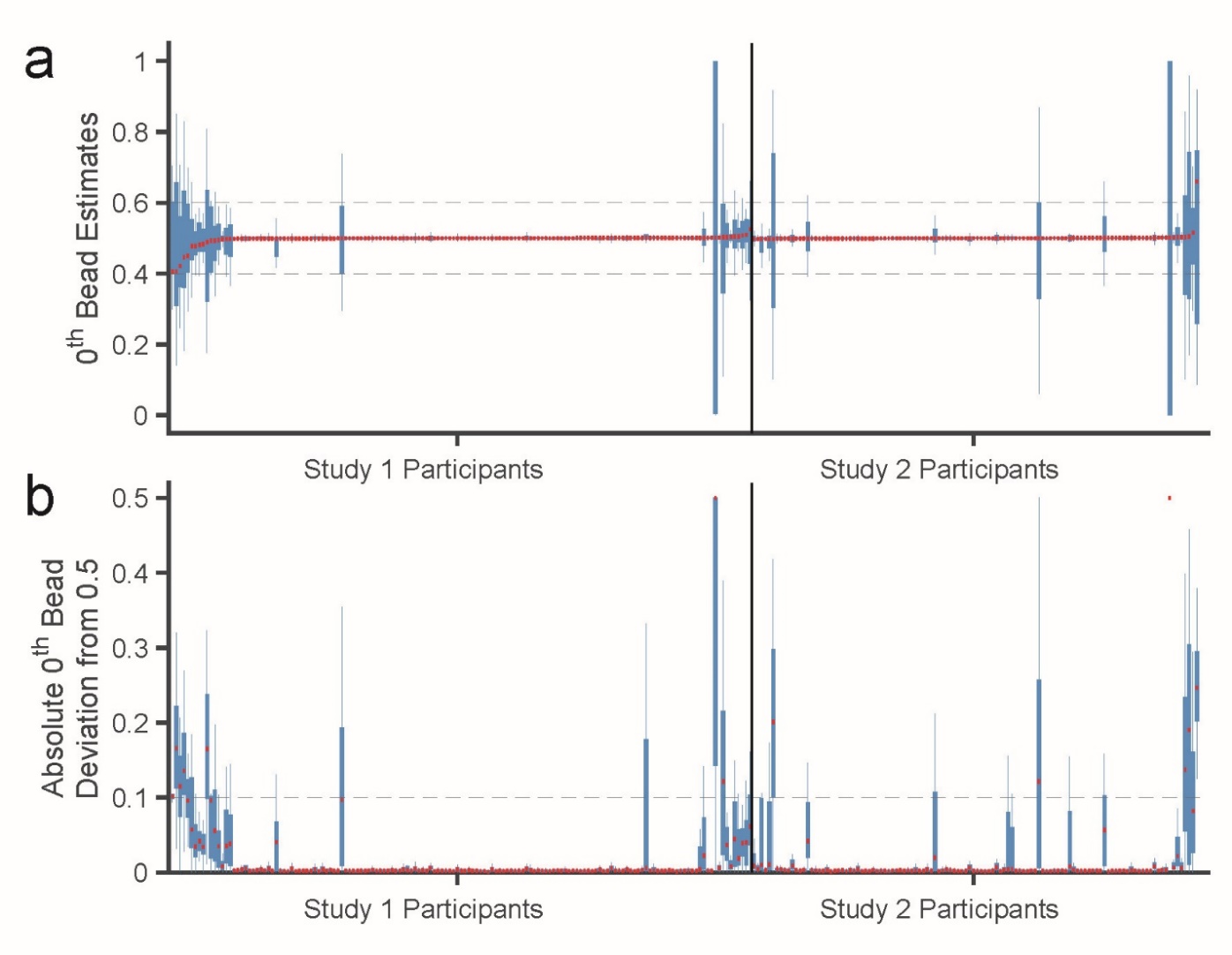


**S3 Fig. Probability estimates before presentation of the first bead (i.e., at the 0^th^ bead).** **(a)** Boxplots for all 0^th^ bead estimates (i.e., the estimate before presentation of the first bead on a trial) for each participant (55 per participant; one for each sequence), ordered from lowest to highest median. 0^th^ bead estimates were generally unbiased with nearly all participants reporting median 0^th^ bead estimates (red lines) around .5 and very few participants showed median 0^th^ bead estimates that appear to deviate substantially from 0.5. Furthermore, the majority of 0^th^ bead estimates were between 0.4 and 0.6 (dashed lines). **(b)** Boxplots for the absolute deviation of 0^th^ bead estimates from 0.5. Data ordering is consistent with (a). This was done to visualize if any participants were systematically deviating from a 0^th^ bead estimate of 0.5 regardless of the direction of that deviation. Most participants reported median 0^th^ bead estimates (red lines) that minimally deviated from 0.5. 254 of 267 participants’ average 0^th^ bead estimates deviated from 0.5 by less than 0.10 (~95% of participants; dashed line). The two most extreme outliers passed all of the exclusion criteria set forth in the methods, thus we did not exclude them from our analyses. *However, removing them from the analyses does not change any of the results in the study.* **(a, b)** The boxplots reflect the median (red lines), 25% percentile (lower boundary of box), 75^th^ percentile (upper boundary of box), 1.5 times the interquartile range above the median (upper whisker), and 1.5 times the interquartile range below the median (lower whisker) of the 0^th^ bead estimates for each participant. Taken together, these data suggest participants’ 0^th^ bead estimates were generally unbiased.
