# Supplementary material for: The effects of base rate neglect on sequential belief updating and real-world beliefs
Source: PLoS Comput Biol. 2022 Dec 22;18(12):e1010796. doi: 10.1371/journal.pcbi.1010796 (PMC9831339; doi:10.1371/journal.pcbi.1010796)
Supplement: S4 Fig — (DOCX) [file pcbi.1010796.s035.docx]

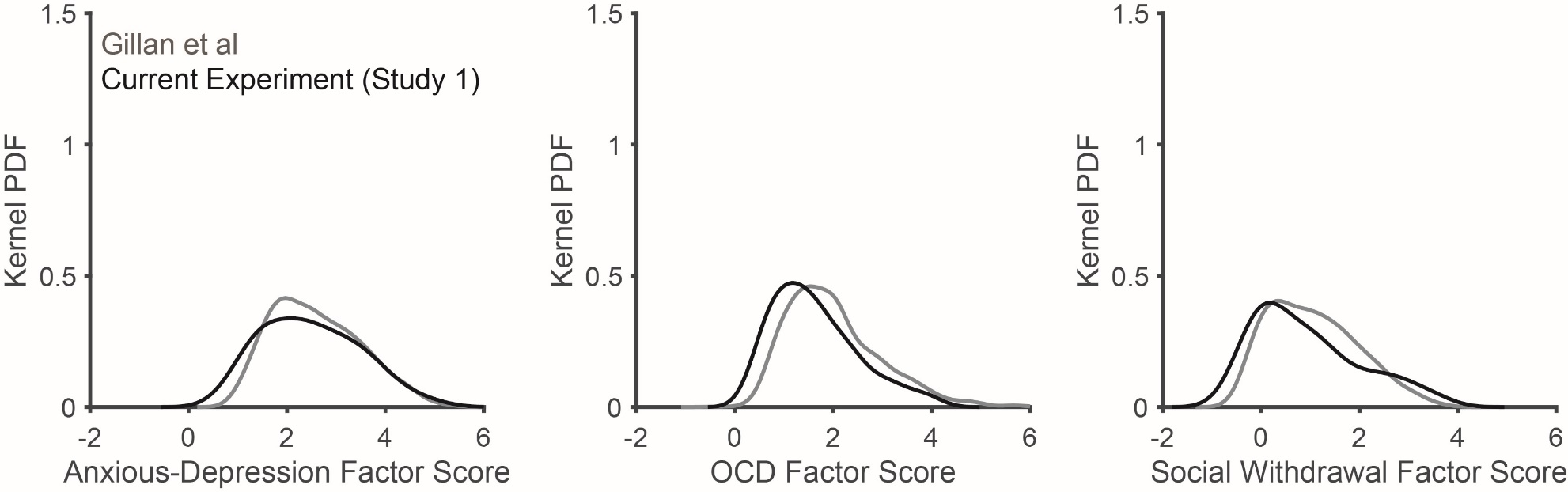


**S4 Fig. Comparison of sample’s general psychopathology factor scores to those in Gillan et al. (2016).**  Kernel-fit probability density functions for three broad dimensions of psychopathology for data from Gillan et al.[1] (Exp. 2, N = 1413; Grey line) and Study 1 (N = 143; black line). Gillan et al. conducted a factor analysis on an online sample of participant responses to a battery of 9 psychiatric questionnaires and identified a three-factor structure that explained the variance in responses: an anxious-depression factor, an OCD factor, and a social withdrawal factor. The 9 questionnaires included in the factor analysis were: the Obsessive Compulsive Inventory-Revised (OCI-R)[2], the Self-Rating Depression Scale (SDS)[3], the trait portion of the State-Trait Anxiety Inventory (STAI)[4], the Alcohol Use Disorder Identification Test (AUDIT)[5], the Barratt Impulsivity Scale (BIS-11)[6], the Apathy Evaluation Scale (AES)[7], the Eating Attitudes Test (EAT-26)[8], the Short Scales for Measuring Schizotypy (SSMS)[9], and the Liebowitz Social Anxiety Scale (LSAS)[10]. We obtained the participant data and item-wise factor weights through correspondence with the authors and calculated the factor scores for each participant in their sample. In the Study 1 sample, after the probability estimates task, 143 of the 151 participants also completed the same 9 questionnaires as in Gillan et al. (some participants dropped out after completing the task, but before finishing the questionnaires). Participants also completed a 9-item Raven’s Matrix[11] but this was not part of the factor analysis. We used the item weights from Gillan et al. to calculate factor scores for the study 1 sample. We used the factor scores and the Raven’s Matrix to assess if general psychopathology and/or general cognition may be driving the critical model-based effects identified in our study (S10-S13 Table). Here, we show that we sampled from participants with a highly similar range of factor scores as Gillan et al., generally supporting the validity of these analyses (i.e., using their factor weights on our data) and the notion that our data had sufficient range in these scores to detect relationships with task and model variables.

References

1. Gillan CM, Kosinski M, Whelan R, Phelps EA, Daw ND. Characterizing a psychiatric symptom dimension related to deficits in goal-directed control. Elife. 2016;5: e11305. doi:10.7554/eLife.11305

2. Foa EB, Huppert JD, Leiberg S, Langner R, Kichic R, Hajcak G, et al. The Obsessive-Compulsive Inventory: development and validation of a short version. Psychol Assess. 2002;14: 485–496.

3. ZUNG WWK. A Self-Rating Depression Scale. Archives of General Psychiatry. 1965;12: 63–70. doi:10.1001/archpsyc.1965.01720310065008

4. Spielberger CD, Gorsuch R, Lushene R, Vagg P, Jacobs G. State-trait anxiety inventory. Palo Alto. CA: Mind Garden. 1983.

5. Saunders JB, Aasland OG, Babor TF, De La Fuente JR, Grant M. Development of the Alcohol Use Disorders Identification Test (AUDIT): WHO Collaborative Project on Early Detection of Persons with Harmful Alcohol Consumption-II. Addiction. 1993;88: 791–804. doi:10.1111/j.1360-0443.1993.tb02093.x

6. Patton JH, Stanford MS, Barratt ES. Factor structure of the barratt impulsiveness scale. Journal of Clinical Psychology. 1995;51: 768–774. doi:10.1002/1097-4679(199511)51:6<768::AID-JCLP2270510607>3.0.CO;2-1

7. Marin RS, Biedrzycki RC, Firinciogullari S. Reliability and validity of the apathy evaluation scale. Psychiatry Research. 1991;38: 143–162. doi:10.1016/0165-1781(91)90040-V

8. Garner DM, Olmsted MP, Bohr Y, Garfinkel PE. The Eating Attitudes Test: psychometric features and clinical correlates. Psychological Medicine. 1982;12: 871–878. doi:10.1017/S0033291700049163

9. Mason O, Linney Y, Claridge G. Short scales for measuring schizotypy. Schizophrenia Research. 2005;78: 293–296. doi:10.1016/j.schres.2005.06.020

10. Liebowitz MR. Social Phobia. Anxiety. 1987;22: 141–173. doi:10.1159/000414022

11. Bilker WB, Hansen JA, Brensinger CM, Richard J, Gur RE, Gur RC. Development of Abbreviated Nine-item Forms of the Raven’s Standard Progressive Matrices Test. Assessment. 2012;19: 354–369. doi:10.1177/1073191112446655
