# Supplementary material for: The effects of base rate neglect on sequential belief updating and real-world beliefs
Source: PLoS Comput Biol. 2022 Dec 22;18(12):e1010796. doi: 10.1371/journal.pcbi.1010796 (PMC9831339; doi:10.1371/journal.pcbi.1010796)
Supplement: S5 Fig — (DOCX) [file pcbi.1010796.s036.docx]

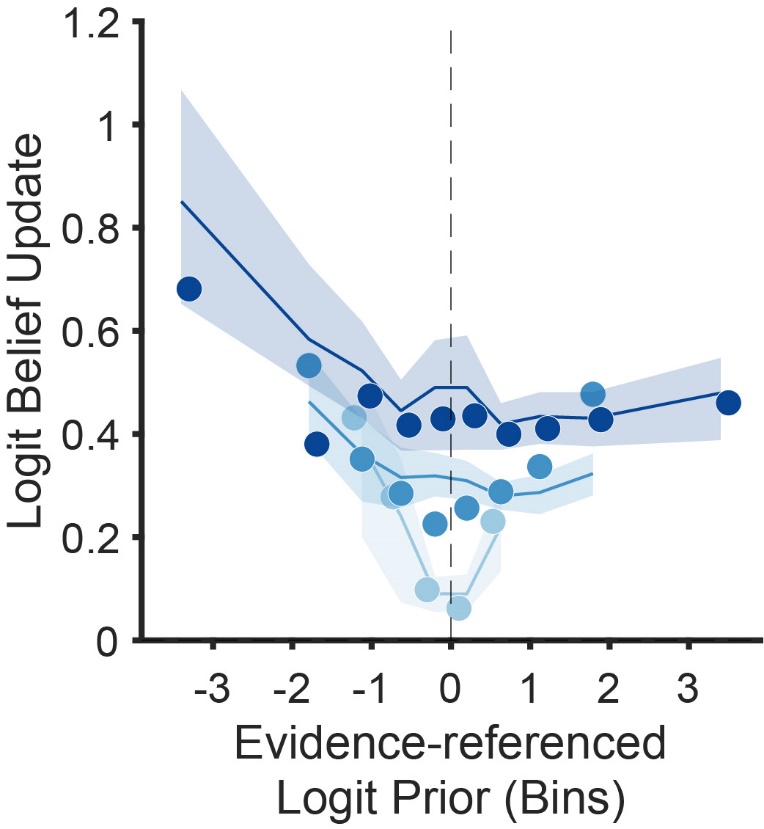


**S5 Fig.** **Logit-belief updates as a function of logit prior by bead ratio for the main sample in study 2 (N = 91).** This is comparable to the analysis illustrated in Figure 3c, but for the main sample in study 2. Group medians of individual medians for logit-belief updates are shown and other conventions follow Fig 3c. Solid lines and shaded regions reflect medians and 95% bootstrapped confidence intervals of the weighted Bayesian model fits. These data are consistent with the base-rate neglect induced prior dependent updating slope predicted by the weighted Bayesian model in Fig 2c.
