# Supplementary material for: The effects of base rate neglect on sequential belief updating and real-world beliefs
Source: PLoS Comput Biol. 2022 Dec 22;18(12):e1010796. doi: 10.1371/journal.pcbi.1010796 (PMC9831339; doi:10.1371/journal.pcbi.1010796)
Supplement: S6 Fig — Formal model comparison for data from (a) study 1 and (b) study 2. (DOCX) [file pcbi.1010796.s037.docx]

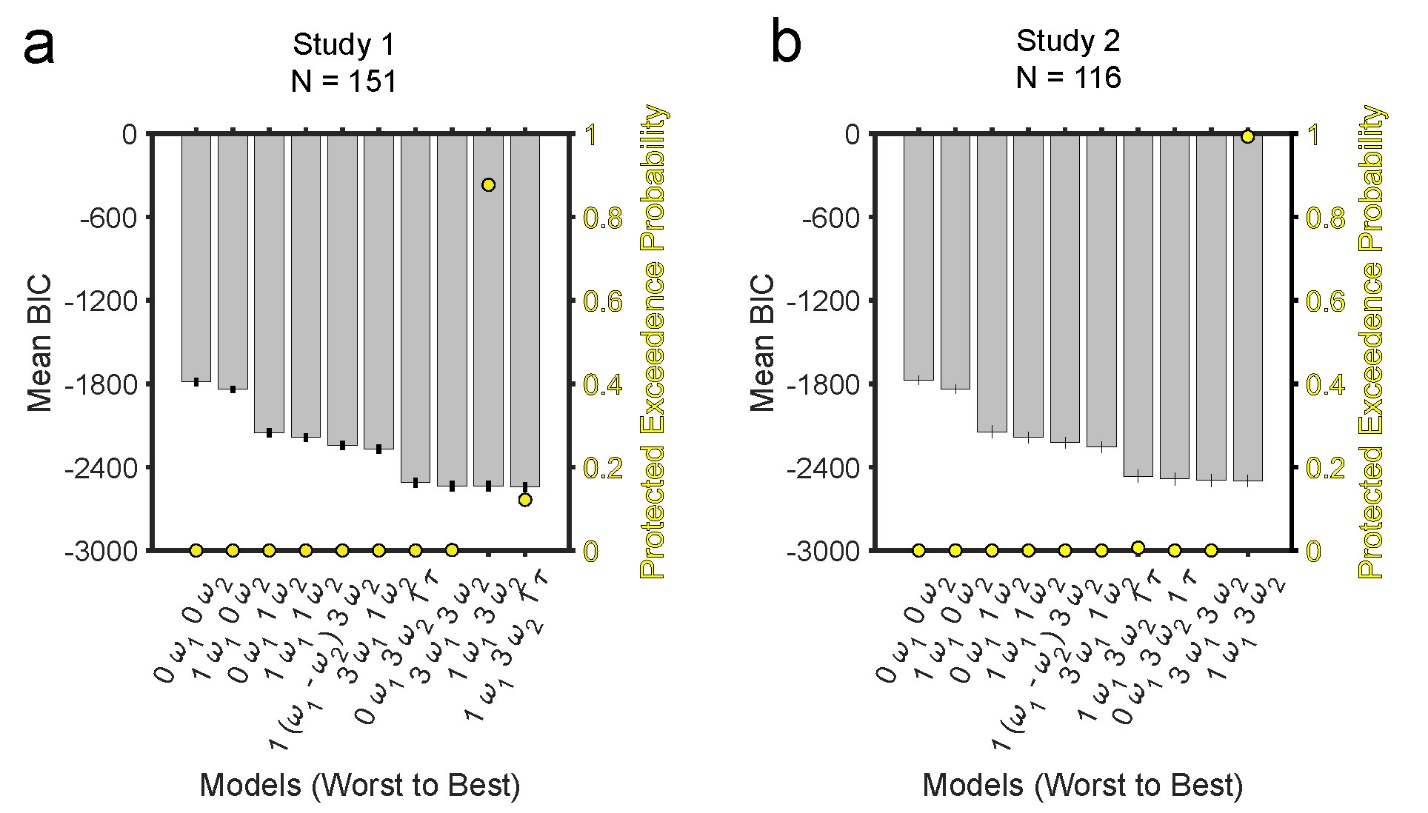


**S6 Figure. Formal model comparison for data from (a) study 1 and (b) study 2.** This Figure illustrates mean BIC across participants (from worst to best based on BIC) and the protected exceedance probability. The winning model was defined as the model with the highest protected exceedance probability. The study 1 data has been reproduced from the main text to make it easier to directly compare model fits. We compared 10 different models (S27 Table), consistent with Baker et al.[1]. In both studies, the winning model was the same as the winning model in Baker et al [1]; it included a single prior weight parameter across all conditions, and three likelihood weight parameters (one per bead-ratio condition).

References

1. Baker SC, Konova AB, Daw ND, Horga G. A distinct inferential mechanism for delusions in schizophrenia. Brain. 2019;142: 1797–1812. doi:10.1093/brain/awz051
