# Supplementary material for: The effects of base rate neglect on sequential belief updating and real-world beliefs
Source: PLoS Comput Biol. 2022 Dec 22;18(12):e1010796. doi: 10.1371/journal.pcbi.1010796 (PMC9831339; doi:10.1371/journal.pcbi.1010796)
Supplement: S7 Fig — (DOCX) [file pcbi.1010796.s038.docx]

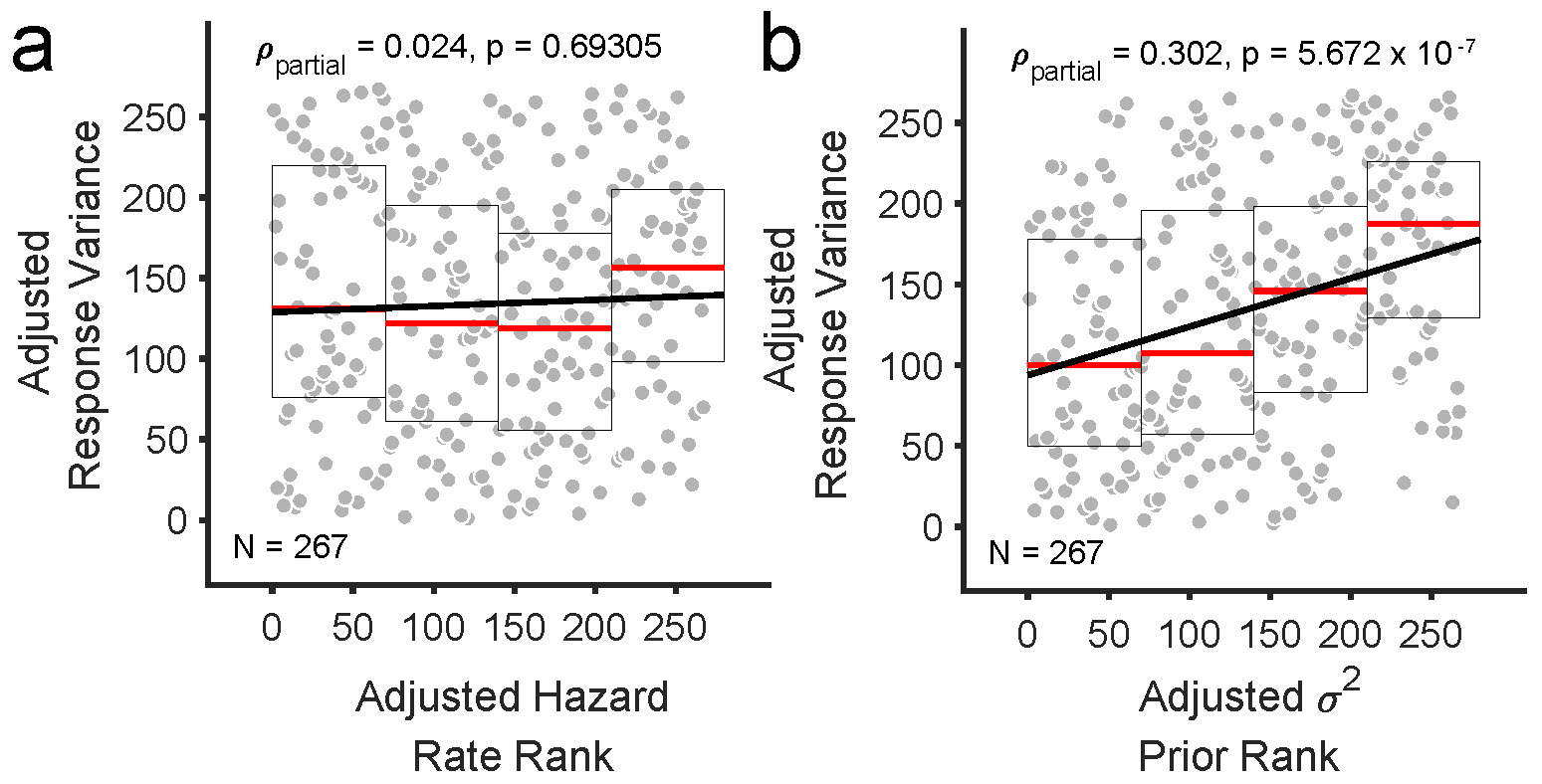


**S7 Fig. Predicted relationships between parameters governing prior integration and response variability in (a) a volatility model and (b) the noisy sampling model.** Observers with model parameters in the range of the real data were simulated on the beads task. Simulated observers started each trial at uncertainty with regard to the correct box and the posterior estimate after each draw served as the prior for the subsequent draw. Response variance computed from simulated task responses was compared to the concomitant prior weight parameter: Hazard rate for the volatility model and $\sigma_{prior}^{2}$ for the noisy sampling model. The volatility model does not predict a systematic relationship between its prior weight parameter and unstructured behavioral noise (response variance), but the noisy sampling model does. The data points were adjusted controlling for **(a)** all three $\omega_{2_{(Likelihood)}}$ or **(b)** all three $\sigma_{Likelihood}^{2}$ parameters consistent with reported partial correlation values. These simulations were designed for comparison to Figure 6d, which reflects the real data.
