# Supplementary material for: The effects of base rate neglect on sequential belief updating and real-world beliefs
Source: PLoS Comput Biol. 2022 Dec 22;18(12):e1010796. doi: 10.1371/journal.pcbi.1010796 (PMC9831339; doi:10.1371/journal.pcbi.1010796)
Supplement: S8 Fig — (DOCX) [file pcbi.1010796.s039.docx]

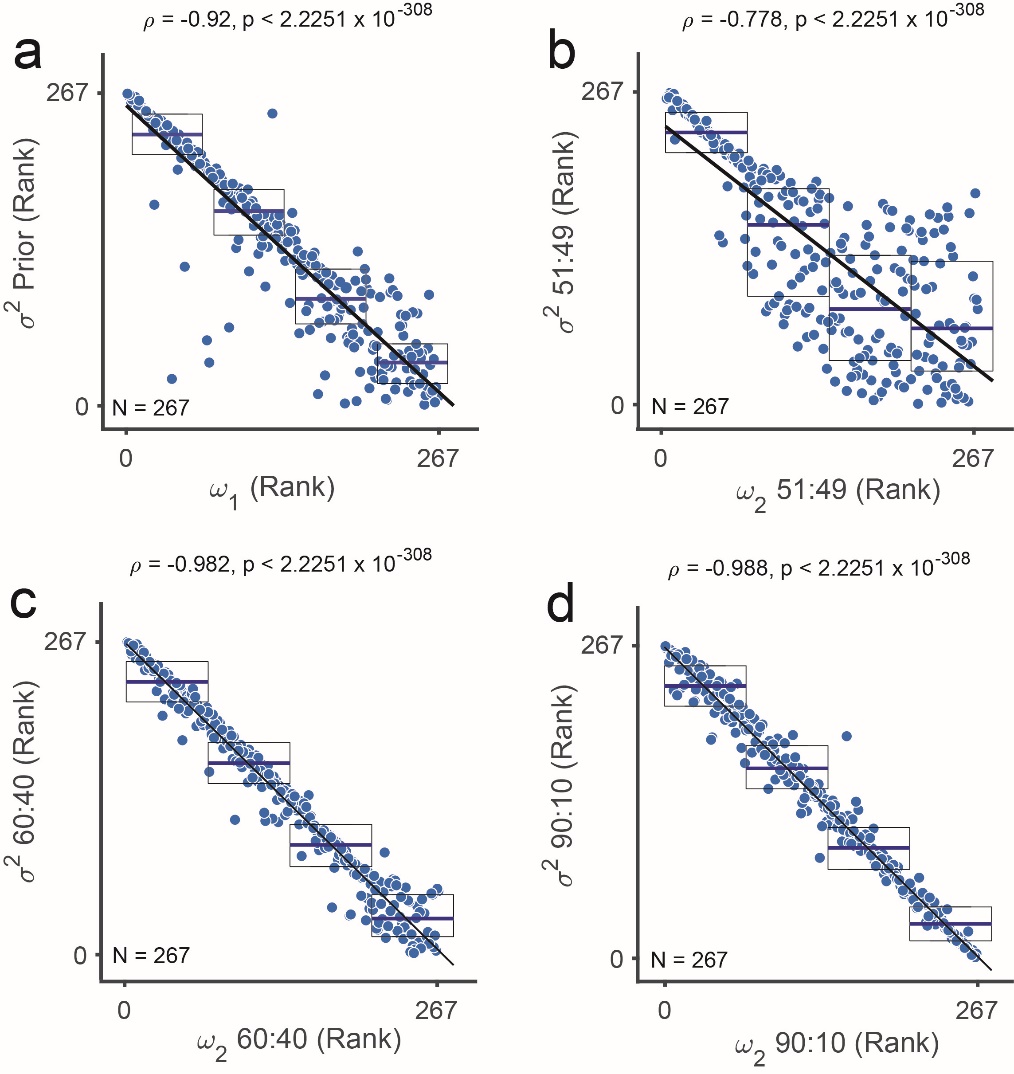


**S8 Fig. Scatterplots and Spearman correlations of corresponding prior and likelihood parameters from the weighted Bayesian model (**$\boldsymbol{\omega}_{\boldsymbol{(Likelihood)}}^{\boldsymbol{2}}$**) and noisy sampling model (**$\boldsymbol{\sigma}_{\boldsymbol{(Likelihood)}}^{\boldsymbol{2}}$**).** These figures reflect the **(a)** prior parameters and the likelihood parameters for the **(b)** 51:49, **(c)** 60:40, and **(d)** 90:10 bead ratio conditions
