# Supplementary material for: The effects of base rate neglect on sequential belief updating and real-world beliefs
Source: PLoS Comput Biol. 2022 Dec 22;18(12):e1010796. doi: 10.1371/journal.pcbi.1010796 (PMC9831339; doi:10.1371/journal.pcbi.1010796)
Supplement: S9 Fig — (DOCX) [file pcbi.1010796.s040.docx]

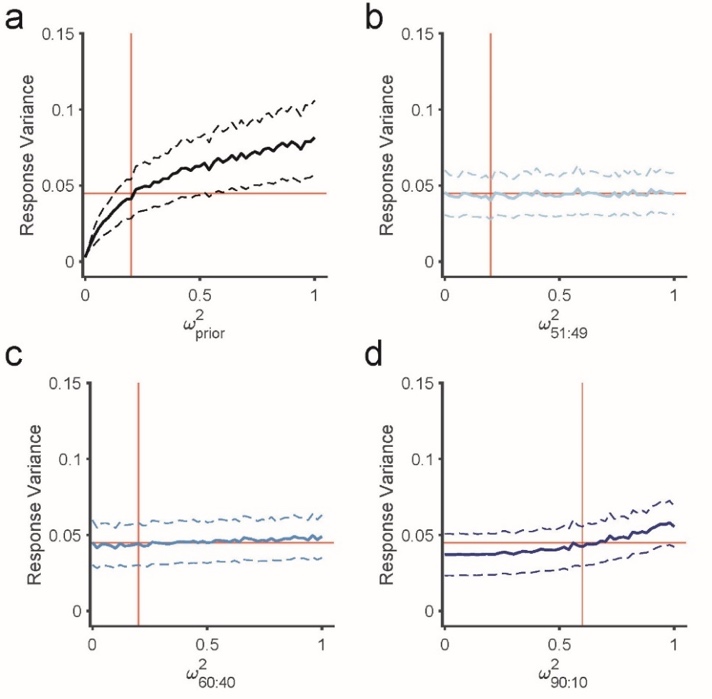


**S9 Fig. Simulations varying individual** $\boldsymbol{\omega}^{\boldsymbol{2}}$ **parameters in the noisy sampling model, while holding all other parameters constant.** We simulated data based on each participant’s best fitting parameter values (both $\sigma^{2}$’s and group-level $\omega^{2}$ values) and using their 0^th^ bead estimates as starting points for the simulations, except for the $\omega^{2}$ parameter of interest which was parametrically iterated from 0 – 1 in steps of .02 (51 iterations). For each Figure, this resulted in 267 simulations per $\omega^{2}$ parameter iteration. For each simulated participant we calculated the response variance, and obtained the mean (solid line) and standard error of the mean (dashed lines) of the response variance across participant simulations for each parameter iteration. **(a)** shows the effects of varying $\omega_{prior}^{2}$, **(b)** shows the effects of varying $\omega_{51:49}^{2}$, **(c)** shows the effects of varying $\omega_{60:40}^{2}$, and **(d)** shows the effects of varying $\omega_{90:10}^{2}$. The vertical red line indicates the $\omega^{2}$ value we used in our model fitting procedure in the main analysis. The horizontal red line indicates the median response variance from the raw data. Here we show that $\omega_{prior}^{2}$ appears to impact response variance in a manner similar to $\sigma_{prior}^{2}$; greater variance in the underlying logit prior distribution is associated with higher response variance. Since participants are explicitly informed of the variance in the underlying logit prior and likelihood distributions and since these distributions are held constant over the course of the study, to avoid tradeoffs between the $\omega_{prior}^{2}$ and the $\sigma_{prior}^{2}$ we concluded it was reasonable to hold $\omega_{prior}^{2}$ constant across participants during our model fitting procedures (see Methods). The $\omega_{likelihood}^{2}$ parameters do not appear to substantially impact response variance, and thus we could also hold these constant as well. In short, our results are not likely to be driven by our decision to hold the $\omega_{prior}^{2}$ and the $\omega_{likelihood}^{2}$ parameters constant across participants.
