# Supplementary material for: The effects of base rate neglect on sequential belief updating and real-world beliefs
Source: PLoS Comput Biol. 2022 Dec 22;18(12):e1010796. doi: 10.1371/journal.pcbi.1010796 (PMC9831339; doi:10.1371/journal.pcbi.1010796)
Supplement: S10 Fig — (DOCX) [file pcbi.1010796.s041.docx]

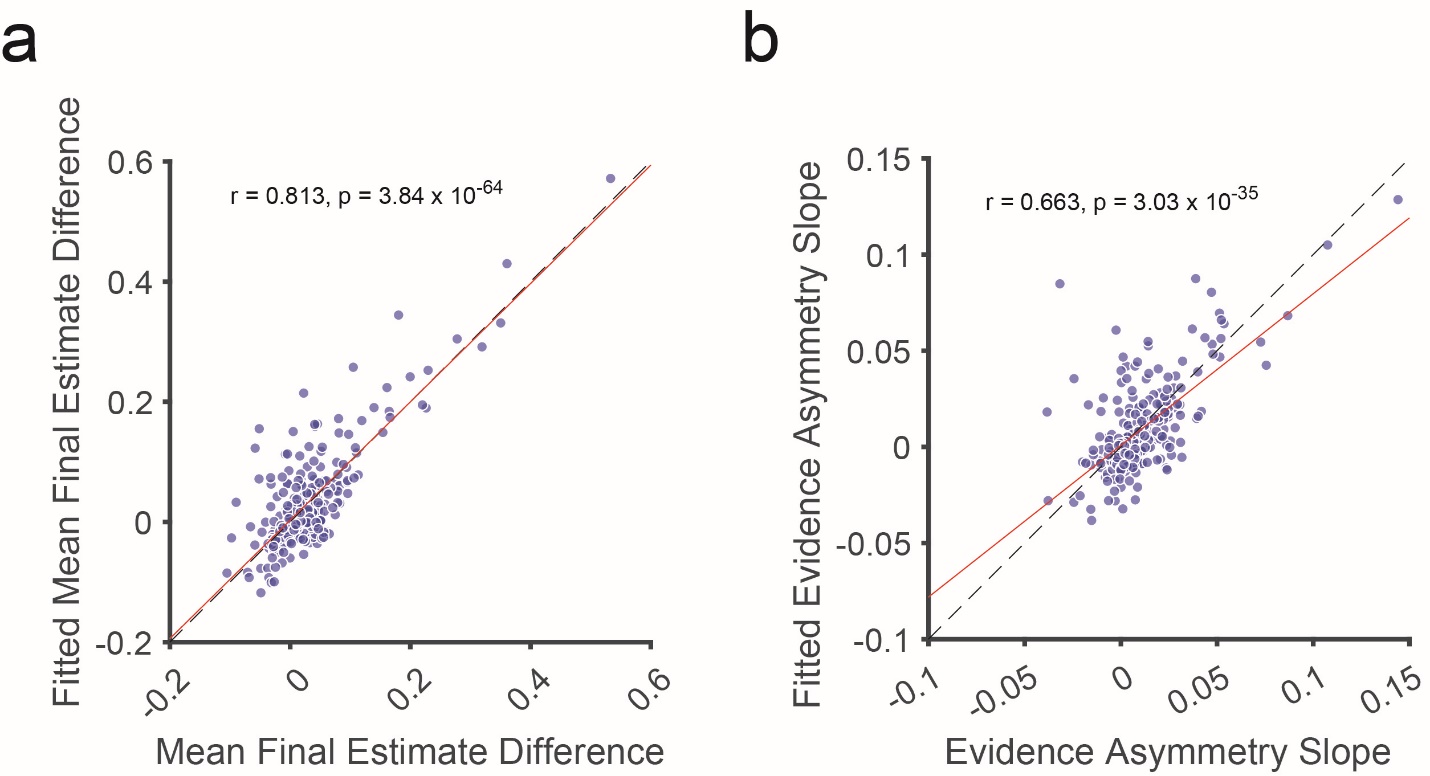


**S10 Fig. Posterior predictive checks for mean final estimate difference and evidence asymmetry slope.** We simulated data based on the best-fitting parameters for each of the 267 participants and calculated the model-agnostic measures of mean final estimate difference and evidence asymmetry based on these simulated data. **(a)** Correlation between the fitted and real mean final estimate difference. **(b)** Correlation between the fitted and real evidence asymmetry slope. **(a, b)** The simulated and real data correlated highly for both measures (unity line reflected by dashed black line, least squares fit line reflected by solid red line). Overall, these checks suggest that our data (including the range of observed values) are well captured by the model.
