# Supplementary material for: The effects of base rate neglect on sequential belief updating and real-world beliefs
Source: PLoS Comput Biol. 2022 Dec 22;18(12):e1010796. doi: 10.1371/journal.pcbi.1010796 (PMC9831339; doi:10.1371/journal.pcbi.1010796)
Supplement: S11 Fig — (DOCX) [file pcbi.1010796.s042.docx]

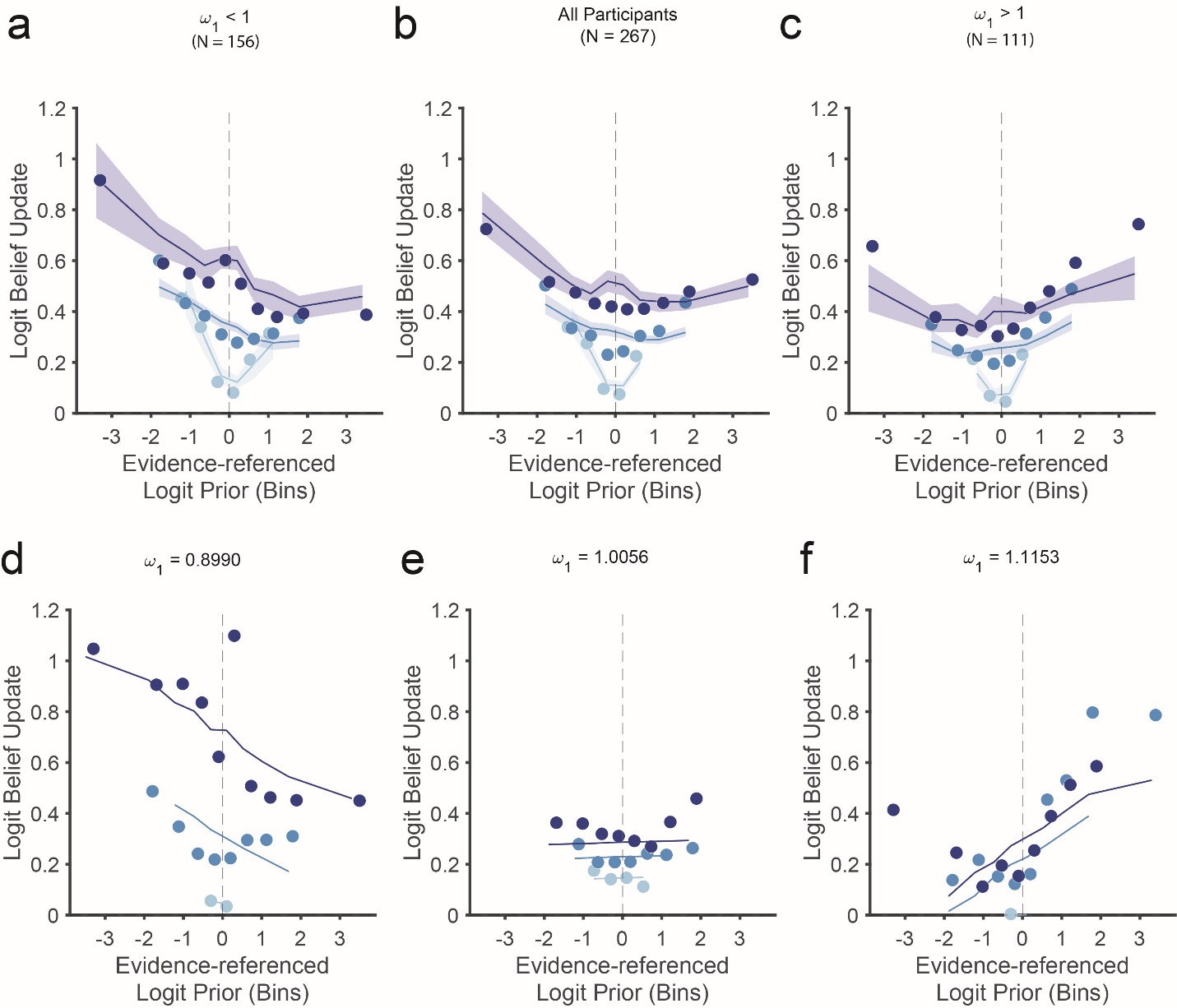


**S11 Fig: Posterior predictive checks for logit belief updates.** Logit belief update across (a) participants with $\omega_{1}<1$, (b) all 267 participants, and (c) participants with $\omega_{1}>1$. (**a, b, and c)**. Data reflects the group median of the individual medians of the magnitude of logit-belief updates as a function of the logit prior with respect to the color of the current evidence by bead-ratio condition. The x-axis is discretized into bins equivalent to 0.1 increments of the prior belief in probability space (with a lower limit of 0.01 and an upper limit of 0.99). Data are only binned for visualization. The y-axis represents the magnitude of the logit-belief updates (the difference in the log-odds of the prior and posterior beliefs). Solid lines and shaded regions reflect medians and 95% bootstrapped confidence intervals of the weighted Bayesian model fits. The model fit and the data track closely with one another in a, b, and c. Although not displayed for visual clarity, the confidence intervals for the raw data overlap substantially with the model fits. **(d, e, f)** We also present the logit belief update Figure for three representative participants with a (d) low $\omega_{1}$, (e) middle-range $\omega_{1}$, (f) and high $\omega_{1}$ in our sample. The dots reflect raw data and the lines reflect model fits. The relationship between logit-prior and logit belief updates is consistent with our predictions at the level of individuals, not just group averages.
