# Supplementary material for: The effects of base rate neglect on sequential belief updating and real-world beliefs
Source: PLoS Comput Biol. 2022 Dec 22;18(12):e1010796. doi: 10.1371/journal.pcbi.1010796 (PMC9831339; doi:10.1371/journal.pcbi.1010796)
Supplement: S12 Fig — (DOCX) [file pcbi.1010796.s043.docx]

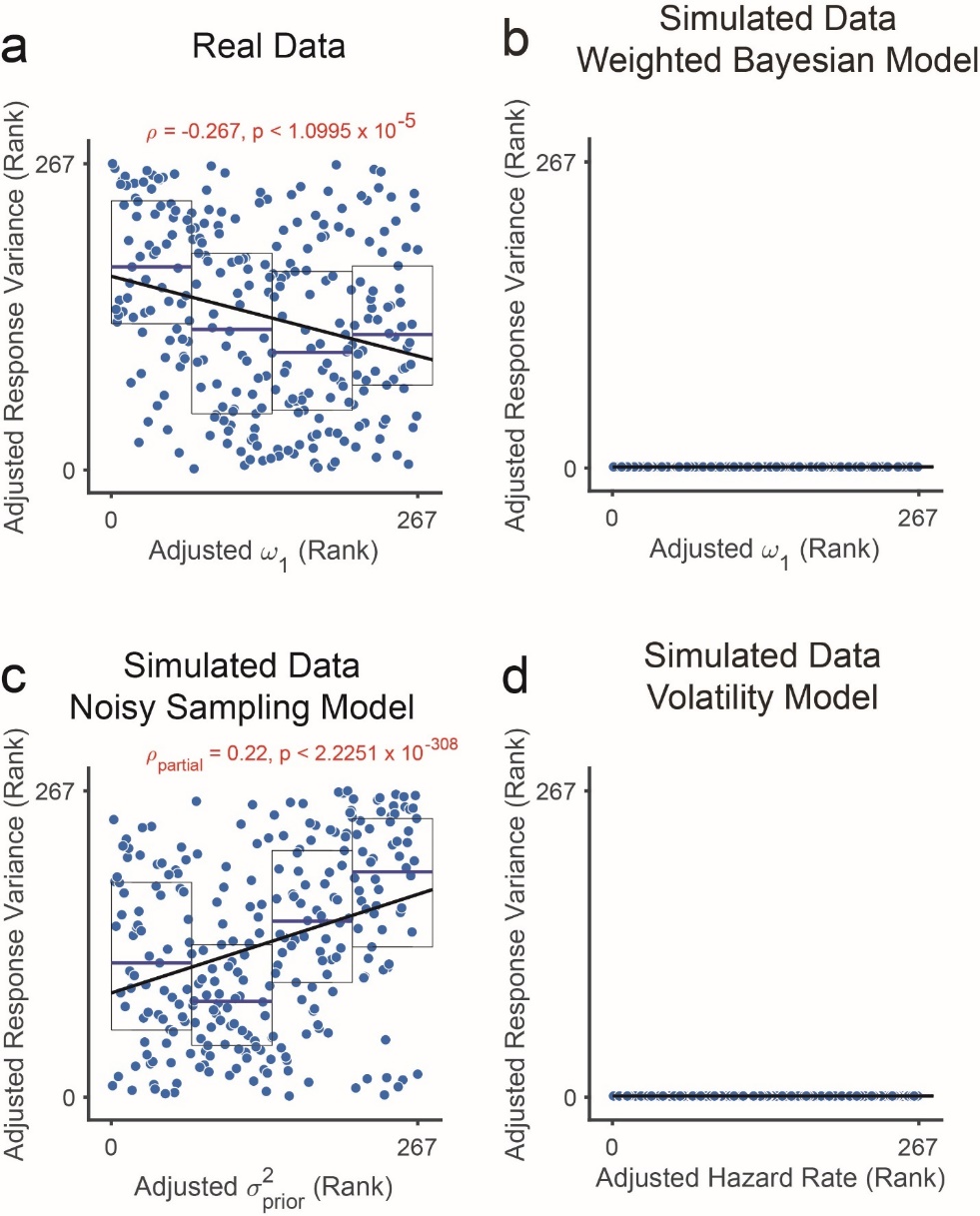


**S12 Fig. Posterior predictive checks for response variance.** This Figure shows the relationship between $\omega_{1}$ and the response variance based on **(a)** the real data, **(b)** data simulated with the weighted Bayesian model, **(c)** data simulated with the noisy sampling model, and **(d)** data simulated with the volatility model. The noisy sampling model is the only one of the three models that is able to reproduce the relationship between response variance and prior weight. For comparison, we show that the relationship found in the real data matches that predicted by the model. Note that the relationship is inverted because low $\omega_{1}$ is equivalent to high $\sigma_{prior}^{2}.$ **(b, c, d)** Data was simulated using the best-fitting parameters for each of the models. **(a – d)** Partial correlations control for the appropriate model parameters ($\omega_{2 (likelihood)}$ for the real data and $\sigma_{likelihood}^{2}$ for the noisy sampling model).
