# Supplementary material for: The effects of base rate neglect on sequential belief updating and real-world beliefs
Source: PLoS Comput Biol. 2022 Dec 22;18(12):e1010796. doi: 10.1371/journal.pcbi.1010796 (PMC9831339; doi:10.1371/journal.pcbi.1010796)
Supplement: S13 Fig — (DOCX) [file pcbi.1010796.s044.docx]

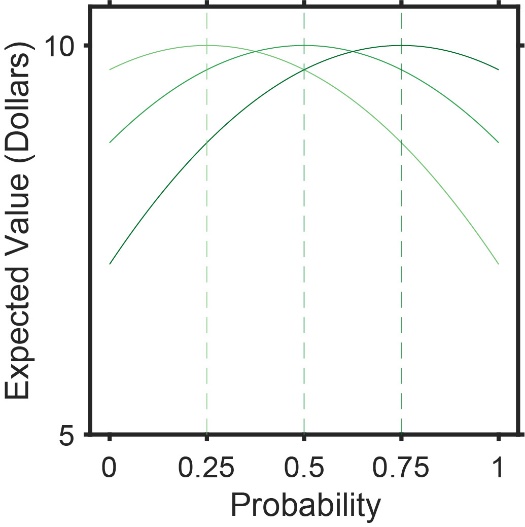


**S13 Fig. The binarized scoring rule maximizes expected value for accurate probability estimates.** We implemented a binarized scoring rule to isolate rewards to incentivize estimation accuracy while accounting for risk attitudes[1]. On the x-axis are simulated probability estimates, $\pi\left( Q | x \right)$, and on the y-axis is the expected value of an estimate assuming, as in the task, an endowment, N, of $10 and a potential penalty, W, of $5. Expected value is calculated for three situations, in which the objective posterior probability, $P \left( Q | x \right)$, is either 0.25, 0.50, or 0.75 (lighter to darker green, respectively), reflected by the dashed vertical lines. It is clear that the highest expected value is obtained in each condition when $P \left( Q | x \right)=\pi\left( Q | x \right)$, regardless of the magnitude of $P \left( Q | x \right)$. By applying a quadratic loss function to the probability of obtaining a big or small reward, the binarized scoring rule establishes a U-shaped rather than linear relationship between expected value, $EV,$ and the posterior probability, $\pi\left( Q | x \right)$. In other words, participants get more money for reporting accurate estimates (i.e., estimates closer to the true Bayesian estimate), not for reporting higher (or lower) probabilities.

The loss function for the binarized scoring rule takes this form: $p_{loss}=\left( P \left( Q | x \right)-\pi\left( Q | x \right) \right)^{2}$, where $p_{loss}$ represents the probability the participant will lose money from their endowment, $\pi(Q|x)$ is the participant’s subjective probability estimate on a trial, and $P \left( Q | x \right)$ is the objective posterior probability given the ideal Bayesian observer. Therefore, $p_{loss}$ is effectively a measure of the participant’s error and is minimized when $P \left( Q | x \right)=\pi(Q|x)$. Manipulating the *probability* of losing a pre-set and consistent reward, instead the amount of reward lost, makes the scoring rule insensitive to differing risk preferences among participants [1]. Using this loss function, we calculated the expected value ($EV$) of a trial, which is the sum of two products: (1) the endowment $N$ and the probability of keeping the endowment and (2) the endowment minus the penalty $W$ and the probability of losing the penalty from the endowment: $EV=N\left( 1-p_{loss} \right)+\left( N-W \right)p_{loss}$. Filling in the equation for $p_{loss}$ and then simplifying gives: $EV=N-\left( W\cdot{P \left( Q | x \right)}^{2} \right)+ \left( 2\cdot W\cdot P \left( Q | x \right)\cdot\pi\left( Q | x \right) \right)-\left( W\cdot{\pi\left( Q | x \right)}^{2} \right)$.

References

1. Hossain T, Okui R. The binarized scoring rule. Review of Economic Studies. 2013;80: 984–1001. doi:10.1093/restud/rdt006
