# Supplementary material for: The effects of base rate neglect on sequential belief updating and real-world beliefs
Source: PLoS Comput Biol. 2022 Dec 22;18(12):e1010796. doi: 10.1371/journal.pcbi.1010796 (PMC9831339; doi:10.1371/journal.pcbi.1010796)
Supplement: S14 Fig — (DOCX) [file pcbi.1010796.s045.docx]

**
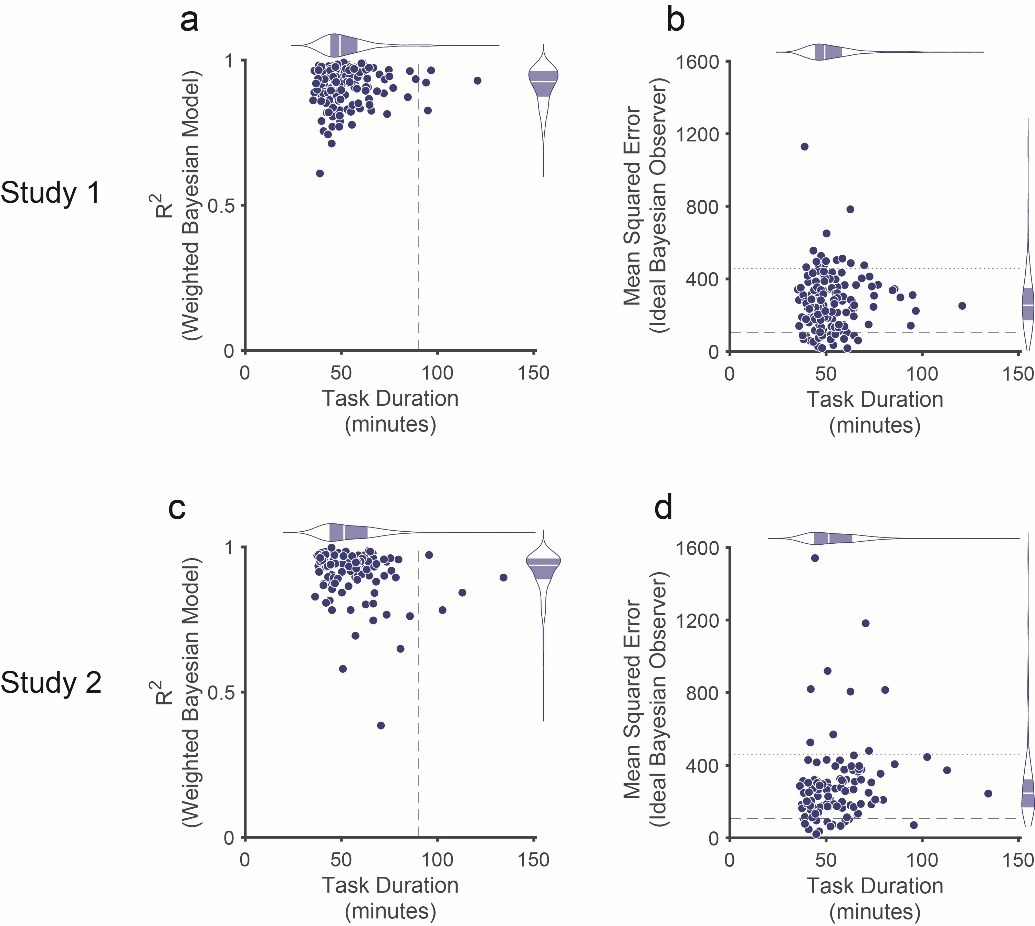
**

**S14 Fig. Data Quality Checks 1: Weighted Bayesian model fit, mean squared error, task duration.** Given the online data collection, there may be some concerns about data quality. As described in the methods, we applied several stringent exclusion criteria to ensure high quality data. Evidence supporting the effectiveness of our efforts are provided in **(a – d). (a)** and **(c)** show the distributions of, and relationship between, the R^2^ model fits to the winning weighted Bayesian model and the time participants took to complete the task for study 1 and 2, respectively. During in-person piloting, this task took approximately 45 minutes to complete half as many trials as in these studies. Consistent with this, and despite having a 4-hour time window, **(a)** and **(b)** show that 97% of participants in study 1 and study 2 finished the task within 90 minutes (dashed vertical line). Furthermore, the distribution of R^2^ shows that the winning model fit participants’ data well, with 98% of participants across study 1 and study 2 having an R^2^ > 0.70. Both of these results are very consistent with our in-person participants, and data quality expectations for study 1 given participants’ Mturk “masters” status[1]. Taken together, these data suggests that the online participants were not inattentive or multitasking. **(b)** and **(d)** show the distributions of, and relationship between, the mean squared error of subject estimates relative to the ideal Bayesian observer and the time participants took to complete the task for study 1 and 2, respectively. In both samples, the error was within the expected range. As a reference, we included the mean squared error (horizontal dashed line) plus 2 standard deviations (horizontal dotted line) from previous work that used the same incentive structure[2]. In general, the error in our data is consistent with this previous work which used the same incentive structure. This suggests that participants understood our incentive structure and that it was effective.

References

1. Peer E, Vosgerau J, Acquisti A. Reputation as a sufficient condition for data quality on Amazon Mechanical Turk. Behav Res. 2014;46: 1023–1031. doi:10.3758/s13428-013-0434-y

2. Hossain T, Okui R. The binarized scoring rule. Review of Economic Studies. 2013;80: 984–1001.
