# Supplementary material for: The effects of base rate neglect on sequential belief updating and real-world beliefs
Source: PLoS Comput Biol. 2022 Dec 22;18(12):e1010796. doi: 10.1371/journal.pcbi.1010796 (PMC9831339; doi:10.1371/journal.pcbi.1010796)
Supplement: S15 Fig — (DOCX) [file pcbi.1010796.s046.docx]

**
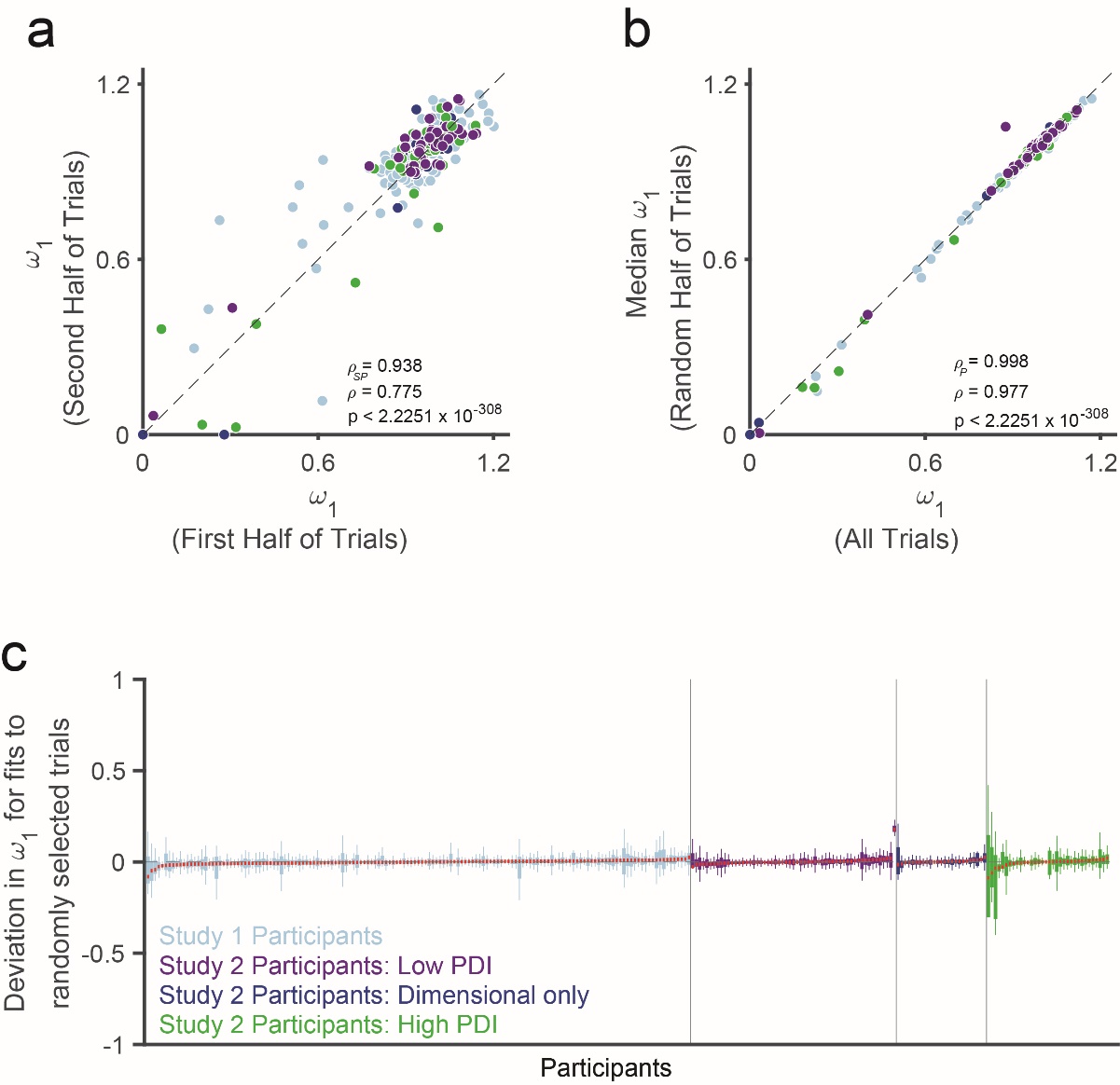
**

**S15 Fig. Data Quality Checks 2: Evidence for behavioral consistency across the probability estimates beads task.** One concern about online samples is inattention. Despite good model fits, one may be concerned that participants were only paying attention to a part of the task (i.e., only early on or only late in the task) and that period of time is driving the modelling results. **(a)** To address this concern, we fit the weighted Bayesian model (using the same procedure described in the methods) to the first half of trials and the second half of trials that each participant completed in order to calculate the split-half reliability of $\omega_{1}$. Note that the first and second half of trials included an equal number of 60:40 and 90:10 trials (11 each). As there were an odd number of 51:49 trials, we included 6 of these trials in first half and 5 in the second half. There was a significant Spearman correlation between $\omega_{1}$ for fits to the first and second half of the data, suggesting participants were responding consistently over the course of the experiment. Following the conventions set by prior work[1], we also report the split-half parallel reliability ($\rho_{SP}$) which indicates robust reliability. **(b)** As a further test, we conducted a bootstrap analysis where we fit the weighted Bayesian model to 100 random samples of half of the trials, with the caveat that the random sample had to include half of the 60:40 and 90:40 trials and half, rounded up because there are an odd number of these trials, of the 51:49 trials (6 trials). Here, we show the median $\omega_{1}$ per participant across the 100 bootstrapped samples. There was a significant Spearman correlation between $\omega_{1}$ for model fits to the entire data set and to the median of the bootstrapped samples. Consistent with (a), we found that a general parallel reliability ($\rho_{P}$) indicates robust reliability. **(c)** Finally, to illustrate that the findings from random sampling of the trials is not simply an artifact of averaging the $\omega_{1}$’s within participants, for each participant we calculated the differences between the $\omega_{1}$ from fitting all of the trials and for each of the $\omega_{1}$’s obtained from random sampling of the trials. This Figure shows boxplots of 100 deviations from the $\omega_{1}$’s from the main text for each participant. It is evident that most participants show limited variance and a median near 0. Although there is more variance for some of the high PDI participants, this is expected based on our models (high PDI = low $\omega_{1}$ = high $\sigma_{prior}^{2}$= high behavioral variance) and critically their medians tend to be around 0. The boxplots reflect the median (red lines), 25% percentile (lower boundary of box), 75^th^ percentile (upper boundary of box), 1.5 times the interquartile range above the median (upper whisker), and 1.5 times the interquartile range below the median (lower whisker). **(a, b, c)** Overall, these analyses provide substantial evidence of behavioral consistency within participants across the entire task, supports the notion that this is high quality data, and speaks against the notion that participants were inattentive during the task. Participant data is color coded. Light blue data points indicate participants from study 1, purple data indicates low PDI participants from study 2, green data indicates high PDI participants from study 2, and dark blue data indicates all remaining participants from study 2 that were only included in the exploratory dimensional analyses.

References

1. Cho E. Making Reliability Reliable: A Systematic Approach to Reliability Coefficients. Organizational Research Methods. 2016;19: 651–682. doi:10.1177/1094428116656239
