# Supplementary material for: The effects of base rate neglect on sequential belief updating and real-world beliefs
Source: PLoS Comput Biol. 2022 Dec 22;18(12):e1010796. doi: 10.1371/journal.pcbi.1010796 (PMC9831339; doi:10.1371/journal.pcbi.1010796)
Supplement: S16 Fig — (DOCX) [file pcbi.1010796.s047.docx]

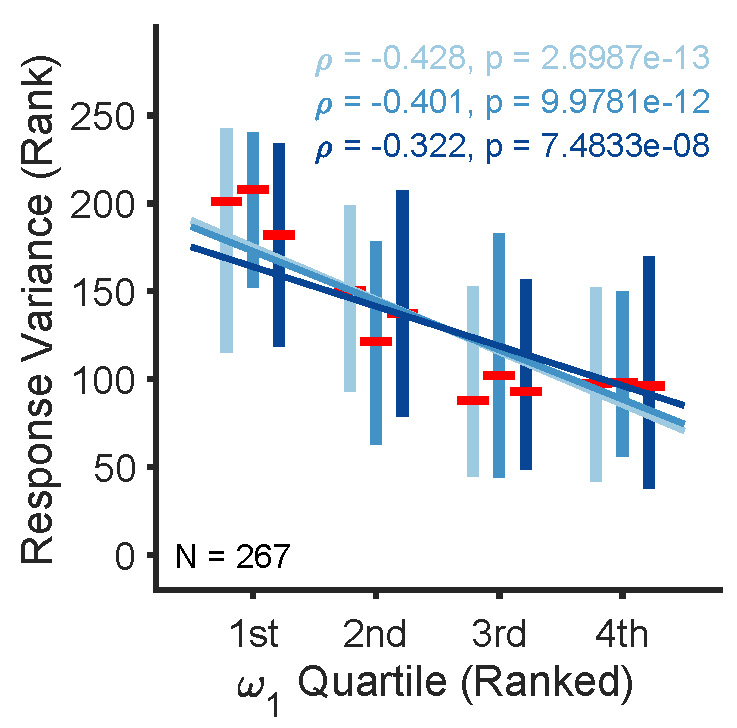


**S16 Fig. The relationship between the condition-wise response variance and** $\boldsymbol{\omega}_{\boldsymbol{1}}$**.** For visualization purposes, the data is presented as boxplots summarizing quartiles categorized by participant’s $\omega_{1}$ values. The box plots reflect the median (red line) and the 25% and 75% percentile. For each quartile, the condition-wise response variance data is presented for the 51:49 (light blue), 60:40 (medium blue), and 90:10 (dark blue) conditions. Overlayed on top of this is the least-squares linear fit for the rank-ordered relationship (using all of the data points, not quartiles) between the condition-wise response variance measures and $\omega_{1}$. The Spearman correlations for all three relationships are significant.
