# Supplementary material for: The effects of base rate neglect on sequential belief updating and real-world beliefs
Source: PLoS Comput Biol. 2022 Dec 22;18(12):e1010796. doi: 10.1371/journal.pcbi.1010796 (PMC9831339; doi:10.1371/journal.pcbi.1010796)
Supplement: S17 Fig — (DOCX) [file pcbi.1010796.s048.docx]

**
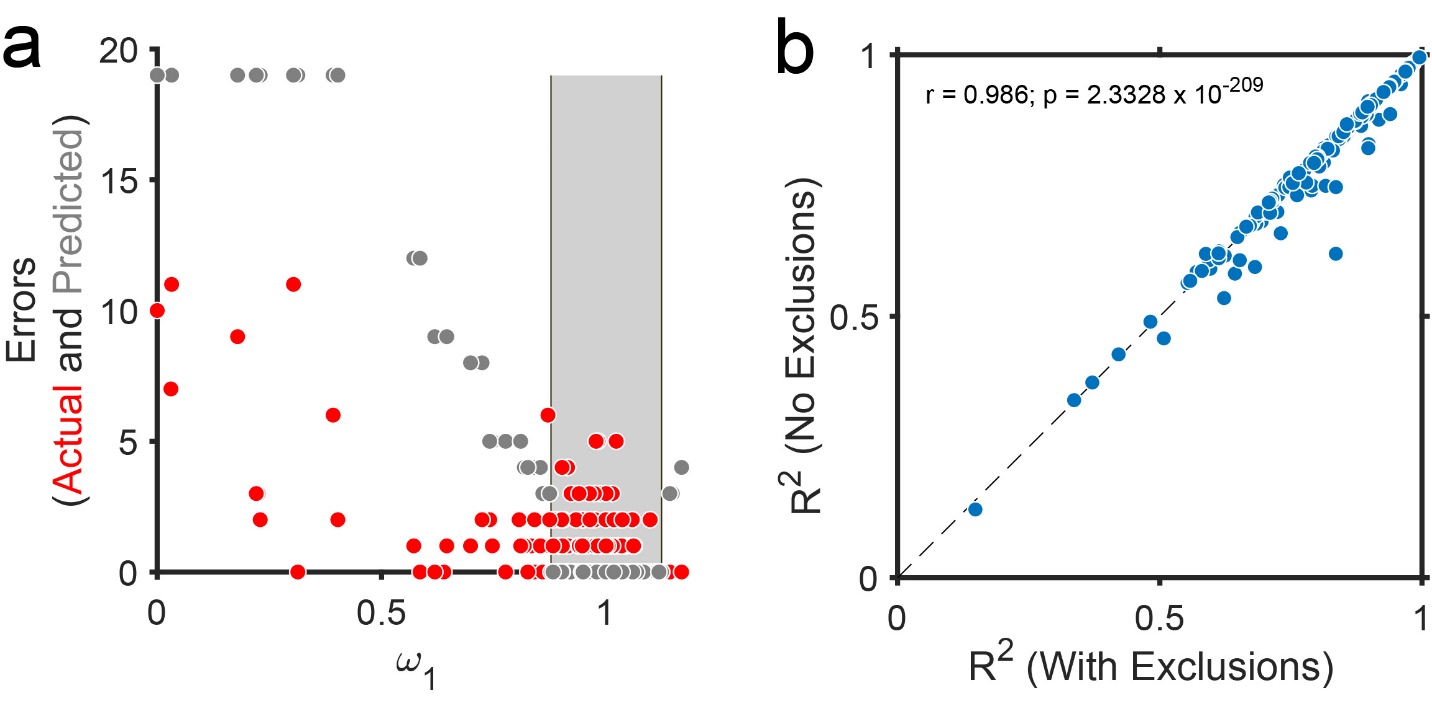
**

**S17 Fig. Trial-wise exclusions are justified because most box choice errors are not predicted by the weighted Bayesian model.**  In some circumstances, prior underweighting predicts biased beliefs, and subsequently choice errors. However, in our task, errors are only predicted (**a;** grey dots) in participants with either very low or very high $\omega_{1}$ values (substantial prior overweighting or underweighting). Based on the fitted data, the weighted Bayesian model predicts that 84% of participants (within the grey shaded region) should have 100% accuracy. Therefore, the majority of the observed box choice errors (**a;** red dots) are not predicted by the model and likely due to general inattention or disengagement. Consequently, we cannot assume the cognitive process of interest is at play in those moments, which justifies excluding incorrect-choice trials for the majority of participants. For the sake of consistency, we have elected to follow the same exclusion rules for all participants. Overall, trial-wise exclusions only exclude a small proportion of trials. The mean percentage of excluded trials was 1.5% per person, though the data was not normally distributed, and the median was 0.0%. Note that these analyses do not include the four trials with no clear answer (the 51:49 trials with 4 blue and 4 green beads) which we never excluded. To assess the impact of trial-wise exclusions, we fit the weighted Bayesian model to the data with and without applying the trial-wise exclusions. We found that the model goodness of fit was highly correlated **(b)** but was marginally better with exclusions**.** The model goodness of fit with exclusions was significantly larger than the model goodness of fit without exclusions (t = 1.76, p = 0.04; one-tailed paired t-test).
