# Supplementary material for: The effects of base rate neglect on sequential belief updating and real-world beliefs
Source: PLoS Comput Biol. 2022 Dec 22;18(12):e1010796. doi: 10.1371/journal.pcbi.1010796 (PMC9831339; doi:10.1371/journal.pcbi.1010796)
Supplement: S18 Fig — (DOCX) [file pcbi.1010796.s049.docx]

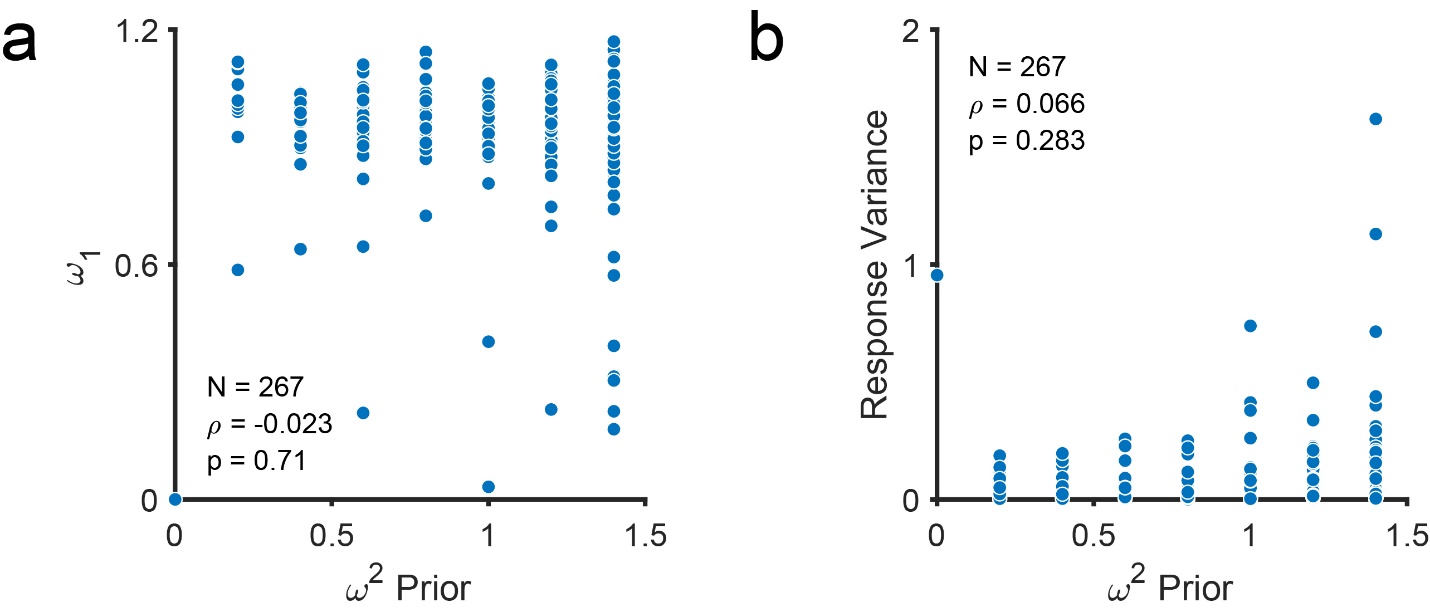


**S18 Fig. Negligible effects of** $\boldsymbol{\omega}_{\boldsymbol{prior}}^{\boldsymbol{2}}$ **on base-rate neglect and response variance.** One concern about the noisy-sampling model is that, since the $\omega_{prior}^{2}$ and $\sigma_{Prior}^{2}$ parameters induce similar effects, it is possible that sequential base-rate neglect effects observed in the data may in fact be driven by the assumed variance in the underlying logit prior, rather than noise in the internal representation of the prior. Using the 4-dimensional grid search described in the methods, we extracted best-fitting $\omega_{prior}^{2}$ parameter at the individual-subject level. We show that there is no relationship between the individually fitted $\omega_{prior}^{2}$ parameters and **(a)** $\omega_{1}$ from the weighted Bayesian model or **(b)** with response variance; a negative relationship with $\omega_{1}$ and a positive relationship with response variance would be predicted (S8 Fig) if the $\omega_{prior}^{2}$ parameter were driving the sequential base-rate neglect effects in our data. Further, we also calculated the sub-group fitted $\omega_{prior}^{2}$ parameter (i.e. the same 4-dimensional grid search across all participants in each group) for participants with high and low $\omega_{1}$ (high: $\omega_{prior}^{2}$ = 1.0 low: $\omega_{prior}^{2}$ = 0.2) and response variance (high: $\omega_{prior}^{2}$ = 0.2, low: $\omega_{prior}^{2}$ = 1.0), and found that these relationship between these parameters values were also inconsistent with model predictions; the model would predict higher $\omega_{prior}^{2}$ for the low $\omega_{1}$ group and the high response variance group. High and low group membership was determined based on a median-split. Taken together with S8 Fig, these results suggest that it is reasonable to fix the $\omega_{prior}^{2}$ and $\omega_{likelihood}^{2}$ parameters given that they are unlikely to play a significant role in the processes of interest.
