# Supplementary material for: The effects of base rate neglect on sequential belief updating and real-world beliefs
Source: PLoS Comput Biol. 2022 Dec 22;18(12):e1010796. doi: 10.1371/journal.pcbi.1010796 (PMC9831339; doi:10.1371/journal.pcbi.1010796)
Supplement: S1 Text — (DOCX) [file pcbi.1010796.s050.docx]

**S1 Text:** Supplemental Materials table of contents

**Sample Demographics**

**S1 Table:** Sociodemographic and clinical characteristics of samples included in data analysis.

**S2 Table:** Sociodemographic and clinical characteristics for study 1 comparing included participants to non-completers and excluded participants.

**S3 Table:** Sociodemographic and clinical characteristics for study 2 comparing included participants to non-completers and excluded participants.

**Introduction**

**S1 Fig:** Evidence asymmetry is unrelated to mean final estimates.

**S2 Fig:** Condition-wise simulations of the final estimate difference and prior-dependent updating as a function of $\omega_{1}$ and $\omega_{2}$.

**Results: Study 1**

**S3 Fig:** Probability estimates before presentation of the first bead (i.e., at the 0^th^ bead).

**S4 Table:** Linear mixed-effects model predicting probability estimates based on bead draw and bead ratio.

**S5 Table:** Linear mixed-effects model predicting probability estimates based on bead draw and bead ratio for matched trials.

**S6 Table:** Linear mixed-effects model predicting final estimate difference based on evidence asymmetry and bead ratio.

**S7 Table:** Linear mixed-effects model predicting mean logit-belief updates based on mean logit-priors and bead ratio.

**S8 Table.** Descriptive statistics for the parameters of the winning weighted Bayesian model for study 1, 2, and 3.

**S9 Table:** Pair-wise correlations between PDI score, the final estimate difference, the Evidence Asymmetry Slope, the prior dependent updating slope, and $\omega_{1}$.

**S4 Fig:** Comparison of sample’s general psychopathology factor scores to those in Gillan et al (2016).

**S10 Table:** Linear model predicting participant scores on the 9-item Raven’s Matrix based on their fitted parameters from the weighted Bayesian model (N = 143).

**S11 Table:** Linear model predicting participant scores on their anxious-depression factor score (S4 Fig) based on their fitted parameters from the weighted Bayesian model (N = 143).

**S12 Table:** Linear model predicting participant scores on their OCD factor score (S4 Fig) based on their fitted parameters from the weighted Bayesian model (N = 143).

**S13 Table:** Linear model predicting participant scores on their social withdrawal factor score (S4 Fig) based on their fitted parameters from the weighted Bayesian model (N = 143).

**Results: Study 2**

**S2 Text:** Direct replication of results from study 1 using Study 2 data.

**S14 Table:** Linear mixed-effects model predicting probability estimates based on bead draw and bead ratio for the main sample in study 2 (N = 91).

**S15: Table** Linear mixed-effects model predicting probability estimates based on bead draw and bead ratio for the main sample in study 2 (N = 91) for matched trials.

**S16 Table:** Linear mixed-effects model predicting final estimate difference based on evidence asymmetry and bead ratio for the main sample in study 2 (N = 91).

**S17 Table:** Linear mixed-effects model predicting mean logit-belief updates based on mean logit-priors and bead ratio for the main sample in study 2 (N = 91).

**S5 Fig:** Logit-belief updates as a function of logit prior by bead ratio for the main sample in study 2 (N = 91).

**S18 Table:** Linear mixed-effects model predicting final estimate difference based on evidence asymmetry and bead ratio for low PDI group only (N = 57).

**S19 Table:** Linear mixed-effects model predicting final estimate difference based on evidence asymmetry and bead ratio for the high PDI group only (N = 34).

**S20 Table:** Linear mixed-effects model predicting mean logit-belief updates based on mean logit-priors and bead ratio for the low PDI group only (N = 57).

**S21 Table:** Linear mixed-effects model predicting mean logit-belief updates based on mean logit-priors and bead ratio for the high PDI group only (N = 34).

**S22 Table:** Statistics for rank sum tests for group differences between Low (N = 34) and High (N =57) PDI groups for belief updating measures yielded by study 2.

**S23 Table:** Pair-wise correlations for study 2 between mean PDI score (mean of prescreening and experimental session PDI scores; see Methods), Paranoia Checklist score, the final estimate difference, the evidence asymmetry slope, the prior dependent updating slope, and $\omega_{1}$.

**S6 Fig:** Formal model comparison for data from (a) study 1 and (b) study 2.

**Results: Study 3**

**S7 Fig:** Predicted relationships between parameters governing prior integration and response variability in (a) a volatility model and (b) the noisy sampling model.

**S24 Table:** Correlations between measures of response variability and $\omega_{1}$ by individual study and for the full sample.

**S25 Table.** Descriptive statistics for the parameters of the noisy sampling model for study 3.

**S8 Fig:** Scatterplots and Spearman correlations of corresponding likelihood parameters from the weighted Bayesian model and noisy sampling model.

**S9 Fig:** Simulations varying individual $\omega^{2}$ parameters in the noisy sampling model, while holding all other parameters constant.

**S26 Table:** Statistics for rank sum tests for group differences between Low (N = 34) and High (N =57) PDI groups for belief updating measures yielded by study 3.

**S27 Table:** Prior weight $\omega_{1}$ is not associated with response times.

**Posterior Predictive Checks**

**S10 Fig:** Posterior predictive checks for mean final estimate difference and evidence asymmetry slope.

**S11 Fig**: Posterior predictive checks for logit belief updates.

**S12 Fig:** Posterior predictive checks for response variance.

**Methods**

**S13 Fig:** The binarized scoring rule maximizes expected value for accurate probability estimates.

**S1 Movie:** Video demonstration of task instructions, miscomprehension quiz, and practice trials.

**S14 Fig:** Data Quality 1: Comparison between the weighted Bayesian model mean squared error and total time taken to complete the probability estimates beads task.

**S15 Fig:** Data Quality 2: Evidence for behavioral consistency across the probability estimates beads task.

**S28 Table:** Weighted Bayesian belief updating models

**S29 Table:** PDI items and order of presentation of items.

**S30 Table:** Bead sequences (i.e., trials) used in studies 1 and 2.

**S31 Table:** Questions and possible responses during the miscomprehension quiz.

**S16 Fig:** The relationship between the condition-wise response variance and $\omega_{1}$.

**S17 Fig:** Trial-wise exclusions are justified because few box choice errors are predicted by the weighted Bayesian model.

**S18 Fig:** Negligible effects of $\omega_{prior}^{2}$ on base-rate neglect and response variance

**S3Text:** Volatility Model Specification

**Notation and Analysis Conventions**

1. For all analyses where reported: LL = Lower Limit and UL = Upper Limit
2. Unless otherwise noted, all linear mixed-effects models were conducted with random intercepts and slopes allowed for each participant and, to minimize type 1 errors, implemented the Satterthwaite correction for degrees of freedom[1].
3. For all linear mixed-effects models of logit beliefs probability estimates of exactly 1 and 0 were excluded to avoid numerical issues in logit space, where these values respectively convert to +∞ and -∞. This does not to apply to any of the model-fitting procedures, for which the cost function uses probability values.
4. Note that 2.2251e-308 was used as the precision limit in MATLAB.

References

1. Luke SG. Evaluating significance in linear mixed-effects models in R. Behav Res. 2017;49: 1494–1502. doi:10.3758/s13428-016-0809-y
