# Supplementary material for: The effects of base rate neglect on sequential belief updating and real-world beliefs
Source: PLoS Comput Biol. 2022 Dec 22;18(12):e1010796. doi: 10.1371/journal.pcbi.1010796 (PMC9831339; doi:10.1371/journal.pcbi.1010796)
Supplement: S2 Text — (DOCX) [file pcbi.1010796.s051.docx]

**S2 Text: Direct replication of results from study 1 using Study 2 data.**

As further validation of our task and model, we replicated the critical results from study 1 in the main sample of study 2, which consisted of 91 independent participants (Methods; supplemental results). Here, participants also reported increasing probability estimates for the true hidden box as the number of observed beads increased, particularly in conditions with stronger evidence across all trials (interaction of bead draw [0-8] by bead-ratio condition: t_93.26_ = 17.35, p = 5.8976 x 10 ^-31^, linear mixed-effects model; S14 Table) and the 16 identical trials (see Methods) across 60:40 and 90:10 bead-ratio conditions ([90:10]>[60:40]; interaction of bead draw [0-8] by bead-ratio condition: t_101.68_ = 8.42, p = 2.5303 x 10^-13^, linear mixed-effects model; S15 Table). As in study 1, participants in study 2 tended to exhibit a recency bias in model-agnostic measures (mean final estimate difference > 0: p = 1.6924 x 10^-5^; sign-rank test) which increased with evidence asymmetry (evidence asymmetry slope > 0; p = 9.0042 x 10^-9^; sign-rank test), with steeper slopes for stronger evidence ([90:10]>[60:40]>[51:49] bead-ratio conditions; bead ratio x evidence asymmetry interaction: t_77.99_ = 2.82, p = 0.006, linear mixed-effects model; combined data from Fig 4a and 4b; S16 Table). Further consistent with study 1 and the model predictions, the mean logit belief updates to prior-consistent evidence tended to decrease in magnitude as prior certainty increased (logit-prior main effect: t_95.74_ = -78, p = 0.007), again independent of evidence strength (logit-prior x bead ratio interaction: t_89.80_ = 0.1523, p = 0.88; S17 Table and S4 Fig; Combined data from Fig 4c and 4d).

The majority of the main model-based results also replicated: the same winning model as in study 1 was selected (S5 Fig), which included a prior-weight $\omega_{1}$parameter. Participants also tended to exhibit a base-rate neglect ($\omega_{1}<1$: p = 7.3592 x 10^-4^; sign-rank test) and interindividual variability in $\omega_{1}$ correlated with the model-agnostic measures of recency bias, the mean final estimate difference ($\rho= -0.55, p=2.8407 x {10}^{-8}$) and the evidence asymmetry slope ($\rho= -0.45, p=1.1852 x {10}^{-5}$; Fig 4e and 4f). Furthermore, prior-dependent updating – the slope of the logit belief update in the direction of the evidence as a function of the logit prior across all bead probability conditions positively correlated with $\omega_{1}$ ($\rho= 0.534, p=8.1704 x {10}^{-8})$ and negatively correlated with the mean final estimate difference ($\rho= -0.25, p= 0.019)$ and the evidence asymmetry slope ($\rho= -0.25, p= 0.017).$ Consistent with study 1 and the model predictions, these relationships held when controlling for the three $\omega_{2_{(likelihood)}}$ parameters, and the model RMSE (S23 Table).
