# Supplementary material for: The effects of base rate neglect on sequential belief updating and real-world beliefs
Source: PLoS Comput Biol. 2022 Dec 22;18(12):e1010796. doi: 10.1371/journal.pcbi.1010796 (PMC9831339; doi:10.1371/journal.pcbi.1010796)
Supplement: S3 Text — (DOCX) [file pcbi.1010796.s052.docx]

**S3 Text. Volatility Model.** In the main text, we describe simulating a volatility model, first described by Glaze et al.[1] This model is described by Eq. S1 and S2. In this model, $\psi\left( logit(Prior), H \right)$ reflects a weighted prior, which is governed by the hazard rate, H, which reflects the expected probability for each bead draw that the identity of the hidden box may have changed (i.e., from the mostly blue box to the mostly green box, or vice versa). When H is high, this implies the participant thinks a change in the hidden box is likely and induces base-rate neglect; the prior is incorporated into new beliefs less as hazard rate increases. We made one relevant change to the model, which is that we included a likelihood weight for each bead-ratio condition to make this model more directly comparable to the weighted Bayesian model and the noisy sampling model (which both included condition-specific likelihood weights). Free parameters for model fitting were H and $\omega_{2_{(likelihood)}}$ for each likelihood condition. In our simulations (Fig 6e, Fig 6f, and S6a Fig), simulated observers started each trial at uncertainty with regard to the correct box and the posterior estimate after each draw served as the prior for the subsequent draw.

$$\begin{aligned} logit(Posterior)= \psi\left( logit\left( Prior \right), H \right)+\omega_{2_{\left( likelihood \right)}}LLR \#Eq. S1 \end{aligned}$$

$$\begin{aligned} \psi\left( logit\left( Prior \right), H \right)= \\ logit(Prior)+\log\left[ \frac{1-H}{H} +\exp\left( -logit\left( Prior \right) \right) \right]-\log\left[ \frac{1-H}{H} +\exp\left( logit\left( Prior \right) \right) \right] \#Eq. S2 \end{aligned}$$

References

1. Glaze CM, Kable JW, Gold JI. Normative evidence accumulation in unpredictable environments. Behrens T, editor. eLife. 2015;4: e08825. doi:10.7554/eLife.08825
